# Dynamics of heavy chain junctional length biases in antibody repertoires

**DOI:** 10.1038/s42003-020-0931-3

**Published:** 2020-05-01

**Authors:** Kannan Sankar, Kam Hon Hoi, Isidro Hötzel

**Affiliations:** 1 Department of Antibody Engineering, South San Francisco, CA 94080 USA; 2 Department of Bioinformatics and Computational Biology, Genentech, South San Francisco, CA 94080 USA

**Keywords:** Clonal selection, VDJ recombination

## Abstract

Antibody variable domain sequence diversity is generated by recombination of germline segments. The third complementarity-determining region of the heavy chain (CDR H3) is the region of highest sequence diversity and is formed by the joining of heavy chain V_H_, D_H_ and J_H_ germline segments combined with random nucleotide trimming and additions between these segments. We show that CDR H3 and junctional segment length distributions are biased in human antibody repertoires as a function of V_H_, V_L_ and J_H_ germline segment utilization. Most length biases are apparent in the naive and antigen experienced B cell compartments but not in nonproductive recombination products, indicating B cell selection as a major driver of these biases. Our findings reveal biases in the antibody CDR H3 diversity landscape shaped by V_H_, V_L_, and J_H_ germline segment use during naive and antigen-experienced repertoire selection.

## Abstract (web summary)

Sankar et al. investigate the junctional length biases (determining antibody binding potential) as a function of germline gene usage in antibody repertoires. They show that CDR H3 and junction length are biased by V_H_, V_L_, and J_H_ germline segment usage and these biases are apparent in both naive and antigen-experienced repertoires but not in non-productive repertoires.

## Introduction

The diversity of sequences in the variable regions of immunoglobulins is the basis for the ability of these molecules to bind a virtually unlimited number of antigenic structures. Sequence diversity in the primary repertoire is created by recombination of germline segments for both the heavy and light chains, which results in the formation of full-length immunoglobulin variable region exons^1^. The light chain variable region is created by the joining of V_L_ and J_L_ germline segments while the V_H_ region is created by recombination of V_H_, D_H_, and J_H_ germline segments. The process of recombination starts with the heavy chain in progenitor B cells, initiated by D/J_H_ recombination followed by V_H_/DJ_H_ recombination^2,3^. Light chain recombination occurs in pre-B cells after successful completion of the heavy chain recombination. Germline segments in both chains are also trimmed and extended by a variable number of nucleotides by exonucleolytic nibbling of germline segments and random nucleotide incorporation in the N-regions flanking the D_H_ germline segment mediated by terminal deoxynucleotidyl transferase and germline segment palindromic duplications^3^. Self-reactive B cell clones with full-length, in-frame variable regions have V_L_ sequences replaced by receptor editing or are removed from the repertoire by apoptosis^4,5^. Cells passing this self-reactivity checkpoint form the naive B cell repertoire^5^.

The third complementarity-determining region (CDR) of the heavy chain (CDR H3) is the region of highest overall sequence and length diversity in antibody repertoires^1^. CDR H3 length approximates a Gaussian distribution^6^ and the average CDR H3 length varies as a function of species, age, isotype, B cell development stage and disease state^6–13^. CDR H3 amino acid composition is also biased in a CDR H3 length-dependent manner, associated with differential incorporation of D_H_ and J_H_ germline segment sequences of different lengths and sequence composition into CDR H3 of different lengths^6^. Beyond the germline segment biases, CDR H3 sequence biases that reflect underlying selective biases in B cell maturation have also been described. In particular, a bias towards shorter average CDR H3 lengths is observed in mature relative to immature B cells and in isotype-switched memory B cells relative to naive to B cells^9,10,14^. This is accompanied by a reduction of positively charged residue content and hydrophobicity within CDR H3 associated with negative selection of self-reactive clones in the repertoire^9,11,15,16^.

The analyses of CDR H3 diversity and biases in health and disease have been mostly performed independently of the V regions contributed by V_H_ and V_L_ germline segments^6–11,17–20^. Except for sequences that are directly incorporated into CDR H3, the impact of V germline segments on CDR H3 properties has neither been addressed nor expected. Analysis of the impact of the V_L_ on CDR H3 has been limited to properties of the third CDR of the light chain, which is closely associated with CDR H3, without any evidence of biases^14^. Finally, analysis of the impact of J_H_ germline segments on CDR H3 biases has been confined to the expected effects of differential J_H_ germline segment length and sequence composition^6^. A recent analysis of a large dataset of isotype-switched human antibody sequences with paired chain information revealed an unexpected preferential pairing of *IGHV3-7* (V_H_3-7) and Vκ2-30 germline segments^21^. This was determined upon further analysis to be linked with short CDR H3 length biases associated with both germline segments. This unexpected finding prompted us to undertake a high-dimensional analysis of CDR H3 sequences from several human antibody datasets to investigate the extent to which CDR H3 length might be biased by germline segment use in human immunoglobulin repertoires. Our results show several biases in CDR H3 and junctional length distributions associated with V_H_, V_L_, and J_H_ germline segment utilization that shape naive and antigen-experienced antibody repertoires in unexpected and unpredictable patterns.

## Results

### Sequence datasets

We analyzed sequences from four previously described B cell repertoire deep sequencing datasets including three donors each and a fifth dataset with eight donors (used here as an independent test dataset in most analyses to avoid over-representation of donors from a single source in combined data), referred to here as the TX, WA, CA, MA, and SRI datasets^20–25^. These represent the largest publicly available datasets with V_H_/V_L_ pairing (TX and CA) or V_H_-only information (MA, WA, SRI). The datasets were previously sequenced and bioinformatically parsed using a diversity of methods, minimizing the impact of methodological biases. A summary of the datasets including the number of donors, CD27 marker and isotype information has been summarized in Table 1 (see Supplementary Table 1 for details). Data was normalized by analyzing each donor individually and averaging within or among datasets to avoid over-representation of sequences from larger datasets. No antigen-specific selection of B cells was performed for any of the datasets, although the CA and MA datasets include both pre- and post-vaccination samples^21,22^. For simplicity we refer to the TX CD27^pos^ IgG/IgA, CA IgG, MA IgG/IgA, WA CD27^pos^, and SRI IgG subsets as TX AE, CA AE, MA AE, WA AE, and SRI AE, respectively (with “antigen-experienced”, or “AE”, encompassing all five), the TX CD27^pos^ IgM sequences as “AE IgM” and the TX and WA CD27^neg^ subsets as TX naive and WA naive respectively (with “naive” encompassing both, Supplementary Table 1). SRI IgM sequences with no amino acid somatic mutations between Cys-23 and Cys-104 (International ImMunoGeneTics, IMGT^®^, numbering system^26^ used throughout) including most of the region covered by reads are referred as SRI naive.

**Table 1:** Summary of datasets used for analysis.

	Dataset				
	CA	MA	TX	WA	SRI
Reference	21	22,24^a^	23	25	20
Donors	3	3	3	3	8
CD27 marker	NA^b^	NA	Available^c^	Available	NA
Isotypes	IgG	IgG/IgA	IgG/IgA/IgM^d^	NA	IgG/IgM
Chain	V_H_/V_L_^e^	V_H_	V_H_/V_L_^e^	V_H_	V_H_
Unproductive sequences	NA	NA	NA	Yes^f^	NA

^a^Illumina re-sequencing data (24) of the original MA dataset samples (22) was used.

^b^Not available.

^c^Two donors with CD27^pos^ and three donors with CD27^neg^ B cells.

^d^Isotype information ignored for CD27^neg^ IgM clones in the TX dataset.

^e^V_H_/V_L_ variable region sequence paring information available.

^f^CD27^neg^, frameshifted sequences used only.

We aimed at identifying properties shared among donors not influenced by clonal expansion. To minimize the impact of clonal expansion, all datasets except nonproductive sequences were processed to retain a single sequence from each lineage, or clonotype, by clonotype clustering, according to germline segments as well as CDR H3 length and sequence similarity (see ‘Clonotype clustering’ under Methods and Supplementary Data 1, 2, and 3 for unique sequence counts after clustering). Overall distribution of CDR H3 lengths was not noticeably affected by removal of redundant sequences in most datasets except for the WA AE and MA AE compartments, which had subtle shifts (Supplementary Fig. 1a-f). The overall AE CDR H3 length distributions are similar among datasets except SRI, allowing pooling data of AE B cell subsets from different datasets (Supplementary Fig. 1g). However, the relative CDR H3 length distributions of the WA and TX naive B cell subsets differed by an average of 0.9 residues (Supplementary Fig. 1h) and were analyzed separately. Germline segment-specific analyses were performed with germline segments with at least 80 unique clonotypes in a donor, which, in aggregate, include 94–99% of the unique clonotype sequences in the CA, TX, MA and SRI datasets (Supplementary Data 1, 2, and 3). Germline segment-specific analyses in the WA dataset were performed with 16 V_H_ germline segments that had fewer than 10% ambiguous calls in the naive subset and germline segments prevalent in other datasets, totaling about one third of the entire dataset (Supplementary Data 4).

### Average CDR H3 length varies with V_H_ and V_L_ germline segment use

**Fig. 1:**
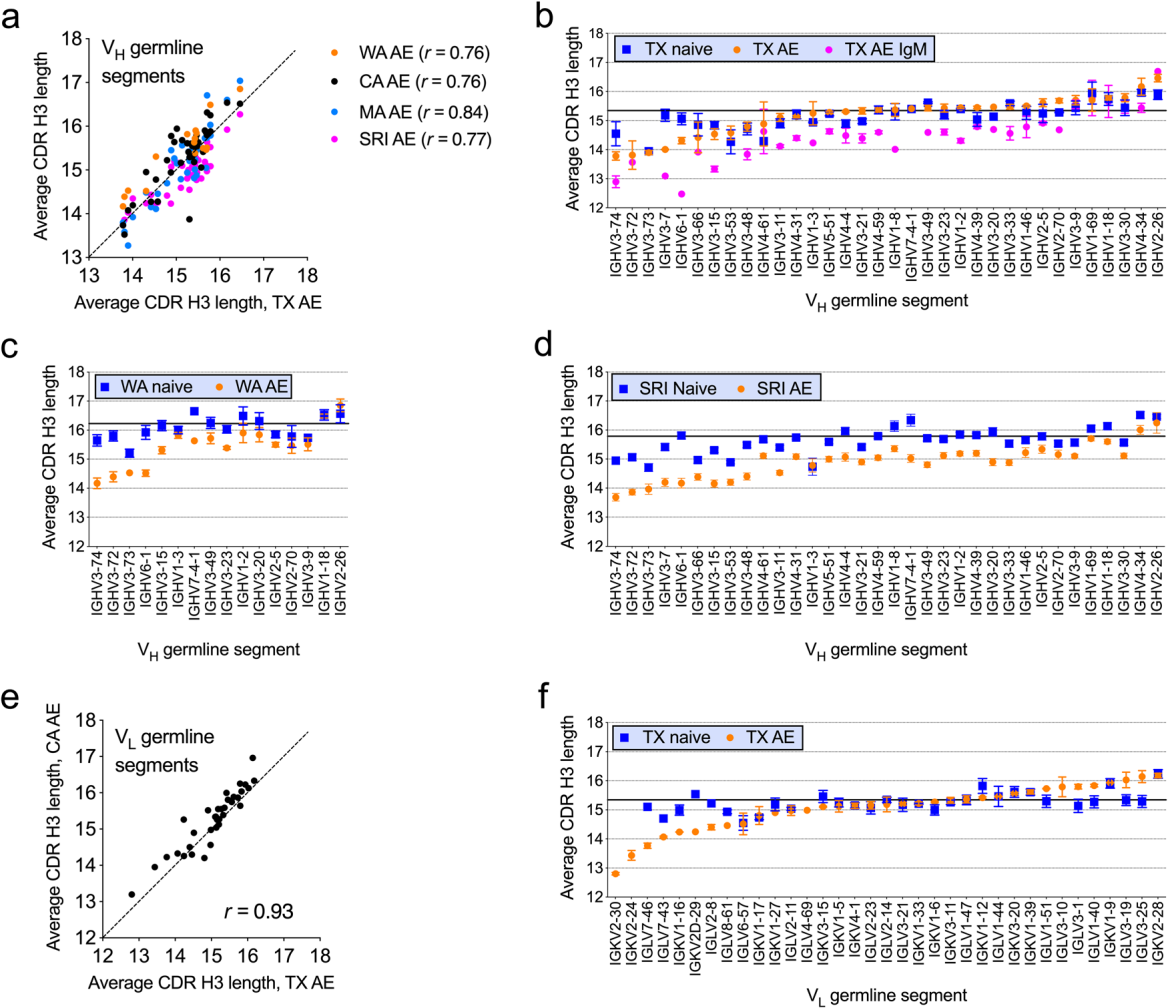
Average CDR H3 length associated with V_H_ and V_L_ germline segments. Average CDR H3 lengths associated with different germline segments in the TX AE dataset (abscissa) correlated with the CDR H3 associated with V_H_ germline segments of different datasets (**a**) and CA V_L_ germline segments (**e**) in the AE compartments. Each dot represents the average CDR H3 length for a different V_H_ or V_L_ germline segment within each dataset, averaged across donors within each dataset. Diagonal lines show 1:1 length correspondence between datasets, with Spearman’s correlation coefficients shown. **b**–**d** Average CDR H3 length associated with V_H_ germline segments in the TX AE, TX AE IgM, WA AE, SRI AE and corresponding naive B cell datasets. Solid horizontal bars indicate average CDR H3 length in the naive B cell compartment. The order of germline segments is the same in the panels. **f** Average CDR H3 length associated with V_L_ germline segments in the TX AE and naive B cell datasets. Solid horizontal bars indicate average CDR H3 length in the naive B cell compartments. Error bars indicate S.E.M. for *n* = 3 donors or range for *n* = 2 donors. Germline segments without error bars have only 1 donor with at least 80 sequences (TX dataset, see Supplementary Data 1, 2 and 3) or sequences mostly represented by 1 donor (WA, see Supplementary Data 1 and 2). Lengths are shown in amino acid residues according to the IMGT® CDR definition^26^.

We analyzed average CDR H3 length by V_H_ or V_L_ germline segment use. Average CDR H3 length in the AE subset varied by up to 3 amino acid residues as a function of V_H_ germline segment use and correlated well for different datasets when compared to the TX dataset (Spearman’s *r* = 0.72–0.84) (Fig. 1a). Average CDR H3 length also varied as a function of V_L_ germline segment use by up to four amino acid residues in the AE compartment and correlated well between the CA and TX datasets (Spearman’s *r* = 0.93) (Fig. 1e). The naive compartment showed a more limited spread in average CDR H3 lengths relative to the AE compartment (Fig. 1b, c, d and f, blue squares). More pronounced reductions in average CDR H3 length in the AE relative to naive compartments were associated with a subset of V_H_ and V_L_ germline segments (Fig. 1b, c, d and f). The TX AE IgM subset showed similar trends as the TX AE subset except that average CDR H3 length was decreased in association with most V_H_ germline segments relative to the naive compartment (Fig. 1b, pink dots).

### CDR H3 length distribution varies with V_H_ germline segment use

**Fig. 2:**
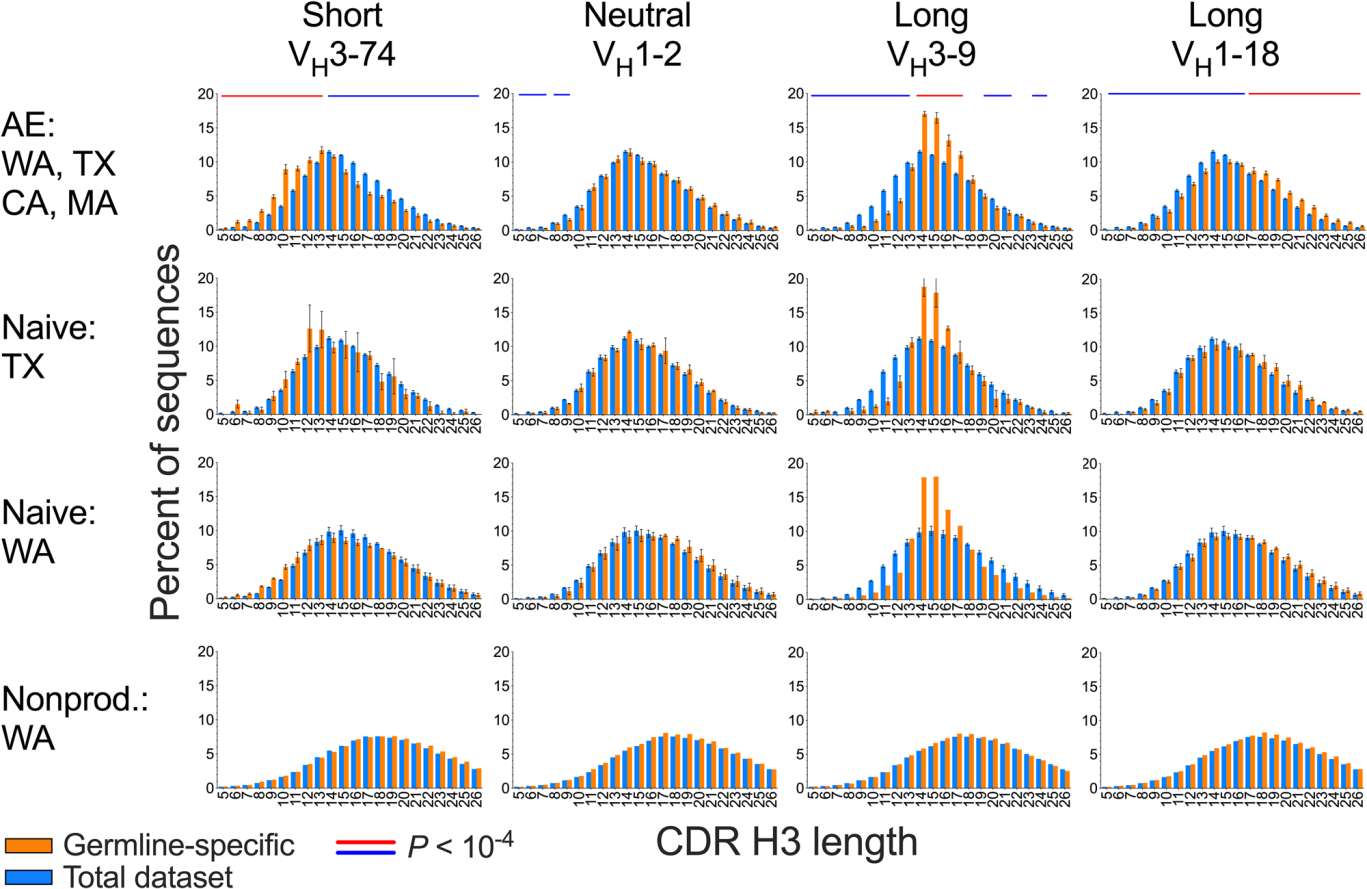
CDR H3 length distribution groups associated with V_H_ germline segments. Characteristic examples of each V_H_ bias group are shown, averaged for TX, CA, MA and WA donors in the AE compartment (top row, *n* = 11 donors), TX naive (second row, *n* = 3 donors), WA naive (third row, *n* = 3 donors) and WA nonproductive compartments (bottom row, three pooled donors). Orange bars are germline segment-specific CDR H3 length distributions of unique clonotypes. Blue bars are overall CDR H3 length distributions of unique clonotypes. Blue and red horizontal lines above the distributions indicate range of CDR H3 lengths statistically significant different between germline segment-specific and overall length distributions in a two-tailed paired *t*-test (*P* < 10^−4^) with a sliding window of two contiguous CDR H3 lengths, with red and blue indicating relative enrichment and depletion in the germline segment-specific distributions. Distributions were determined for each donor individually followed by averaging across donors, except for the nonproductive sequences in which donors were combined prior to calculation of distributions. Error bars indicate S.E.M. The full set of distributions is shown in Supplementary Fig. 2.

We next determined whether CDR H3 length distribution varies with germline segment use. Overall and germline segment-specific CDR H3 length distributions were performed individual for each donor and the frequency of each CDR H3 length averaged within or across the TX, CA, MA, and WA datasets (11 donors), with the eight donors from the SRI dataset analyzed separately. Overall CDR H3 length distribution of the respective B cell compartment, which is influenced by germline segment frequency in the datasets, was used as a relative standard to which germline segment-specific CDR H3 length distributions were compared. This was done to facilitate comparison of biases between germline segments and also because useful objective reference distributions are not available to determine bias types in naive compartment sequences. Therefore, most biases described here, including all in the naive compartment, are relative to the entire set of clonotypes in each B cell compartment. Statistical analysis of biases was performed in the AE compartment by a two-tailed paired (by donor) *t*-test of length frequencies with a sliding window of two consecutive CDR H3 lengths to minimize the impact of local fluctuations. Observed length distribution biases included overall shifts in average CDR H3 length for sequences with different V_H_ germline segments and also obvious and subtle deviations from the overall CDR H3 distribution in discrete ranges of the length spectrum (Fig. 2, top row, Supplementary Fig. 2a).

**Fig. 3:**
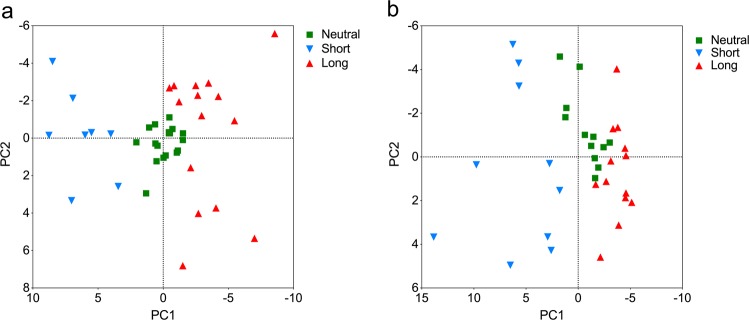
Differentiation of CDR H3 length distributions by PC analysis. PC analyses of V_H_ (**a**) and V_L_ (**b**) germline segment-associated CDR H3 length distributions in the AE compartments are shown. Analysis in **a** excludes the WA dataset due to limited germline segment coverage. Analysis in **b** includes both CA and TX datasets. Each data point indicates a germline segment for which minimum count requirements were met for the analysis. Points are color-coded by bias group determined by visual inspection of CDR H3 length distributions (Supplementary Fig. 2 and 6). Axes are oriented to position distributions skewed towards long lengths and with high apparent kurtosis to the right and top respectively.

To further discern the CDR H3 length biases quantitatively, we performed a principal component (PC) analysis of the length distributions (lengths 5–26) associated with different V_H_ germline segments. Results from the PC analysis were visualized by projecting each germline segment onto the most important trends to obtain the so-called PC scores, aided by a visual analysis of the corresponding distributions (Fig. 3a). PC1 and PC2 corresponded to apparent skewness and kurtosis of the distributions, respectively. Using the PC analysis results in conjunction with visual inspection of V_H_ germline segment-associated CDR H3 distributions in the AE compartment, germline segments were categorized by bias type as “Short”, “Neutral” and “Long” (Figs. 2 and 3a, Supplementary Fig. 2). Those germlines that had similar length distributions as the overall distribution were called “Neutral” (located around the center of the PC plot); while those with shifts towards longer or shorter lengths as “Long” (right-skewed, with low values of PC1) and “Short” (left-skewed, with high values of PC1) respectively. Within each of these classes, some germlines also showed varying degrees of kurtosis relative to the overall distribution (extreme values in PC2). Differences between the distributions of members of different groups can be subtle, both visually and in the PC analysis. The magnitude of the biases and the details of distribution shapes varied for different V_H_ germline segments within each group but were consistent across datasets for each germline segment (Supplementary Fig. 3). Germline segments in the same V_H_ subfamily did not always have the same biases. The range of germline segment prevalence in the various datasets was similar for the different bias groups (Supplementary Fig. 4a).

We determined whether the observed distribution biases were also present in the naive B cell subset. The biases of the Long group were also observed in the naive B cell compartment, without apparent differences relative to the distributions in the AE compartments (Fig. 2, Supplementary Fig. 2). All the germline segments in the Neutral group showed average CDR H3 length distribution in the naive subset as well. However, distribution biases of the Short group in the naive compartment were less prominent (Fig. 2 and Supplementary Fig. 2), consistent with the average CDR H3 length analysis (Fig. 1b and c). Short biases in the naive compartment were mostly limited to the V_H_3-73 and V_H_3-15 germline segments in the TX and WA datasets. Despite the differences in overall CDR H3 length between the TX and WA naive datasets, the biases in the naive compartment had the same trends in both datasets (Fig. 2 and Supplementary Fig. 2).

The data analysis was performed with datasets filtered for sequences likely to belong to the same lineage. To confirm that biases are not due to pockets of clonal expansion, we performed a repertoire similarity index (RSI) analysis with the CA, TX and MA datasets similar to a recently described method^27^ (see Methods for more details). Overall, no apparent increase in RSI scores indicating clonal expansion was associated with regions of positive prevalence biases in different parts of the CDR H3 length spectrum for the different bias groups (Supplementary Fig. 5a), confirming that clonal expansion does not account for the observed CDR H3 length biases.

### CDR H3 length distribution biases are not generated by recombination

We next determined whether the biases observed in the naive compartment are a direct consequence of biases in the VDJ recombination process for each germline segment. For this, we analyzed frameshifted, nonproductive V_H_ sequences that were part of the naive WA dataset. Nonproductive recombination products are not directly subject to selection and therefore provide information about recombination products prior to any repertoire selection. As previously reported^16^, the CDR H3 lengths of nonproductive V_H_ genes are longer than the productively recombined genes in mature B cell subsets (Fig. 2 and Supplementary Fig. 2). However, CDR H3 length for the nonproductive sequences associated with different V_H_ germline segments approximated a Gaussian distribution, with no observable biases associated with different V_H_ germline segments relative to the overall dataset, except for minor anomalies associated with some germline segments (Fig. 2 and Supplementary Fig. 2). Therefore, heavy chain recombination mechanisms do not account for the naive repertoire CDR H3 length distribution biases.

### CDR H3 length distribution varies with V_L_ germline segment use

**Fig. 4:**
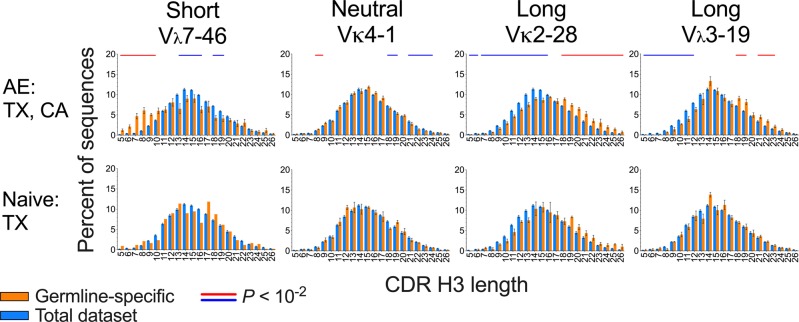
CDR H3 length distribution groups associated with V_L_ germline segments. Characteristic examples of each group are shown, averaged for all CA and TX donors in the AE compartments and the naive compartment of TX donors. Blue and red horizontal lines above the distributions indicate range of CDR H3 lengths statistically significant different between germline segment-specific and overall length distributions in a two-tailed paired (within donor, *n* = 5 donors) *t*-test (*P* < 10^−2^) with a sliding window of two contiguous CDR H3 lengths, with red and blue indicating relative enrichment and depletion in the germline segment-specific distributions. Number of donors is 5 for TX AE and CA AE combined and 3 for TX naive except for V_λ_7-46 in TX naive, where *n* = 1. Distributions were determined for each donor individually followed by averaging across donors. Error bars indicate S.E.M. The full set of distributions is shown in Supplementary Fig. 6.

We performed a similar analysis of CDR H3 length distribution as a function of V_L_ germline segment and B cell compartment using PC and visual analysis. Similar to the V_H_ germline segment-associated biases, V_L_-associated biases in the AE compartment could be classified into three groups based on the skewness of their distributions, named here “Short” (right-skewed, with high value of PC1), “Long” (left-skewed, with low value of PC1) and “Neutral” (intermediate values of PC1), present in both the CA and TX datasets, each group including a diverse set of Vκ and V_λ_ germline segments (Figs. 3b and 4, top row, and Supplementary Fig. 6). PC1 and PC2 for the light chain were also associated with apparent skewness and kurtosis. The V_L_ Long bias group has Gaussian CDR H3 length distributions, whereas the V_L_ Short bias group includes distribution shapes with marked deviations from Gaussian, including localized frequency spikes in discrete sections in the short range. Only Vκ germline segments in the Long group were associated with similar CDR H3 length biases in the TX naive compartment (Fig. 4, Supplementary Fig. 6). The magnitude of the V_L_-associated biases varied for different germline segments within each bias group but were consistent between datasets (Supplementary Fig. 7). As above, the RSI analysis results indicated that clonal expansion does not account for the V_L_ germline segment-associated CDR H3 length biases (Supplementary Fig. 5b). The prevalence of Short group germline segments in the dataset was lower than for germline segments of the other two groups (Supplementary Fig. 4b).

### CDR H3 length is biased as a function of V_H_/J_H_ combination

J_H_ germline segments vary in the number of amino acid residues that can be potentially contributed to CDR H3 from 4 in J_H_4 to 9 in J_H_6. We assessed whether differential J_H_ germline segment usage as a function of V_H_ and V_L_ germline segment use is the basis for V segment-associated CDR H3 length biases. No clear deviations from average J_H_ usage were observed in association with most V_H_ germline segments in the WA unproductive sequences (Supplementary Fig. 8a and 9, top panel). The observed deviations in J_H_ prevalence do not readily explain CDR H3 distribution biases associated with V_H_ and V_L_ germline segments (Supplementary Fig. 8b, c and d, and Supplementary Fig. 9, bottom panel) with the exception of V_L_ germline segment Vκ2-28 in the Long CDR H3 bias group, which was associated with a higher prevalence of the longer J_H_6 and lower prevalence of the shorter J_H_4 germline segments in the heavy chain (Supplementary Fig. 8d).

**Fig. 5:**
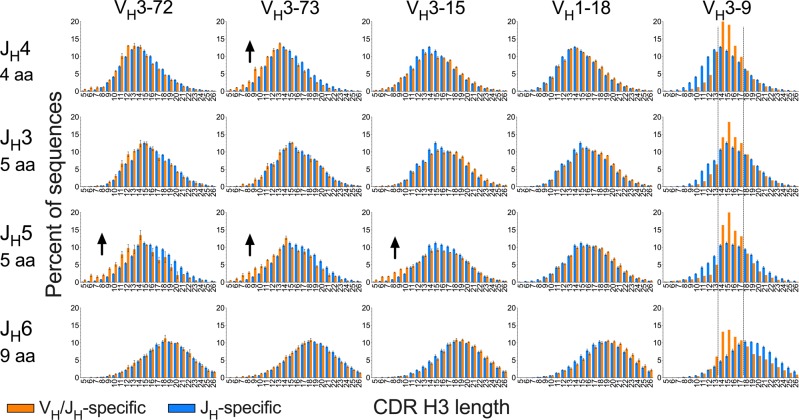
Modulation of V_H_-associated CDR H3 length biases by J_H_ germline segments in the WA naive compartment. CDR H3 length distributions associated with individual V_H_/J_H_ combinations (orange bars) are compared to overall repertoire distributions associated with J_H_ germline segments (blue bars). Arrows highlight biases dependent on J_H_ segment use. Dotted lines indicate the of peaks in the distributions associated with V_H_3-9 and all J_H_ germline segments in the same part of the length spectrum. The number of CDR H3 amino acid (aa) residues potentially encoded by the J_H_3 to J_H_6 germline segments is indicated in row legends. Distributions were determined for each donor individually followed by averaging across donors. Error bars indicate S.E.M. (*n* = 3). The full set of distributions is shown in Supplementary Fig. 10. The figure panels are ordered vertically by the number of residues the J_H_ segments can contribute to CDR H3.

We next analyzed CDR H3 length distributions associated with different V_H_/J_H_ germline segment combinations, comparing these to the CDR H3 length distribution of all sequences with the corresponding J_H_ germline segment. As expected, CDR H3 length distributions were generally shifted according to length of the J_H_ segment in the germline regardless of V_H_ germline segment (Fig. 5, Supplementary Fig. 10 and 11). However, a subset of V_H_-associated CDR H3 length biases were impacted by J_H_ germline segment in a manner independent of length of the J_H_ segment in the germline, with very similar patterns in the naive WA and naive SRI subsets (Fig. 5, Supplementary Fig. 10 and 11). These included a short CDR H3 length bias associated with sequences with the V_H_3-72, V_H_3-73, and V_H_3-15 germline segments combined with the J_H_5 and/or J_H_4 germline segments (Fig. 5, Supplementary Fig. 10 and 11). Additional CDR H3 length biases were observed for other V_H_/J_H_ germline segment combinations (Supplementary Fig. 10). Our results indicate that CDR H3 length distribution biases are not necessarily uniform for each V_H_ germline segment but may vary in association with J_H_ germline segment. In addition, the effect of J_H_ on CDR H3 length distribution is not necessarily similar within V_H_ bias groups, indicating some degree of heterogeneity within bias groups.

### Differentially trimmed J_H_ segments in the naive compartment

**Fig. 6:**
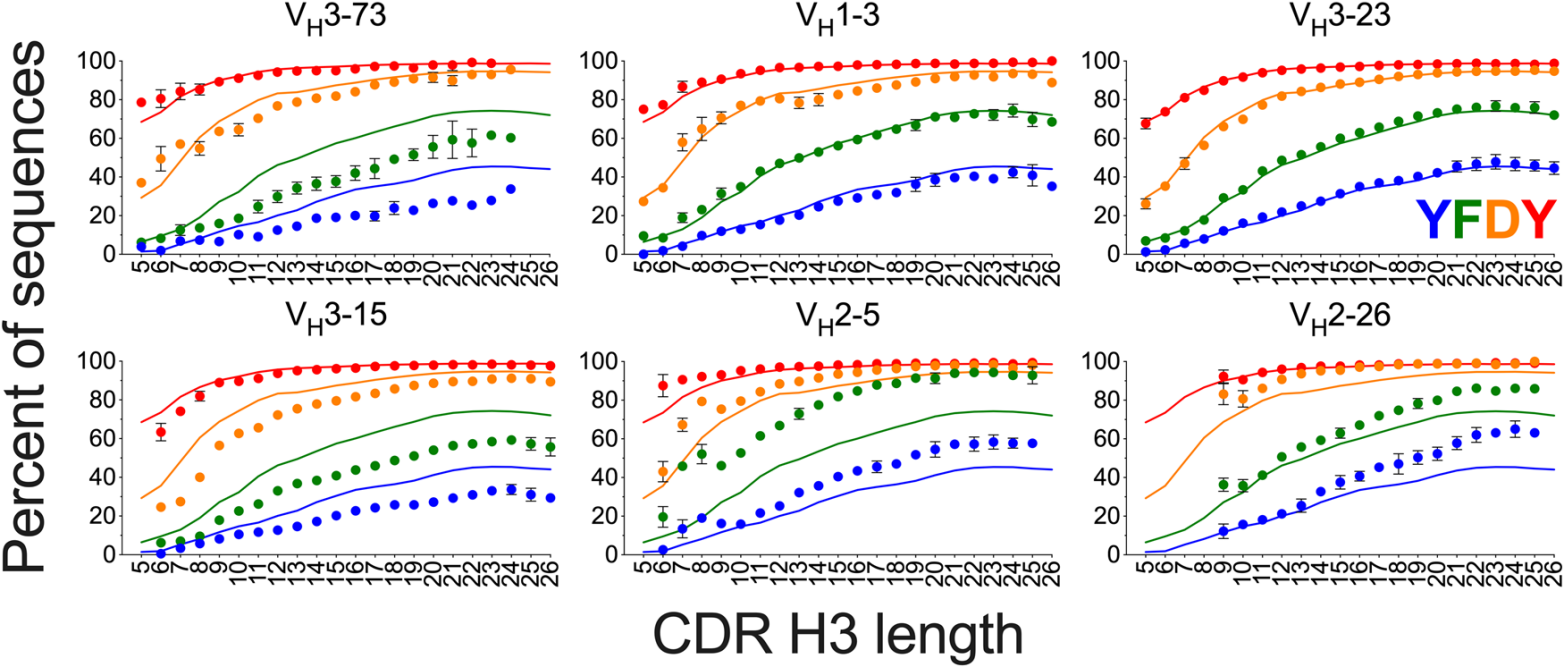
Biases in J_H_4 germline segment residue occupancy in CDR H3 associated with different V_H_ germline segments and CDR H3 length in WA naive sequences. Occupancy of J_H_ germline segment-encoded residues in the last CDR H3 positions (IMGT® 114–117) is shown. J_H_4 residues are color-coded by position as indicated in the V_H_3-23 panel by “YFDY”, the four J_H_4 residues that can be included in CDR H3 in positions 114–117. Solid lines indicate average residue occupancy for all sequences in the naive repertoire with the J_H_4 germline segment. Dots indicate average residue occupancy with each V_H_ germline segment and CDR H3 length. Bars indicate S.E.M. (*n* = 3 donors). Data points with fewer than 80 sequences were excluded.

The CDR H3 length distribution biases associated with a subset of V_H_/J_H_ germline segment combinations may be a consequence of biases in J_H_ trimming as a function of V_H_ germline segment. J_H_ residue occupancy in the last CDR H3 positions of J_H_4 and J_H_5 sequences was used to indirectly determine J_H_ trimming. The J_H_1, 2, 3, and 6 germline segments were not analyzed due to lack of sufficient data or, in the case of J_H_6, limited CDR H3 length biases associated with it. No apparent biases in J_H_ residue occupancy relative to the overall dataset was observed for any of the analyzed V_H_/J_H_ combinations in the nonproductive WA sequences (Supplementary Fig. 12). However, J_H_ residue trimming biases were observed for different V_H_/J_H_ combinations in the WA naive compartment (Fig. 6 and Supplementary Fig. 12). General trends in residue occupancy in J_H_4 were similar in SRI naive sequences for the V_H_/J_H_4 germline segment combinations with sufficient numbers for analysis (Supplementary Fig. 13). Residue-specific trimming biases were mostly coordinated for consecutive J_H_ residues in each analyzed V_H_/J_H_ combination, as expected due to the directional nature of trimming. However, closely related V_H_ germline segments can be associated with distinct trimming biases of different J_H_4 residues. For instance, V_H_2-5/J_H_4 sequences are associated mostly with reduced trimming of IMGT^®^ residue 115 (Phe) whereas for V_H_2-70/J_H_4 strongly reduced trimming of residue 116 (Asp) was also observed (Fig. 5 and Supplementary Fig. 12). The results indicate a complex set of constraints leading to selection of differentially trimmed J_H_ segments in the context of certain V_H_ and J_H_ germline segments during naive repertoire maturation.

## Discussion

Understanding antibody CDR H3 diversity generation, a process critical for the availability of immune receptors binding a wide range of antigens, has long been a goal in the immunology and antibody engineering fields. Numerous reports have described overall CDR H3 length and amino acid composition biases in health and disease and in different B cell developmental stages^6–20^. CDR H3 and junctional segments lengths in the B cell repertoire have been assumed to be independent of V_H_ and J_H_ germline segments except for their lengths prior to recombination^17,19,20^. This assumption has been implicitly used, for example, to generate simulated CDR H3 datasets to estimate the significance of observed clonal convergence in repertoires^20^. Analysis of the impact of V_L_ on CDR H3 length has been limited to CDR L3 length, with negative results^23^. Here we describe detailed, high-dimensional analyses of CDR H3 and junctional segment length distributions and show a complex set of biases determined by V_H_, V_L_, and J_H_ germline segment use and B cell maturation state that are not predictable from germline segment lengths and amino acid sequences. Most of the length and junctional biases we describe are evident in the naive B cell compartment but not in the nonproductive subset, indicating a major role of naive B cell repertoire maturation and, by extension, selection against self-reactivity or for structural integrity as likely factors in shaping those biases. In addition, only a subset of V_H_ or V_L_ germline segments is associated with biases towards shorter CDR H3 lengths in the antigen-experienced compartment, indicating general germline segment-specific adaptive immunity selection processes shared among individuals. Similar T cell receptor β chain CDR3 length distribution biases with different *TRBV* germline segments in repertoires arise in the process of T cell maturation^28^, although selective processes may differ between B and T cell repertoires due to differences in mechanisms of antigen recognition. Our results provide a detailed view into the dynamics of heavy chain junctional biases in antibody repertoires that complements previously described dynamics of clonal selection and expansion, convergence, sequence diversity and changes in overall CDR H3 properties in B cell maturation^9–11,20,22,25,27,29^.

Special consideration was given to the repeatability and robustness of the findings. The results are based on a total of 12 donors in four datasets and confirmed by analysis of 8 additional donors from the SRI dataset. These datasets were obtained and parsed with different sequencing methods and bioinformatic pipelines, minimizing the impact of technical artifacts. Some of the biases, such as those associated with V_L_, and J_H_ cannot be easily generated by sequencing or parsing artifacts, especially in a systematic fashion across datasets. The stringency of clonotype clustering criteria had limited impact on results. This is exemplified by the WA and SRI datasets, which yielded CDR H3 length distributions similar to other datasets (Supplementary Fig. 3) despite having been clustered by clonotype using a higher CDR H3 sequence identity threshold than other datasets (Supplementary Table 1).

A recurring theme in the results presented here was that biases observed at one level (e.g., V_H_ germline segment) were only partly explained by biases at higher-dimensional levels (e.g., V_H_/J_H_ combinations), with additional unexpected biases observed in the higher-dimensional levels. It is expected that higher-dimensional analyses including other repertoire descriptors will uncover additional biases, two examples being D_H_ junctional length biases (Supplementary Fig. 14, 15, and 16 and Supplementary text) and CDR H3 length biases associated with different V_H_ germline segment allelic variants (Supplementary Fig. 17). Haplotype variations could also potentially affect CDR H3 length distributions in a V_H_ allele-dependent manner or through differences in D_H_ germline segment composition and differential recombination frequencies of D_H_ or J_H_ germline segments of different lengths in different chromosomes, combined with differential recombination frequencies of V_H_ alleles^30–32^. However, the observation of essentially the same CDR H3 length distribution biases in several donors from 5 different sources and junctional segment length biases in 11 donors from 2 of these sources, along with a lack of systematic associations between V_H_, D_H_, and J_H_ alleles across donors^31,32^, indicates that haplotype variations are unlikely to be a major factor in the CDR H3 and junctional length distribution biases described here. In addition, heavy chain variable region haplotype differences would not be expected to impact CDR H3 distributions associated with V_L_ germline segments and the AE compartment-specific short CDR H3 length biases.

The analyses shown here use germline segment information as a proxy for undefined sequence features that ultimately determine the observed biases. The selected CDR H3 sequence and structural properties that result in the observed biases and the germline segment sequence properties that determine those biases remain to be identified. Analysis of V_H_ germline segment residues that can directly encode or bias the first CDR H3 residues in IMGT^®^ positions 105–107 did not reveal clear correlations between these and most CDR H3 bias groups or junctional segment length biases (Supplementary Fig. 18). In addition, no obvious correlations between J_H_ trimming biases and variations in V_H_ germline segment residues in positions 40–42 generally contacting the differentially trimmed J_H_ residues 115 and 116 were observed. The differentially trimmed residue 116 is located in a region at the base of CDR H3 that can adopt either a “bulged” or “extended” conformation^33,34^. The bulged conformation appears to depend on the Ig domain, encoded mostly by V_H_ germline segments^34,35^. Whether V_H_ germline segment-dependent J_H_ trimming biases reflect biases in the structure of the CDR H3 base remains to be determined. One challenge in determining how different germline segment regions determine the observed biases is the relatively limited number of non-redundant human antibody structures with different V_H_/J_H_ combinations or V_L_ germline segments with different CDR H3 lengths.

The CDR H3 biases described here pose questions about the functional properties that might shape those biases and the functional consequences of these biases for adaptive immunity. The emergence of some biases in the naive repertoire suggests selection against self-reactivity, selection for structural integrity, expression or a combination of these factors as possible mechanisms. If related to selection against self-reactivity, the different biases indicate either that features other than CDR H3 charge and hydrophobicity contribute to self-reactivity or that V germline segments outside CDR H3 modulate the self-reactivity mediated by these factors. The possible role of selection against self-reactivity may allow leveraging the biases observed in CDR H3 in large natural human antibody repertoire deep sequencing datasets to understand CDR H3 properties beyond charge that correlate with antibody polyspecificity and self-reactivity, a subject of considerable interest in therapeutic antibody development and in the understanding of functional consequences of immune disorders with altered CDR H3 sequence profiles^11,36,37^. The bias towards shorter CDR H3 lengths associated with a subset of V_H_ and V_L_ germline segments in the AE compartment may be attributable to these same mechanisms or to antigen-driven immune selection. The latter would suggest widespread convergences in human repertoires associated with certain V_H_ and V_L_ germline segments or, possibly, some degree of functional specialization in the germline segment repertoire linked to short CDR H3 sequences, analogous to the association between CDR H3 length and recognition of different antigen classes^38^.

Our results point to unexpected cross-constraints between V_H_, V_L_, J_H_, and other junctional elements selected at different stages of B cell development that shape CDR H3 and junctional length distributions in antibody repertoires. That is, CDR H3 length distribution in the repertoire is not independent of heavy and light chain V_H_, V_L_, and V_H_/J_H_ germline segment usage as implicitly assumed^6–11,17–20^. Instead, overall CDR H3 and junctional length distributions in antibody repertoires are aggregates of several sub-repertoires with discrete sets of biases relative to each other that arise in different stages of B cell maturation as a function of V_H_, V_L_, and J_H_ germline segment use, shared by normal donors. Thus, proper description of CDR H3 length biases in disease and immune states^11,12,39^ requires the context of V_H_, V_L_, or V_H_/J_H_ germline segment usage in which these biases are observed for meaningful interpretation. The analyses described here provide a high-dimensional CDR H3 analytical framework, in which CDR H3 and junctional length distributions are analyzed in the context of V_H_, V_L_, and J_H_ germline segments and combinations of these, and a baseline of these biases with multiple healthy donors for further studies of B cell repertoire maturation and clonal selection in health and disease.

## Methods

### Datasets and analysis

Sequences were obtained from the original publications^14,21,25^ except for the MA dataset. The sequences in the MA dataset were obtained from a re-sequencing by Illumina MiSeq of a set of previously described samples^22^, deposited in the Sequence Read Archive (SRA) database^24^. A summary of the samples used here is given in Supplementary Data 5. Sequencing methods for the MA dataset are described in the experiment design section associated with sample data (see https://www.ncbi.nlm.nih.gov/sra/SRX2251687). The SRI dataset donors included in the analyses are 316188, 326650, 326737, 326780, 326797, 326907, 327059, and D103^20^. SRI dataset donors 326713 and 326651 were only used for V_H_ germline segment allele-specific analyses. Sequences were used as parsed in the original publications except for sequences of the MA dataset obtained from the SRA database^24^, where the raw sequencing files were processed and germline segments annotated with a custom pipeline (available from docker hub repository at https://hub.docker.com/r/kamhonhoi/iganalysis). Briefly, paired-end reads were merged using FLASH^40^ to reconstruct the full-length variable domain sequences using the following parameters: read length at 300 bps, expected fragment length at 530 bps, standard deviation at 50 bps. The full-length sequences were subsequently processed to identify the frameworks and CDR regions using position-weighted motifs as previously described^41^. IgBlast^42^ was used to supplement the region parsed data with germline segment annotation for each sequence, including nucleotide somatic mutations. Isotypes of the sequences were determined by finding the closest matching human CH1 isotypes on the available CH1 sequences. Each sequence was processed and annotated with the frameworks, CDRs, germline segment use and clonotype grouping (see below). Nonproductive sequences in the WA dataset used for analyses were limited to frameshifted sequences in the naive compartment to minimize the indirect effects of sequencing errors and clonal expansion. CDR H3 length of nonproductive, frameshifted sequence length in amino acid residues was set as the nearest integer of CDR H3 length in nucleotides divided by 3. For naive compartment sequences of Donor 1 of the WA dataset only the D1a repeat was used for most analyses^25^. All CDR H3 length distributions and germline segment prevalence analyses were determined using custom scripts and Microsoft Excel 2016. The IMGT® CDR definition and numbering system are used throughout^26^.

### Clonotype clustering

Clonotypes in the CA and TX datasets were defined as sequences from the same donor, V_H_ and V_L_ germline segments and CDR H3 length with a nominal 57% or greater CDR H3 amino acid sequence identity, which better approximates an average 60% CDR H3 sequence identity across the range of CDR H3 lengths. IgG/IgA and IgM sequences were segregated prior to clonotype clustering. For the TX dataset IgG/IgA, CD27^pos^/IgM and CD27^neg^ sequences were segregated prior to clonotype clustering. The 11% of sequences without isotype information in the TX AE datasets, which included IgG, IgA, and IgM sequences, were excluded from the analyses due to the differences between IgG/IgA and IgM overall distributions in the AE compartment (Fig. 1b). Clonotypes in the MA dataset were defined as sequences from the same donor, V_H_ and J_H_ germline segments and CDR H3 length with a nominal 57% (average 60% identity across CDR H3 lengths) or greater CDR H3 amino acid sequence identity as above. Clonotypes in the SRI dataset were defined as sequences from the same donor with the same V_H_ and J_H_ germline segment, isotype and same CDR H3 length and sequence. Only sequences labeled as “productive” in the SRI dataset were analyzed. Only a randomly chosen sequence from each clonotype was retained in the dataset for the TX, CA, WA, MA, and SRI datasets. Clonotypes in the WA dataset were defined as sequences from the same donor with the same V_H_ and J_H_ germline segment and same CDR H3 length and sequence. If V_H_ germline segment information was not available then V_H_ subfamily information was used in lieu, retaining as a representative for the clonotype a sequence with V_H_ germline segment information if available. If J_H_ germline segment information was not available then this parameter was ignored, also retaining otherwise identical sequences with available J_H_ information as representatives for clonotypes, if available. Nonproductive sequences in the WA dataset were not processed for clonotype clustering.

### Repertoire similarity index analysis

RSI was computed in a manner similar to a previously described method^27^. For a given set ***S*** of CDR H3 sequences, all of the same length *n*, RSI is measured as follows:

$$RSI = \left[ {1 - \frac{{{\mathrm{median}}\left\{ {LD\left( {S_i,S_j} \right)_{i \ne j}} \right\}}}{n}} \right] \times 100\% \,\forall \,1 \le i,j \le |{\boldsymbol{S}}|$$

where *S*_*j*_ and *S*_*j*_ refer to any two sequences in the set of CDR H3 sequences and *LD*(*S*_*i*_,*S*_*j*_) refers to the Levenshtein distance function, which measures the number of amino acid changes necessary to convert *S*_*i*_ to *S*_*j*_. For a given V_H_ germline segment and CDR H3 length, RSI values were computed for those sequences that shared the same V_L_ germline segment (for the paired CA and TX datasets) or the same J_H_ germline segment (for the unpaired MA datasets) and the same CDR H3 length. Values were computed separately for each donor in the datasets and averaged for each length. Values shown in graphs in Supplementary Fig. 5 are the averages in each length for different datasets. All calculations were performed using custom scripts in R.

### Principal component analysis of CDR H3 length distributions

The length distribution of each germline segment was captured as a vector of length 22 containing the percentage of sequences of length 5–26. For V_H_, the values for each germline segment were averaged overall the AE datasets except the WA dataset due to limited germline segment coverage. For V_L_, the values were averaged over the CA and TX datasets. The distributions of each germline segment were consolidated into a matrix ***X***_*n*×22_ where *n* is the number of germline segments considered for analysis (*n* = 39 for V_H_ and *n* = 35 for V_L_). The variance covariance matrix ***S***_**22×22**_ of X was computed with elements *S*_*ij*_ as

$$S_{ij} = < \left( {X - \left\langle {X_i} \right\rangle } \right)\left( {X - \left\langle {X_j} \right\rangle > \forall \,1 \le i,j \le 22} \right.$$

where <> refers to average across all germline segments. Eigen decomposition of the matrix ***S*** results in 22 eigenvectors, each of which capture a trend in the distribution as a function of the CDR H3 lengths and are sorted in decreasing order of the variance they capture. Each germline segment was then projected onto these eigenvectors to obtain the PC scores which enabled visualization of the different trends and comparisons among the different germline segments. Principal Component Analysis was performed using the ‘princomp’ function and Repertoire Similarity Index, implemented using the ‘sdists’ function in the package ‘cba’ in R version 3.5.0.

### Statistics and reproducibility

Samples consist of independently sequenced antibody repertoires from 20 donors from five laboratories under different sequencing conditions, comprising five datasets of with 2–8 donors each with varying number of sequences per donor (see Supplementary Data 1, 2, and 3 for details and sample sizes for each dataset, donor, B cell subset and germline segment). To avoid over-representation of sequences from donors and datasets with higher sequence counts, calculations were performed per donor and then averaged per dataset or across datasets except for Supplementary Fig. 1, where sequences from donors of each dataset were pooled prior to calculations. Germline segments within a donor with fewer than 80 counts were excluded from analyses. Reproducibility was assessed by comparing distributions between datasets, with the exception for nonproductive sequences, which were present only in the WA dataset. Two-tailed paired *t*-tests of CDR H3 length distributions were performed using Microsoft Excel for the Mac 2016. Data from individual donors comparing germline segment-specific to overall repertoire CDR H3 length prevalence from the CA, TX, MA, and WA datasets (*n* = 11 donors for V_H_, *n* = 5 donors for V_L_) were used for *t-*tests of AE compartment samples over a sliding window of two consecutive CDR H3 lengths to minimize local fluctuations. *P*-value thresholds of 10^−4^ (V_H_) and 10^−2^ (V_L_) were used to assess significance across the range of CDR H3 lengths. Mann-Whitney tests for distributions in Supplementary Fig. 17 were done using GraphPad Prism version 6.

### Reporting summary

Further information on research design is available in the Nature Research Reporting Summary linked to this article.

## Supplementary information

Supplementary Information

Supplementary Data 1-5

Supplementary Data 6

Reporting Summary

Description of Additional Supplementary Files

## Data Availability

Sequence datasets used in analyses have been previously described^20–25^. The subset of the datasets with sequences clustered by clonotype used here are available at https://doi.org/10.5061/dryad.cjsxksn2x ^43^. All data supporting this study are available within the article and its Supplementary Information Figures and Supplementary Tables or are available from the corresponding author on reasonable request.

## Code availability

Raw NGS sequencing data analyzed with a custom analysis pipeline, available from the docker hub repository at hub.docker.com under kamhonhoi/iganalysis. Principal Component Analysis performed using the ‘princomp’ function and Repertoire Similarity Index, implemented using the ‘sdists’ function in the package ‘cba’ in R version 3.5.0.
