# Supplementary material for: Dynamics of heavy chain junctional length biases in antibody repertoires
Source: Commun Biol. 2020 May 1;3:207. doi: 10.1038/s42003-020-0931-3 (PMC7195405; doi:10.1038/s42003-020-0931-3)
Supplement: Supplementary file 1 — Supplementary Information [file 42003_2020_931_MOESM1_ESM.pdf]

Dynamics of heavy chain junctional length biases in antibody repertoires

Supplementary Information

Supplementary Figures

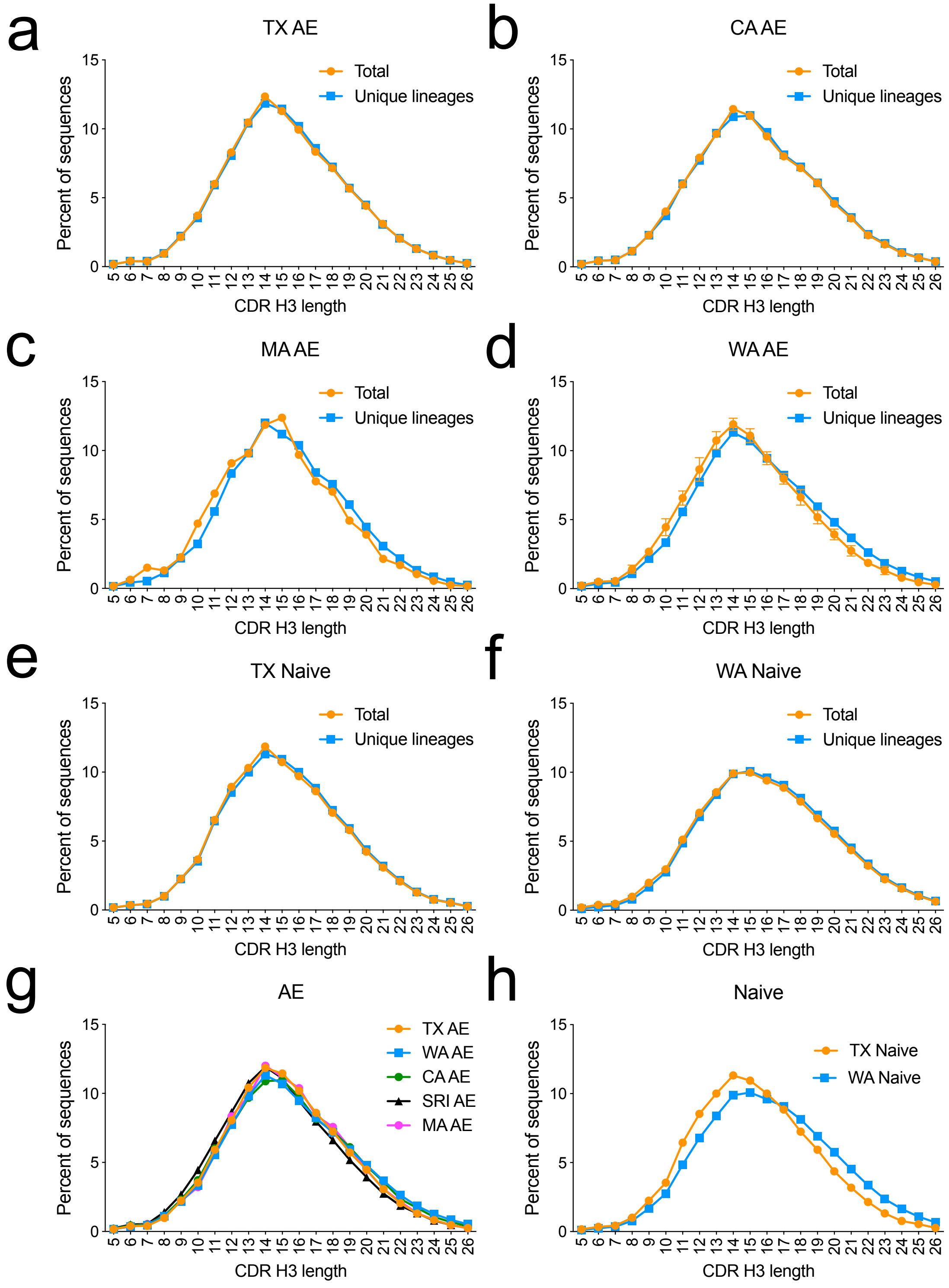

Supplementary Figure 1. (a-f) CDR H3 length distribution of raw, as published, data and unique lineages (clonotypes) in the AE and naive B cell compartments. (g-h) Comparative CDR H3 length distributions of unique lineages in the AE and naive B cell compartments.

Supplementary Figure 2

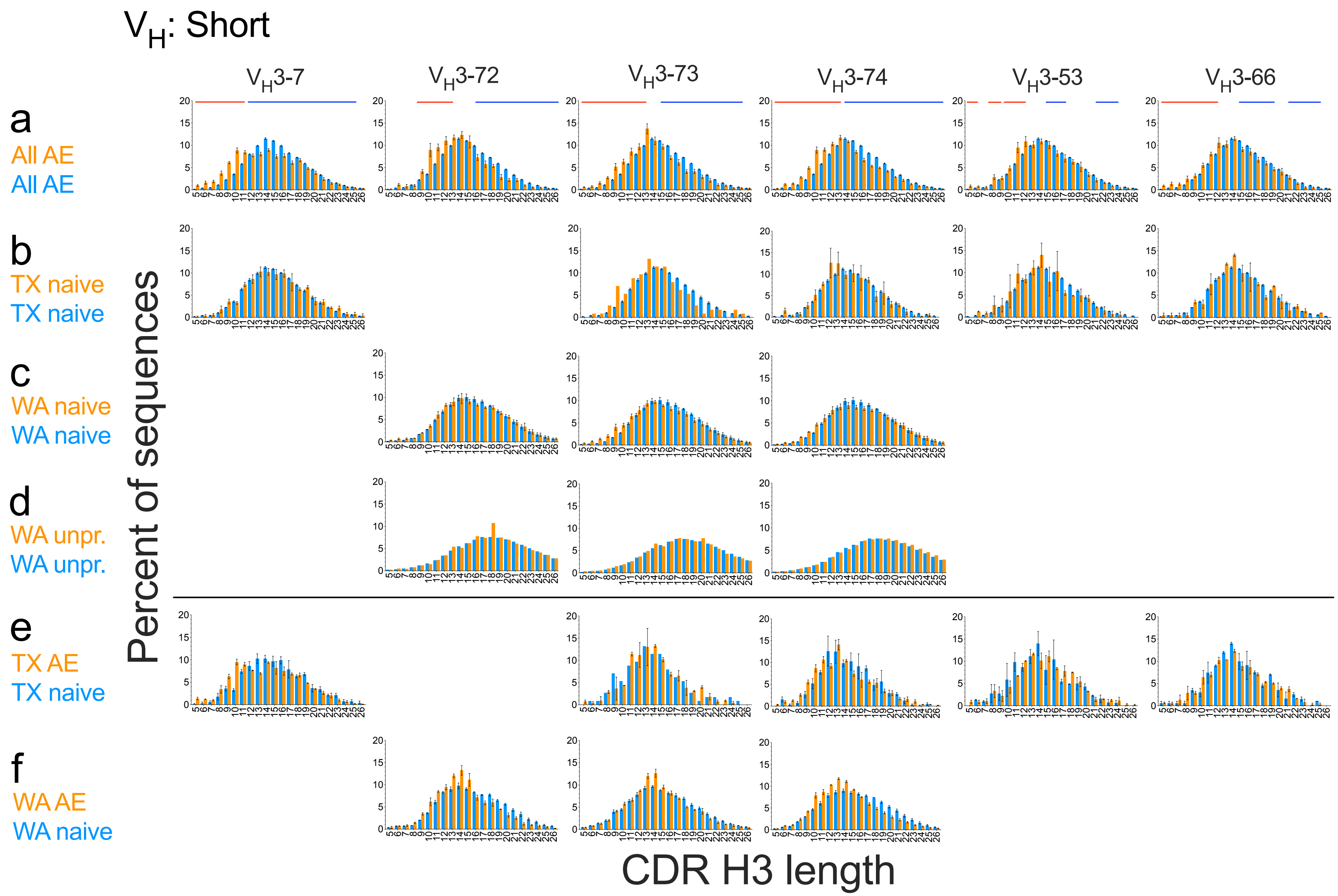

Supplementary Figure 2

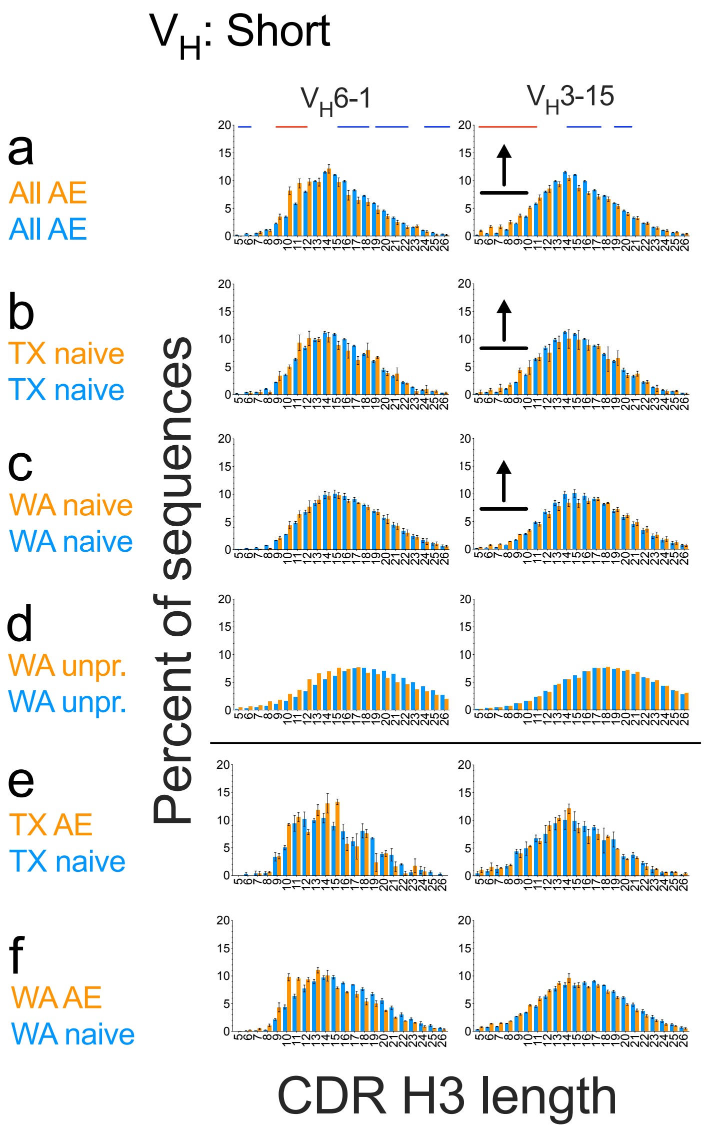

Supplementary Figure 2

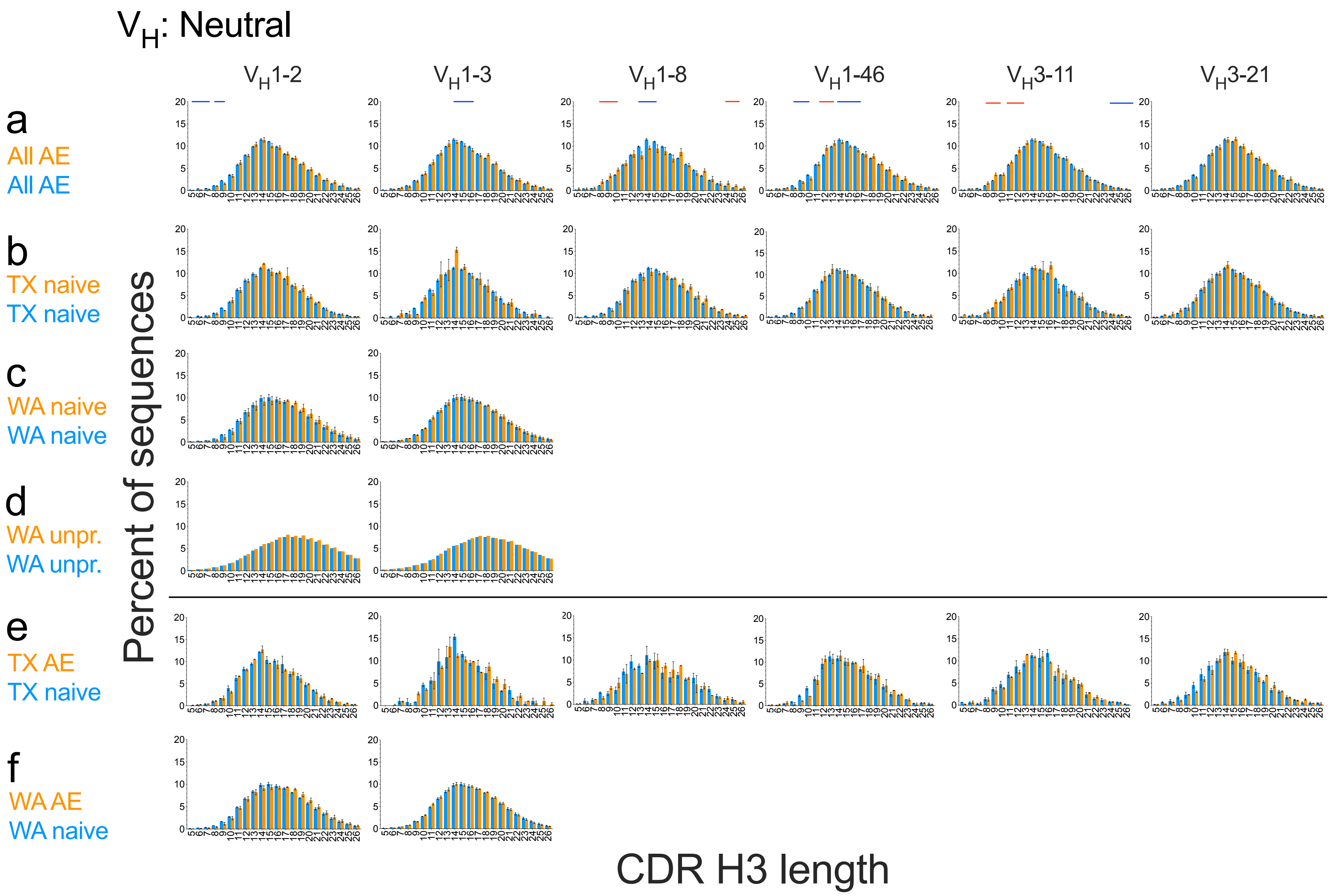

Supplementary Figure 2

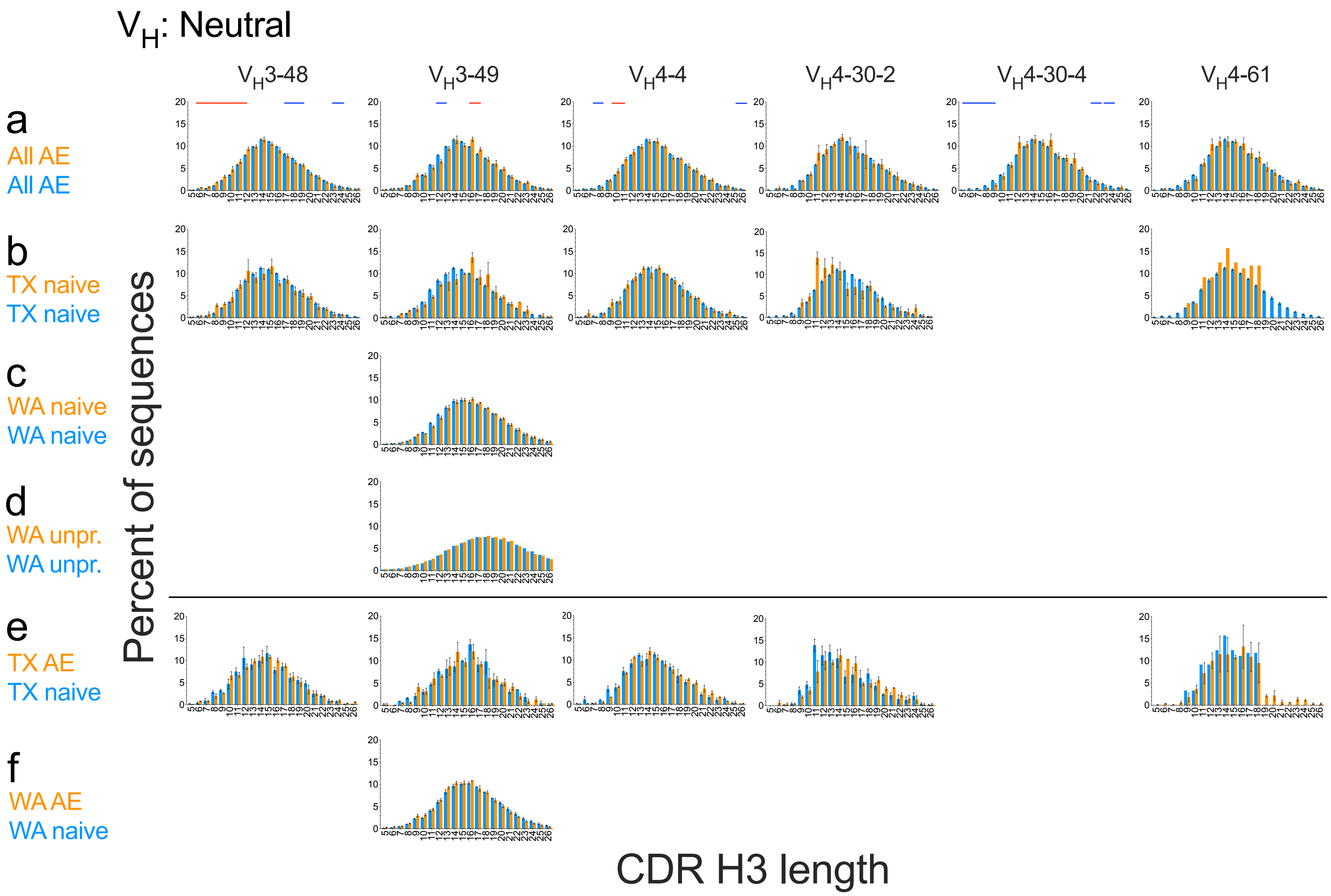

Supplementary Figure 2

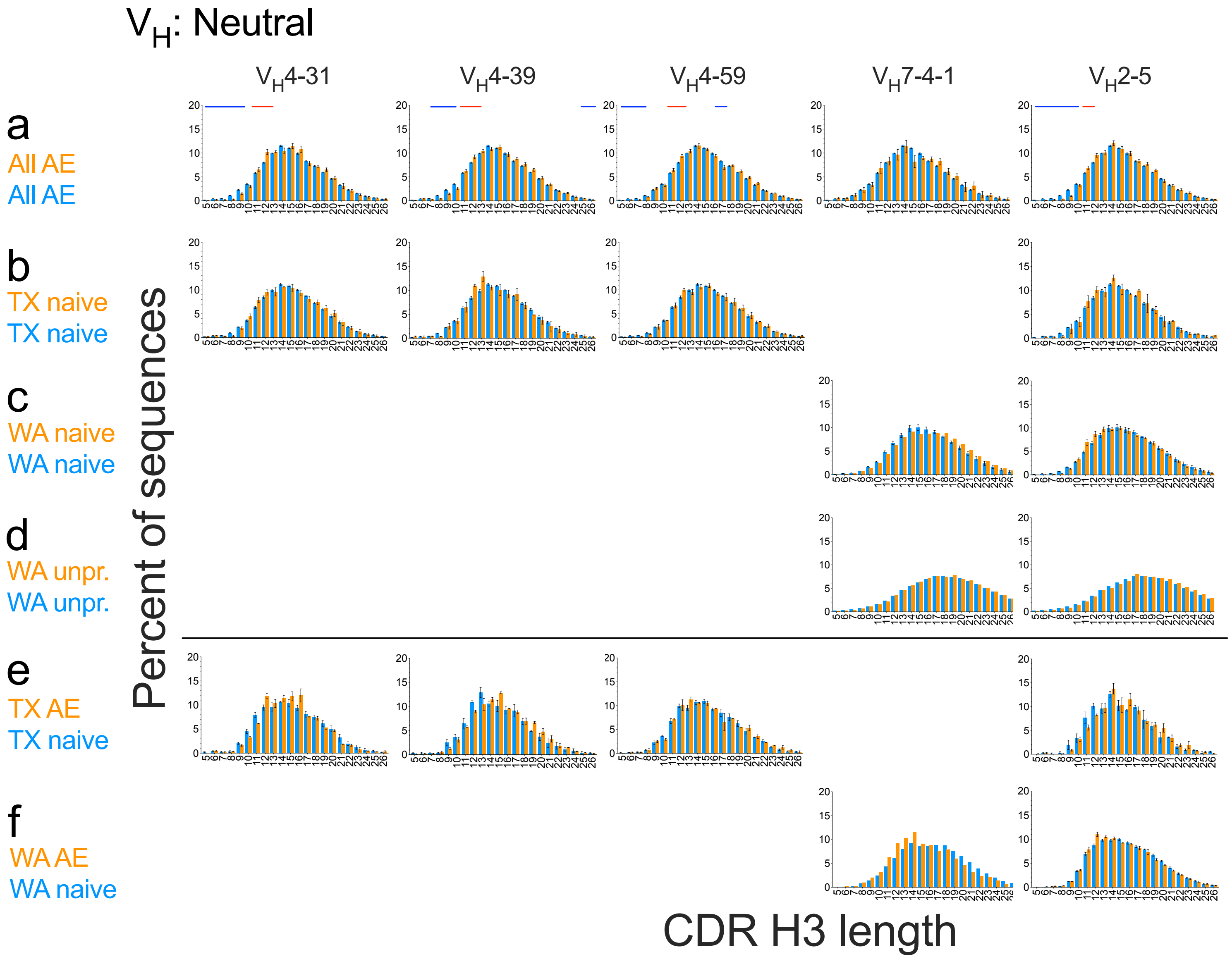

Supplementary Figure 2

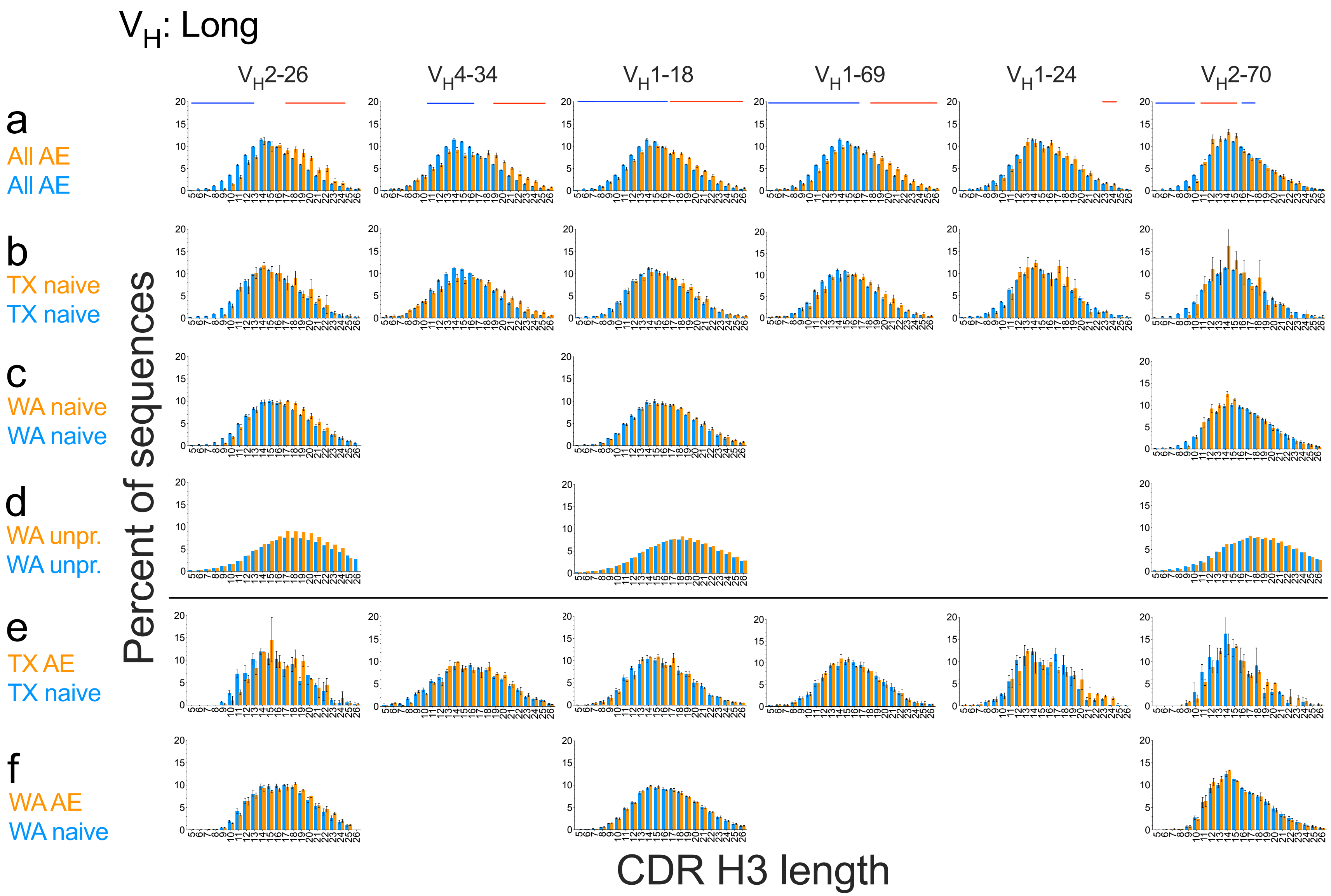

Supplementary Figure 2

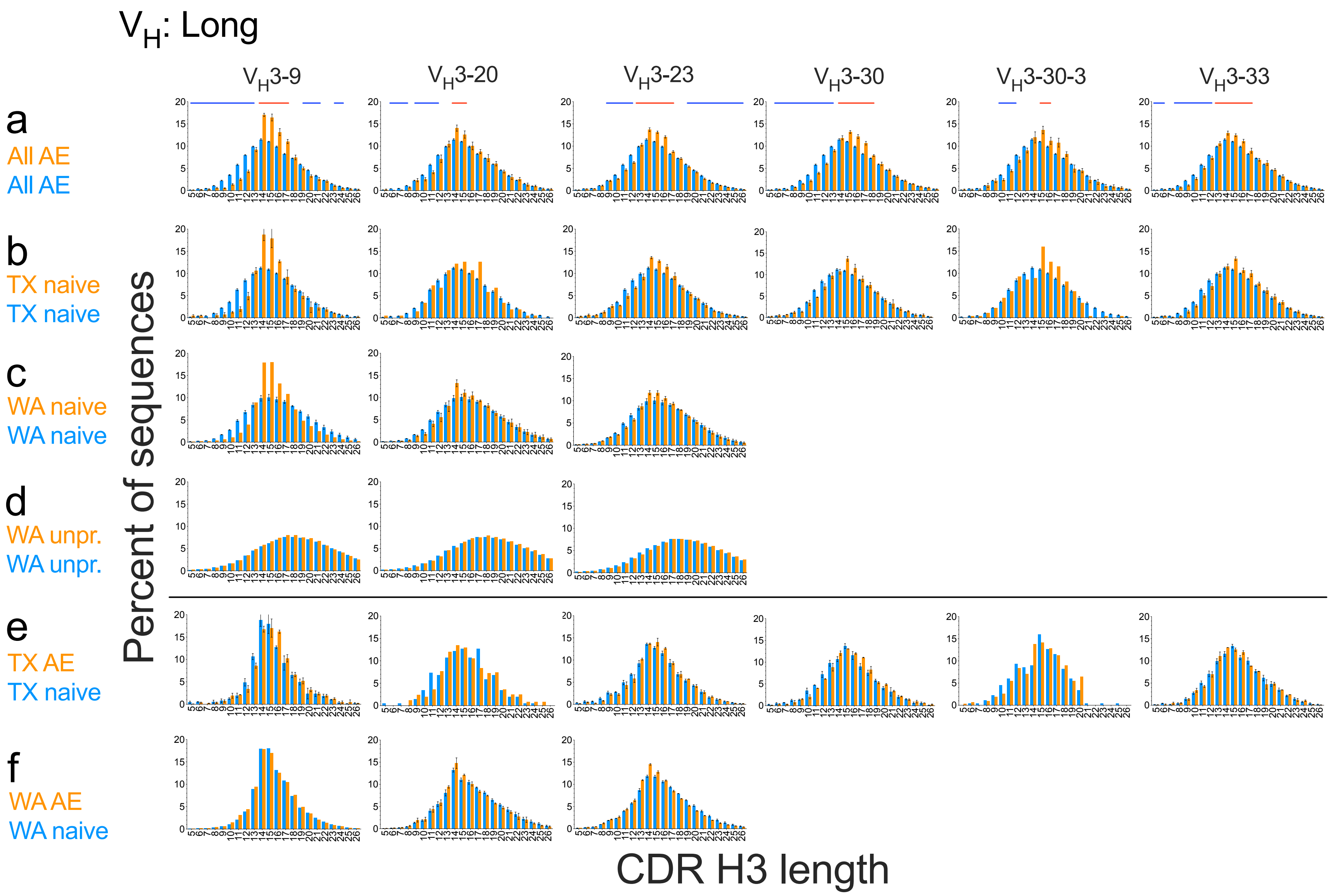

Supplementary Figure 2

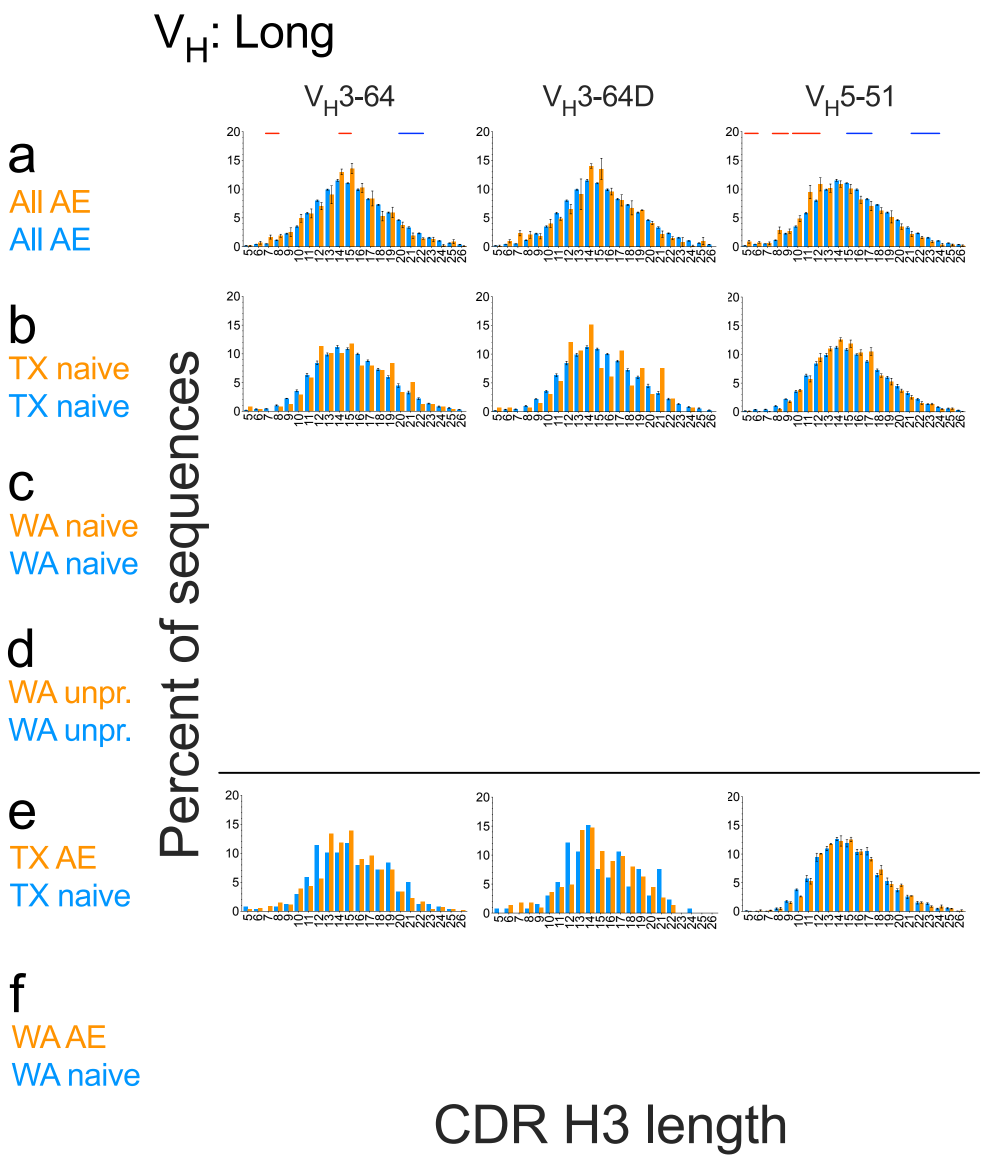

Supplementary Figure 2

Supplementary Figure 2. CDR H3 length distributions associated with V_H germline segments (orange bars) compared to overall distribution in the same samples and compartments (rows a-d, blue bars) or to corresponding naive sequences (rows e and f):

Row (a): AE sequences, all CA, TX, MA and WA donors pooled (n = 11)

Row (b) TX naive compartment (n = 3)

Row (c) WA naive compartment (n = 3)

Row (d) WA V_H unproductive sequences (n = 3)

Row (e) TX AE (orange bars, n = 2, same as orange bars in Suppl. Figure 3, TX) and naive sequences (blue bars, n = 3, same as orange bars in row b)

Row (f) WA AE (orange bars, same as orange bars in Suppl. Figure 3, WA) and naive sequences (blue bars, same as orange bars in row c) (n = 3)

Red and blue horizontal bars above the histograms in (a) indicate statistically significant ($P < 10^{-4}$) differences between the germline segment-specific and overall distributions in a paired (within donors, n = 11 donors) *t*-test. The “all” label in row (a) refers to datasets CA, TX, MA, and WA. Error bars indicate S.E.M. except orange bars in panel e, orange bars (n = 2 donors in TX AE), which indicate range. Arrows indicate subtle enrichment of sequences in the distributions. Figure tags refer to each row of graphs.

Supplementary Figure 3

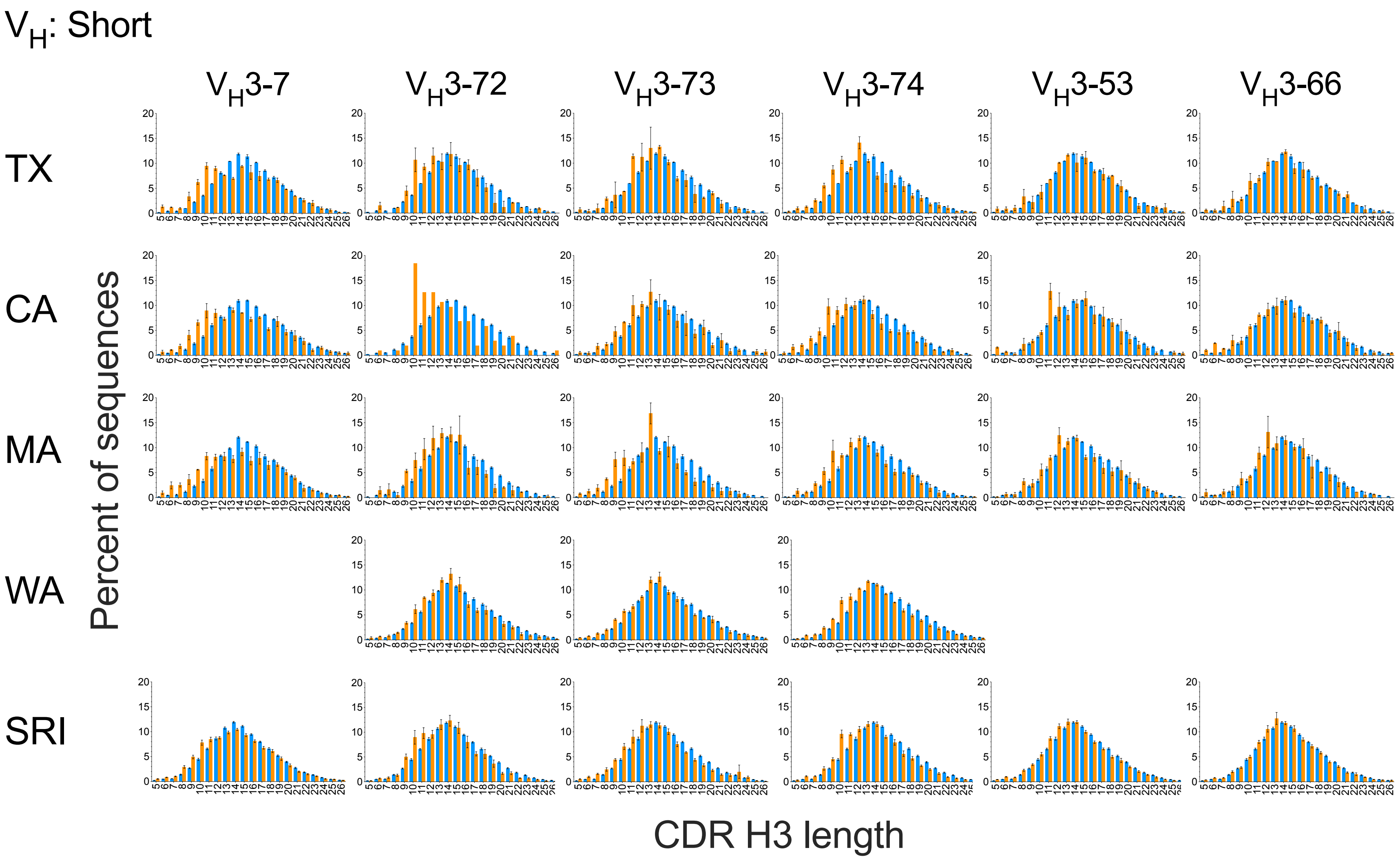

Supplementary Figure 3

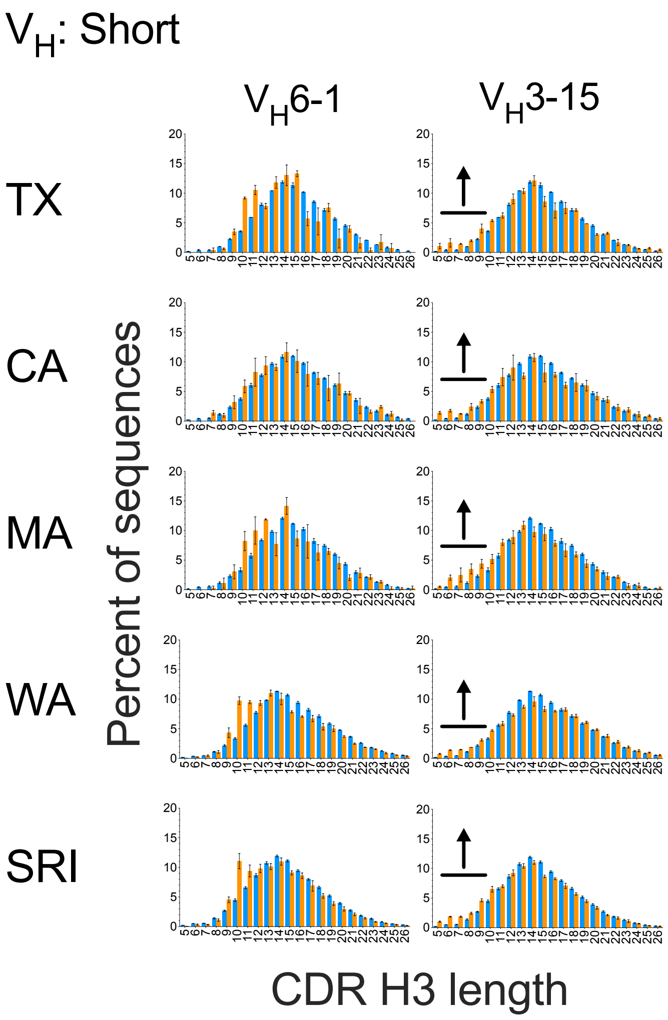

Supplementary Figure 3

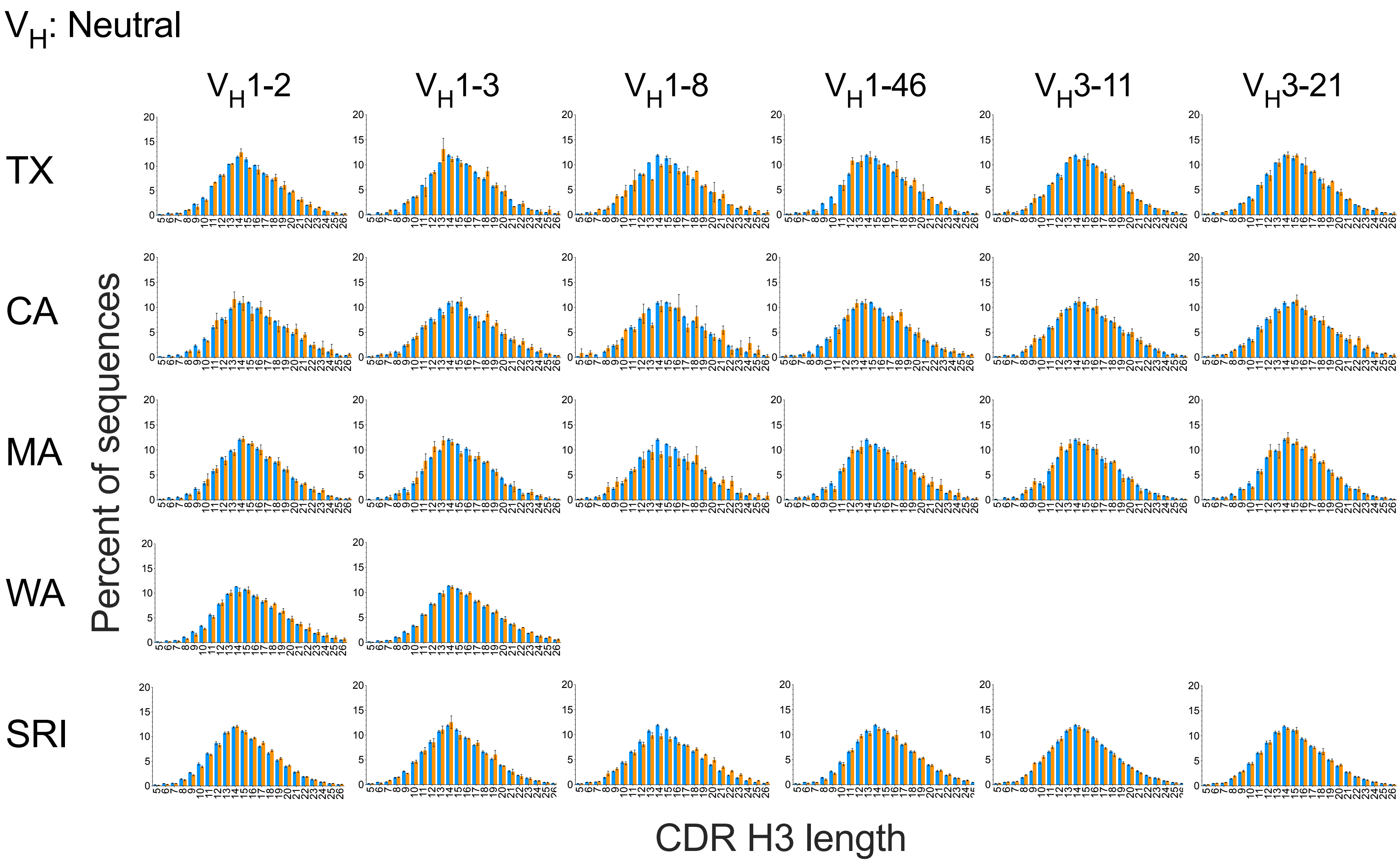

Supplementary Figure 3

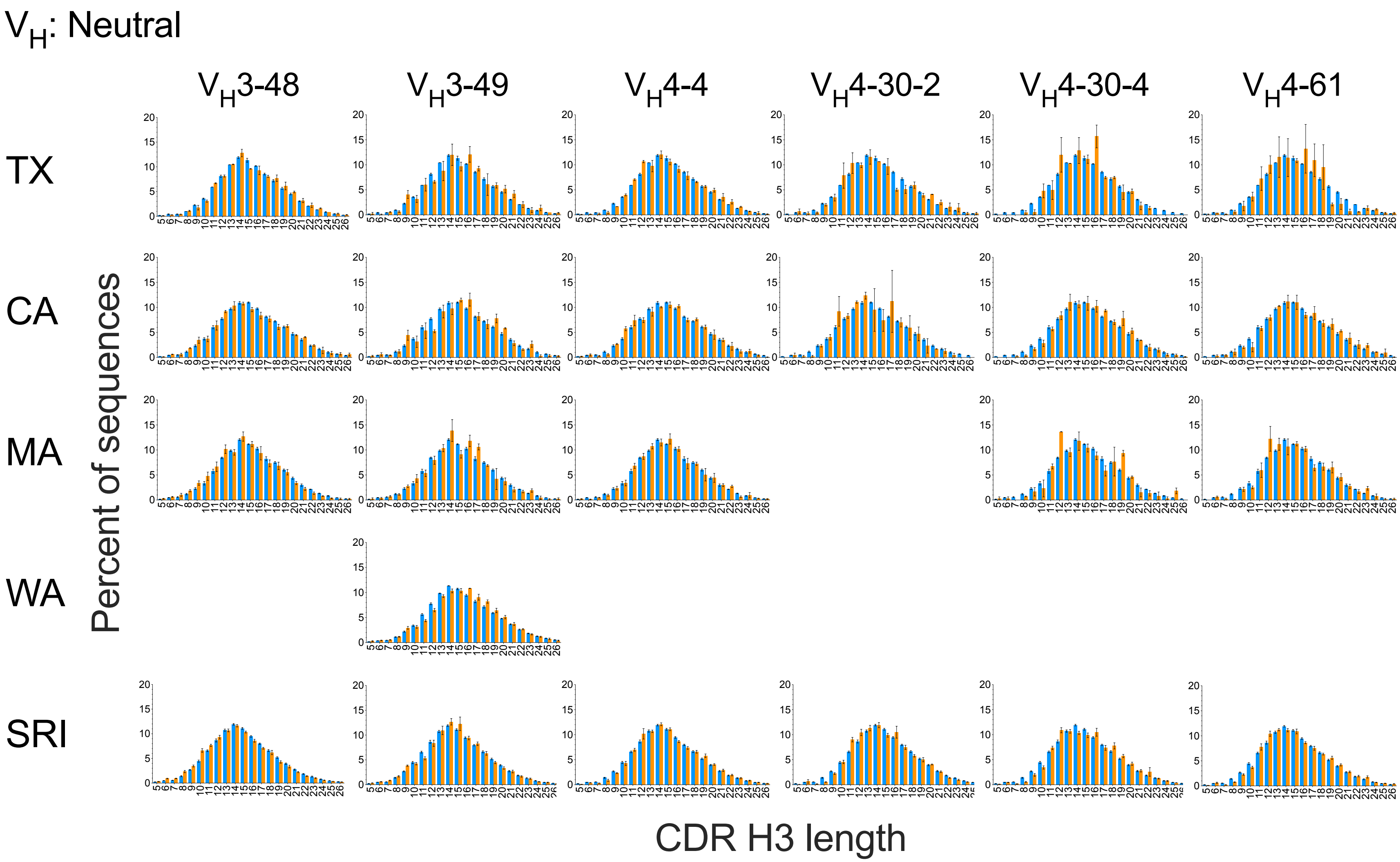

Supplementary Figure 3

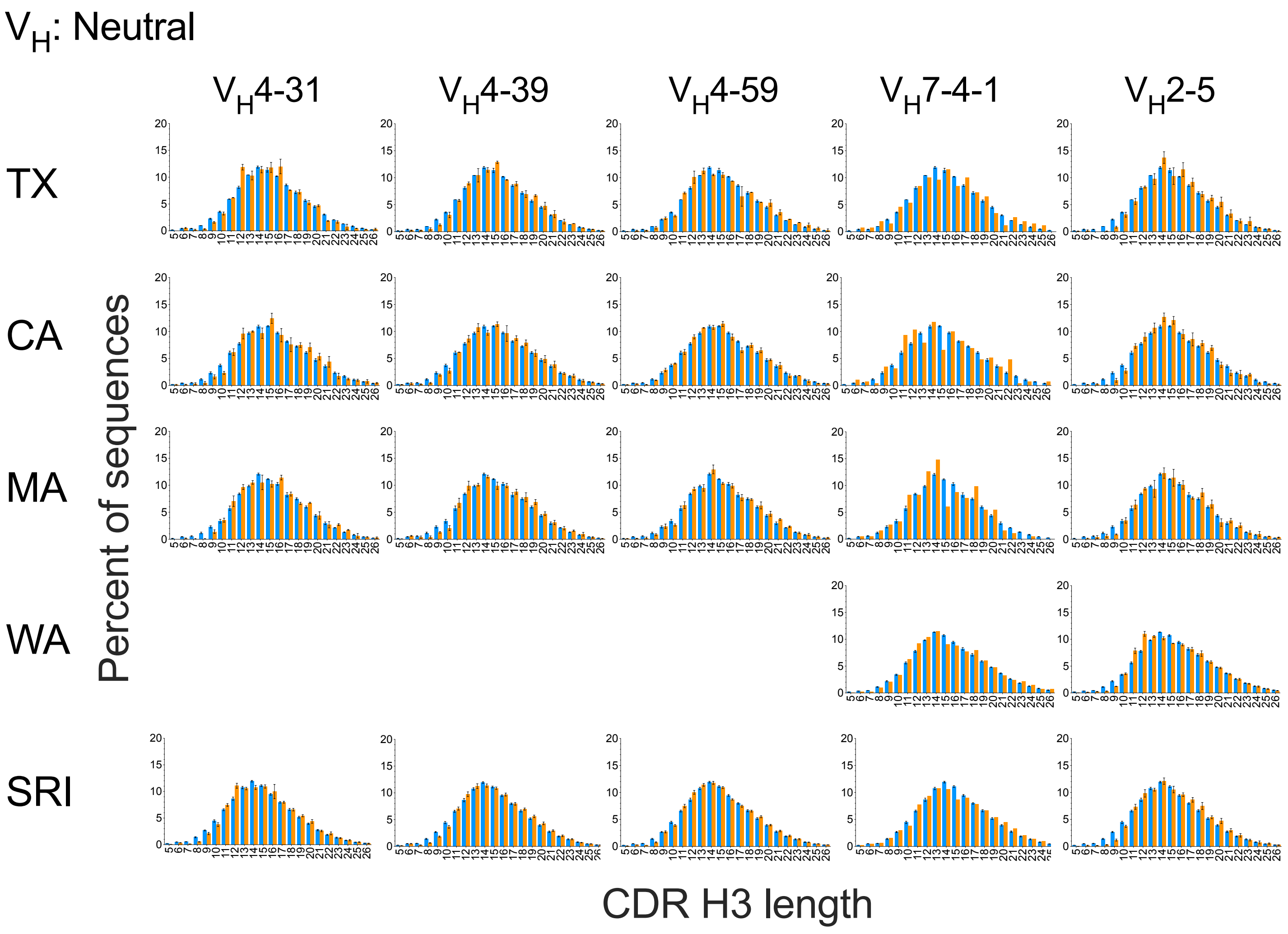

Supplementary Figure 3

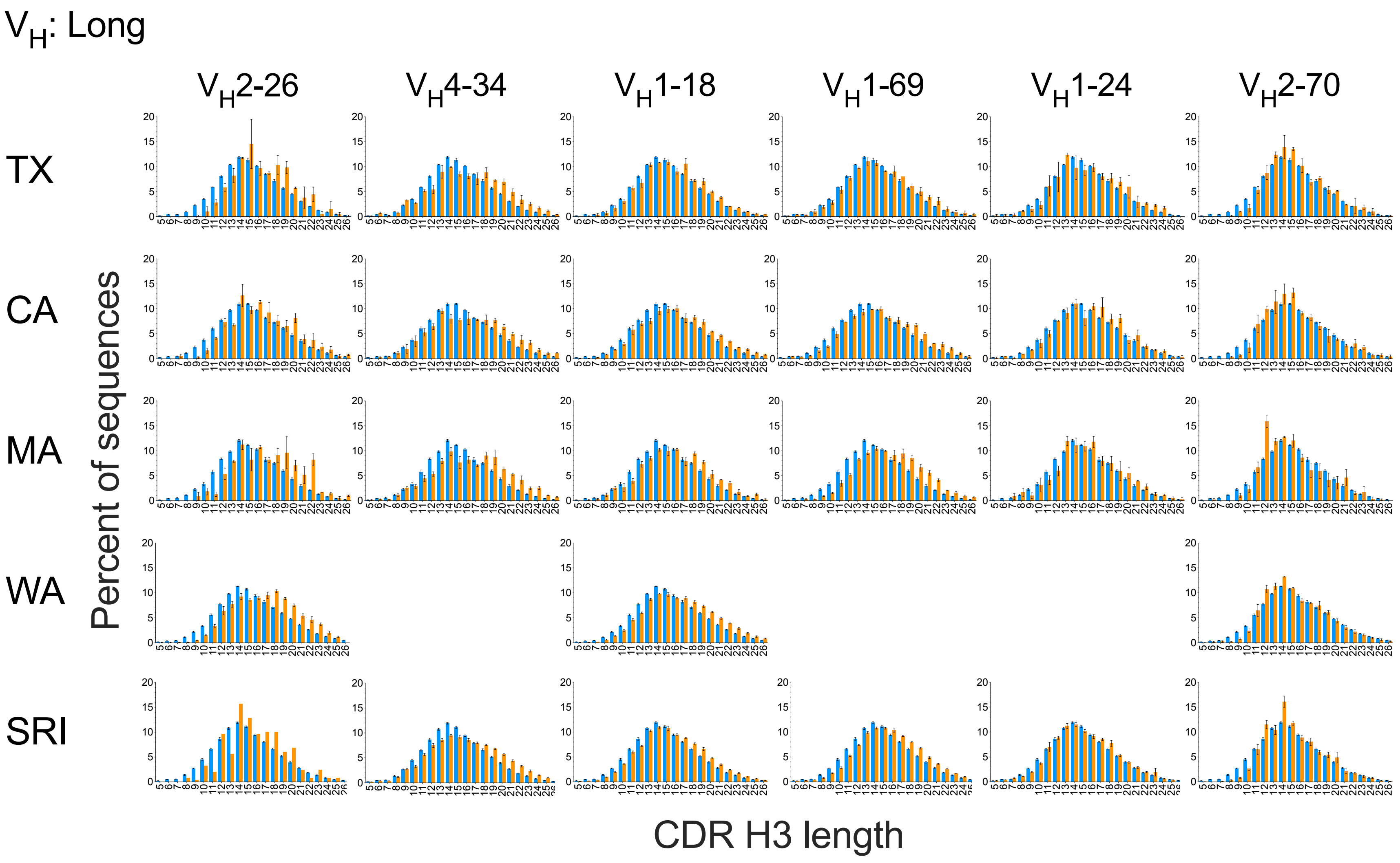

Supplementary Figure 3

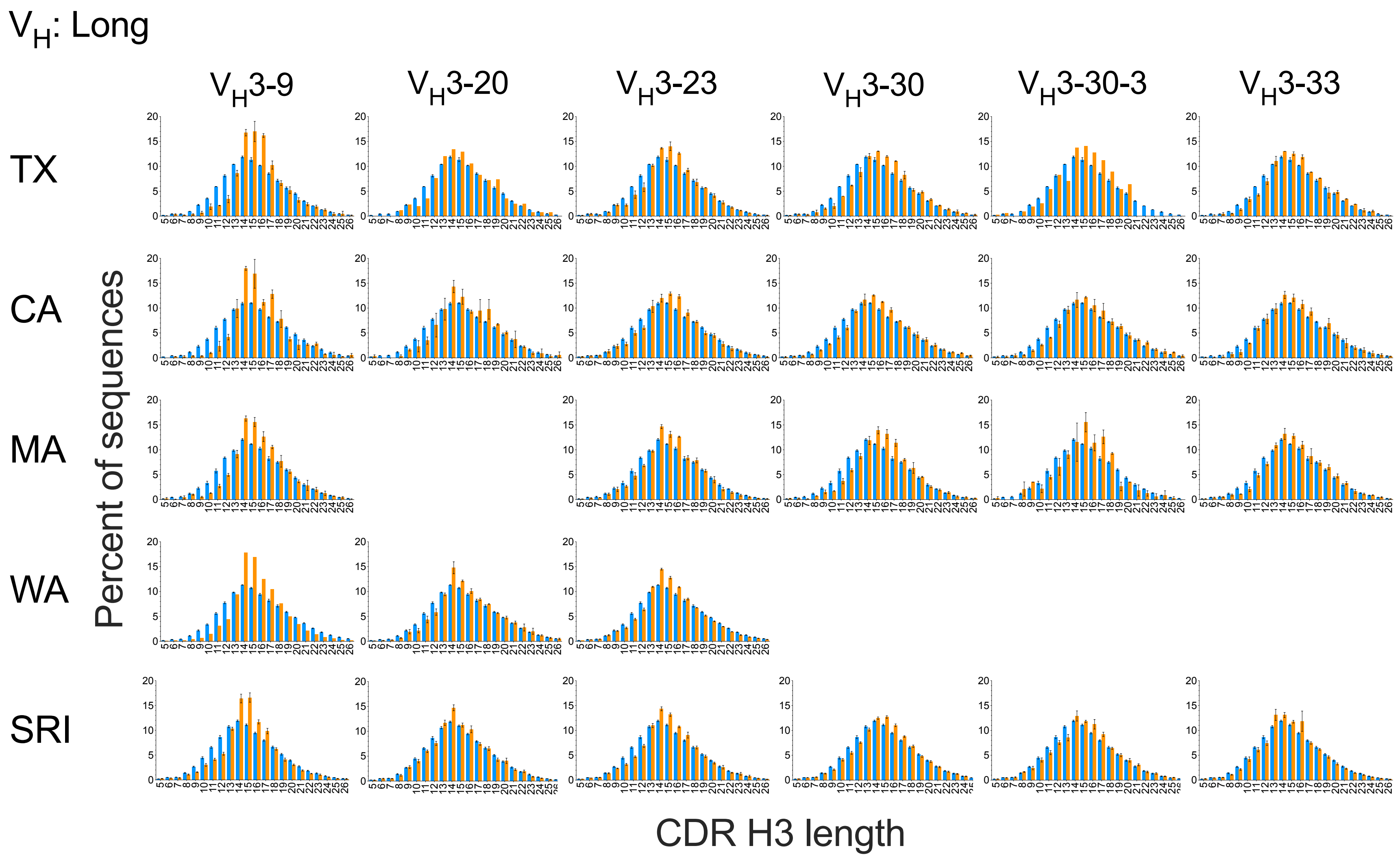

Supplementary Figure 3

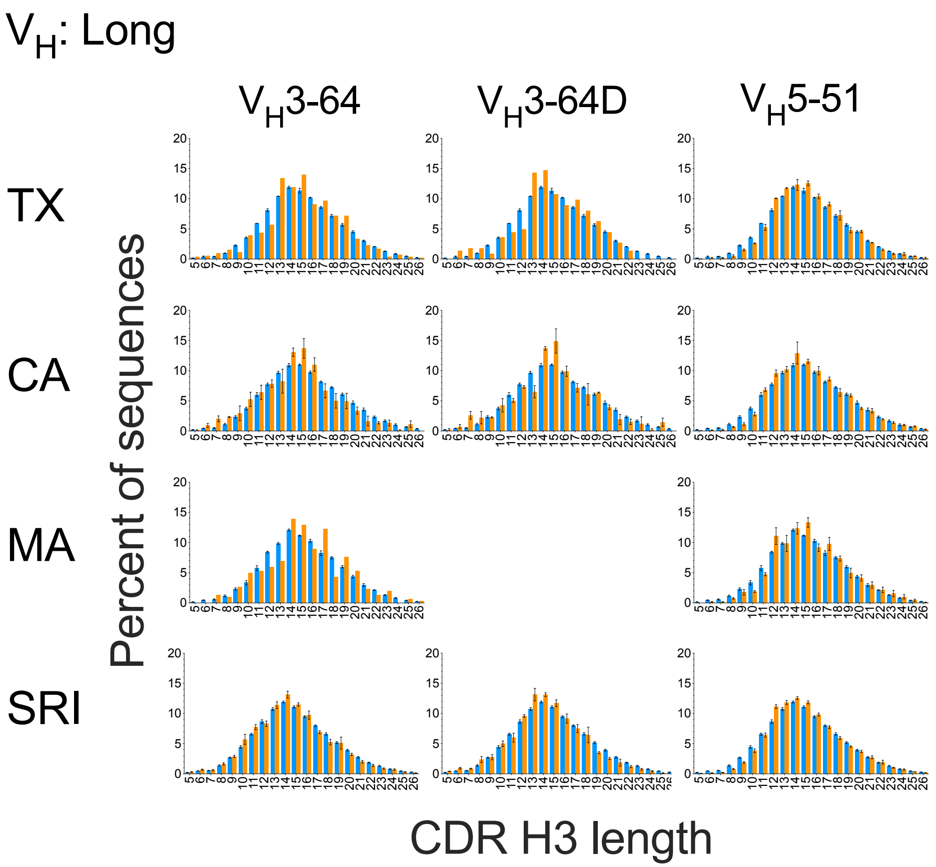

Supplementary Figure 3. Comparability of V_H-associated CDR H3 length biases among AE datasets. V_H germline segment-specific CDR H3 length distributions (orange bars) compared to overall distribution (blue bars) in the AE B cell compartments of the five datasets. Error bars indicate S.E.M. except for TX (n = 2 for TX AE donors), which indicates range. Arrows indicate subtle enrichment of sequences in the distributions shared among datasets.

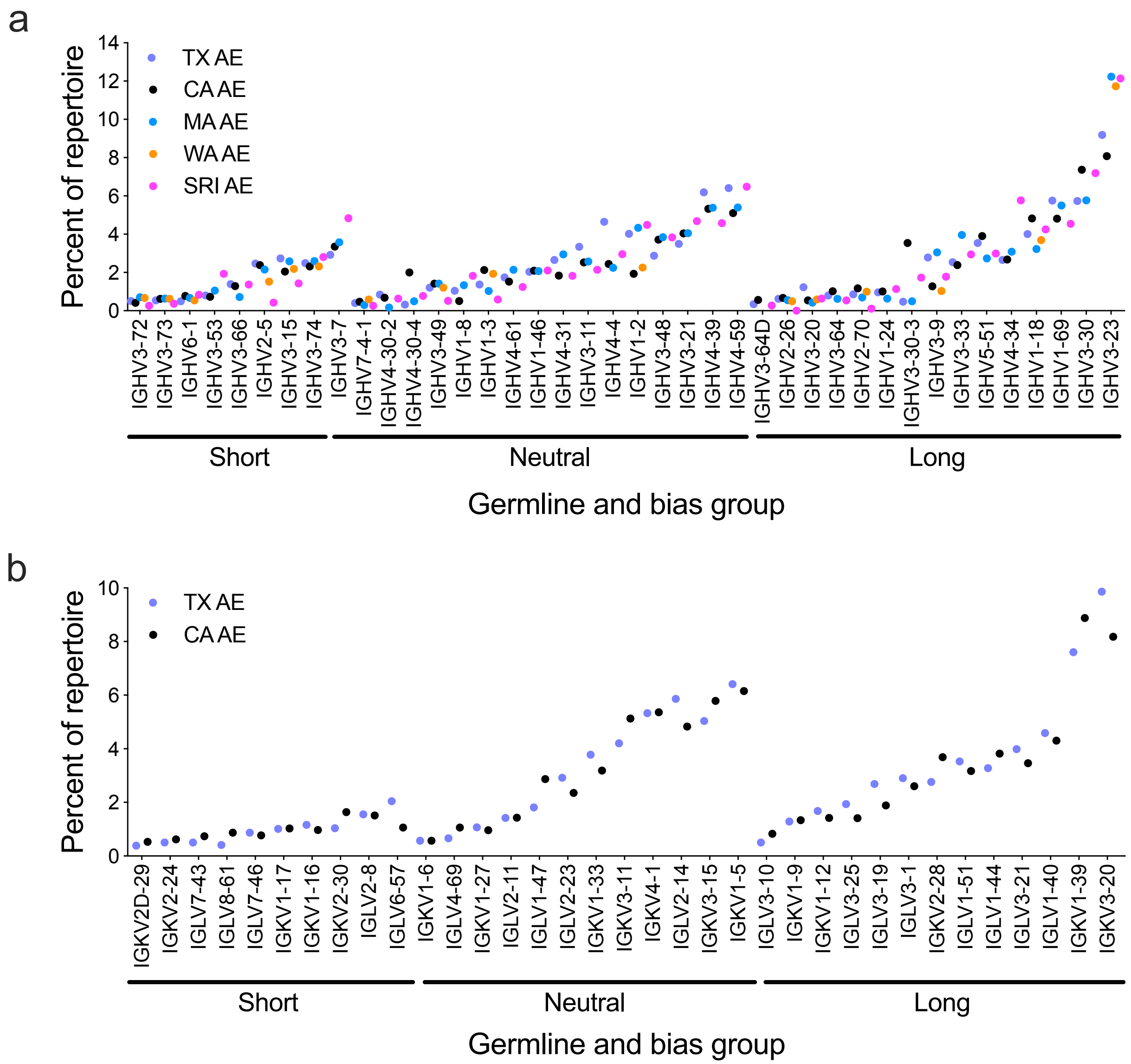

Supplementary Figure 4. Prevalence of V_H (a) and V_L (b) germline segments in the AE compartment. Prevalence of germline segments was calculated based on unique clonotypes after pooling of donors within each dataset.

Supplementary Figure 5

a

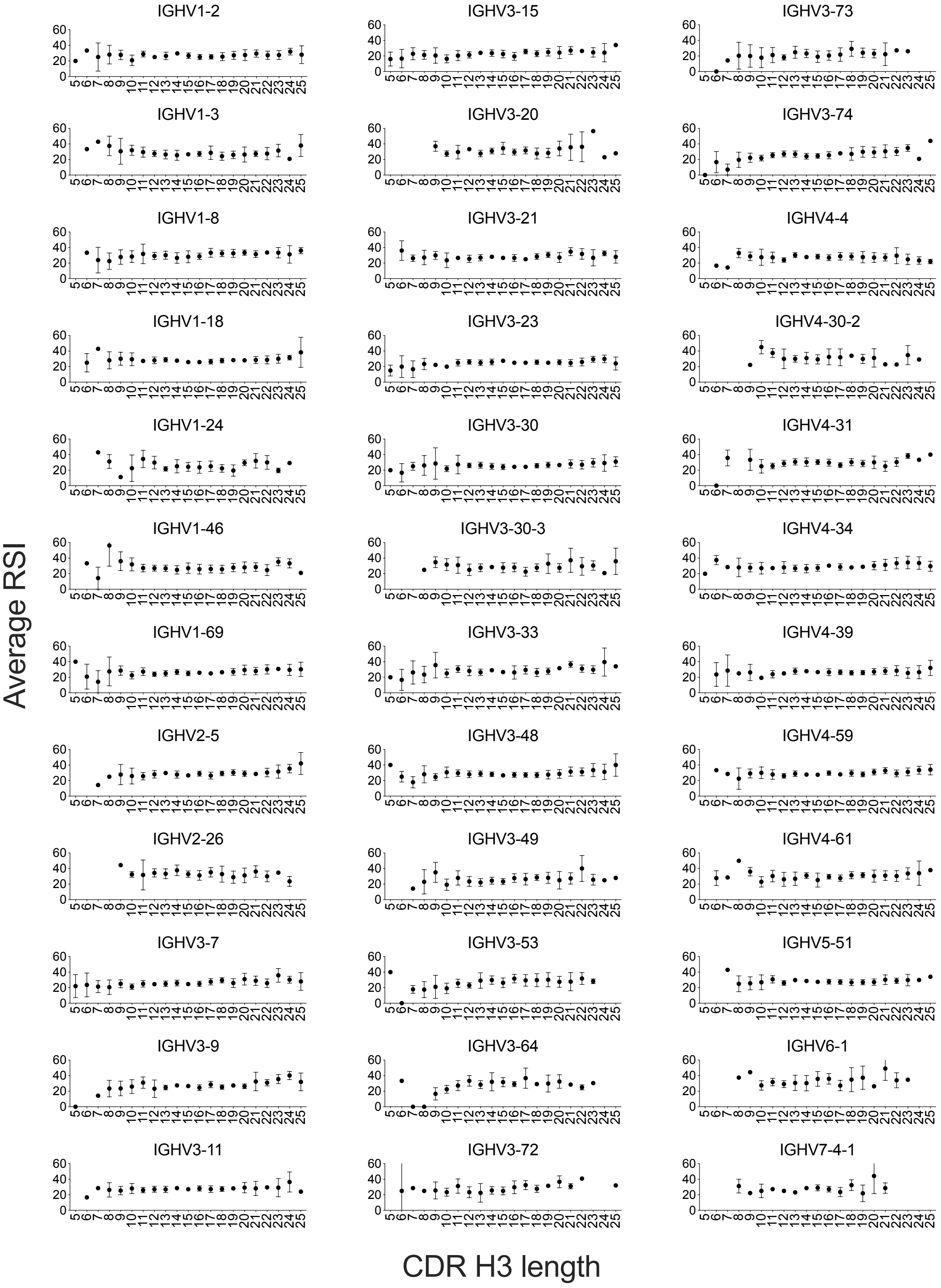

Supplementary Figure 5

b

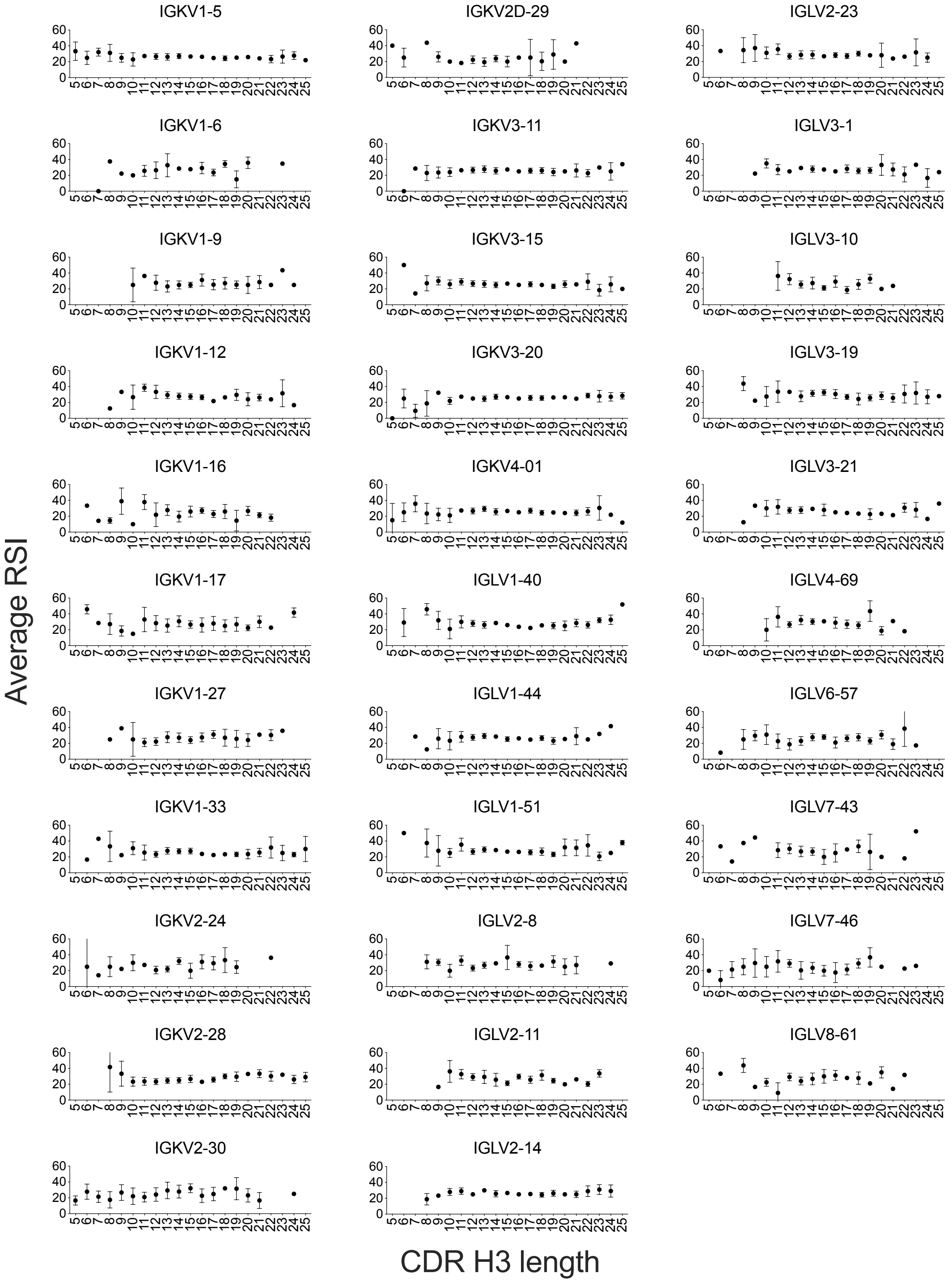

Supplementary Figure 5. RSI analysis of V_H (a) and V_L (b) germline segment-associated sequences. Points show average and standard deviation of the RSI value for sequences from each donor, germline segment and CDR H3 length. WA and SRI sequences were excluded due to large dataset sizes. Note that RSI values cannot be higher than 60 in the TX, CA and MA datasets due to clonotype definition. Germline segments are noted in the IMGT naming convention.

Supplementary Figure 6

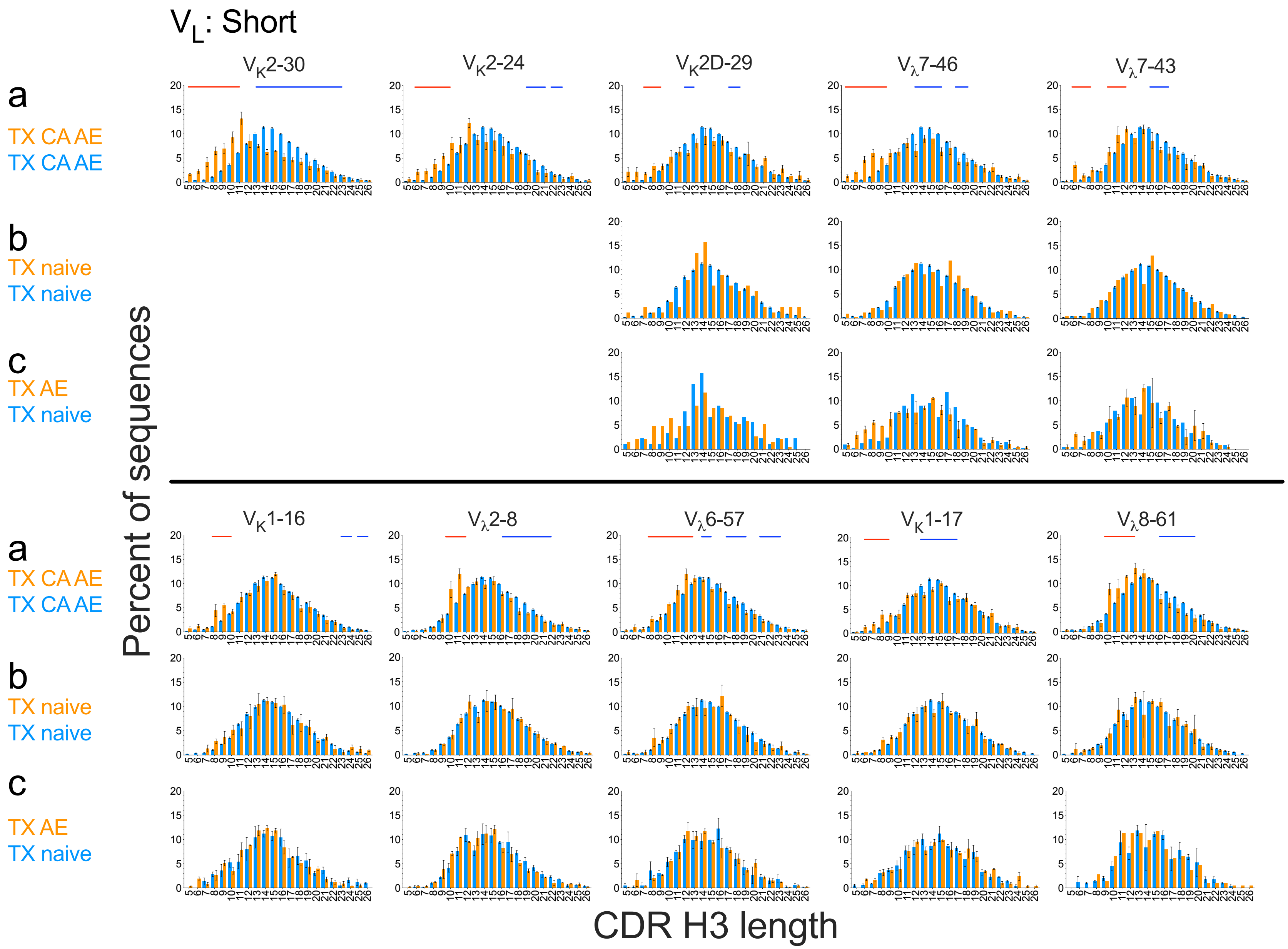

Supplementary Figure 6

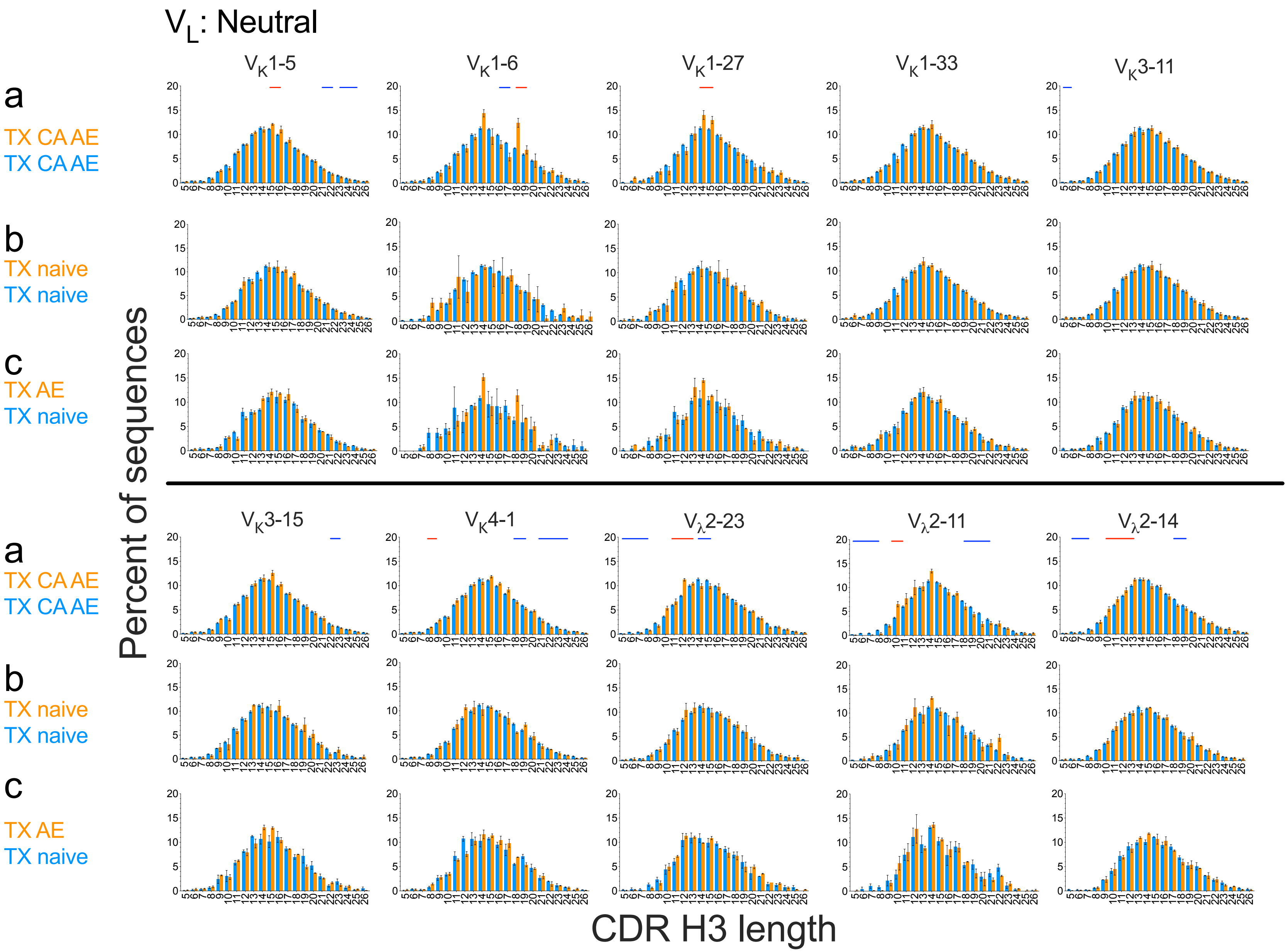

Supplementary Figure 6

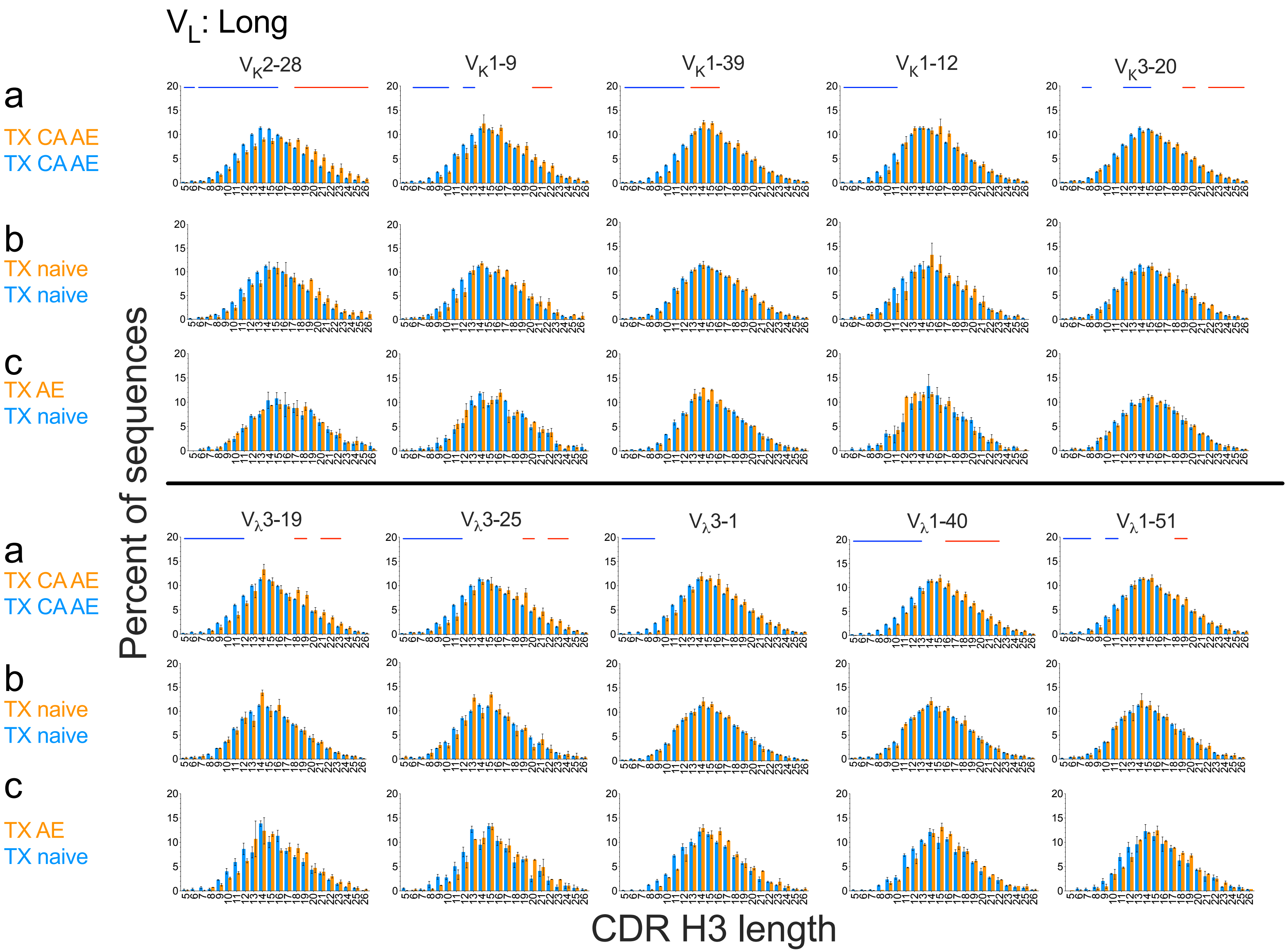

Supplementary Figure 6

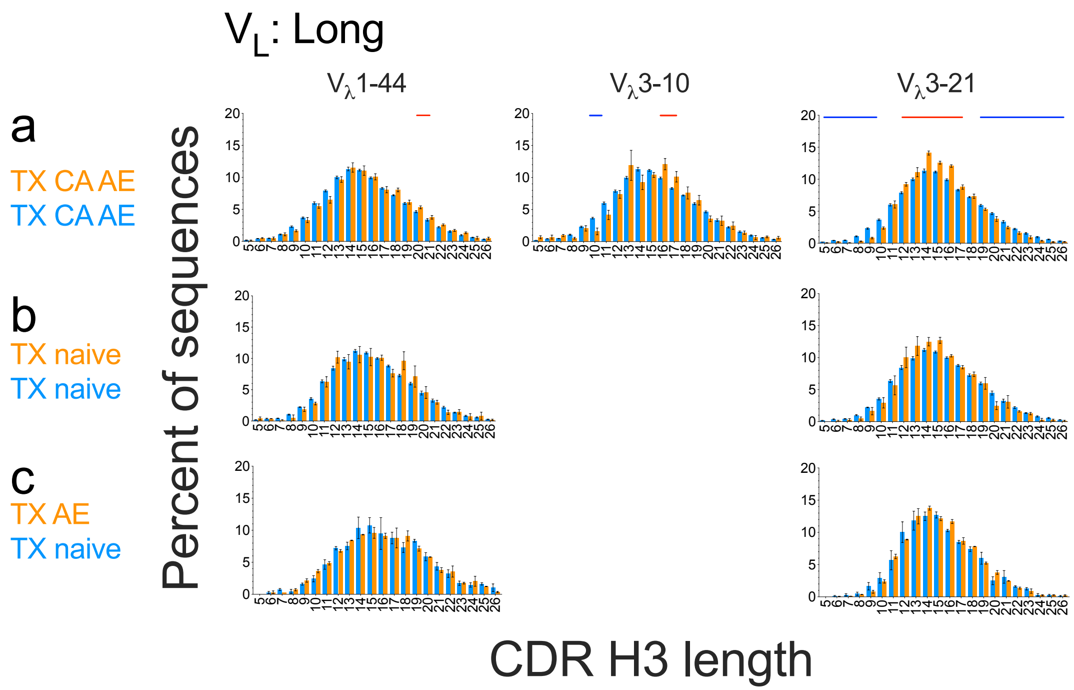

Supplementary Figure 6. CDR H3 length distributions associated with V_L germline segments (orange bars) compared to overall distribution in the same samples and compartments (rows a and b, blue bars) or to corresponding naive sequences (row c):

Row (a) AE sequences, CA and TX donors pooled (n = 5)

Row (b) TX naive compartment (n = 3)

Row (c) TX AE (orange bars, n = 2, same as orange bars in Suppl. Figure 6 TX) and naive (blue bars, same as orange bars in row b) sequences of the indicated germline segment (n = 3)

Red and blue horizontal bars above the histograms in row (a) indicate statistically significant ($P < 10^{-2}$) differences between the germline segment-specific and overall distributions in a paired (within donors, n = 5 donors) *t*-test with a rolling window of 2 consecutive CDR H3 lengths (see main text for details). Error bars indicate S.E.M. except for orange bars in row (c), where it indicates range (n = 2). Arrows indicate subtle deviations in the distributions. Figure tags refer to each row of graphs.

Supplementary Figure 7

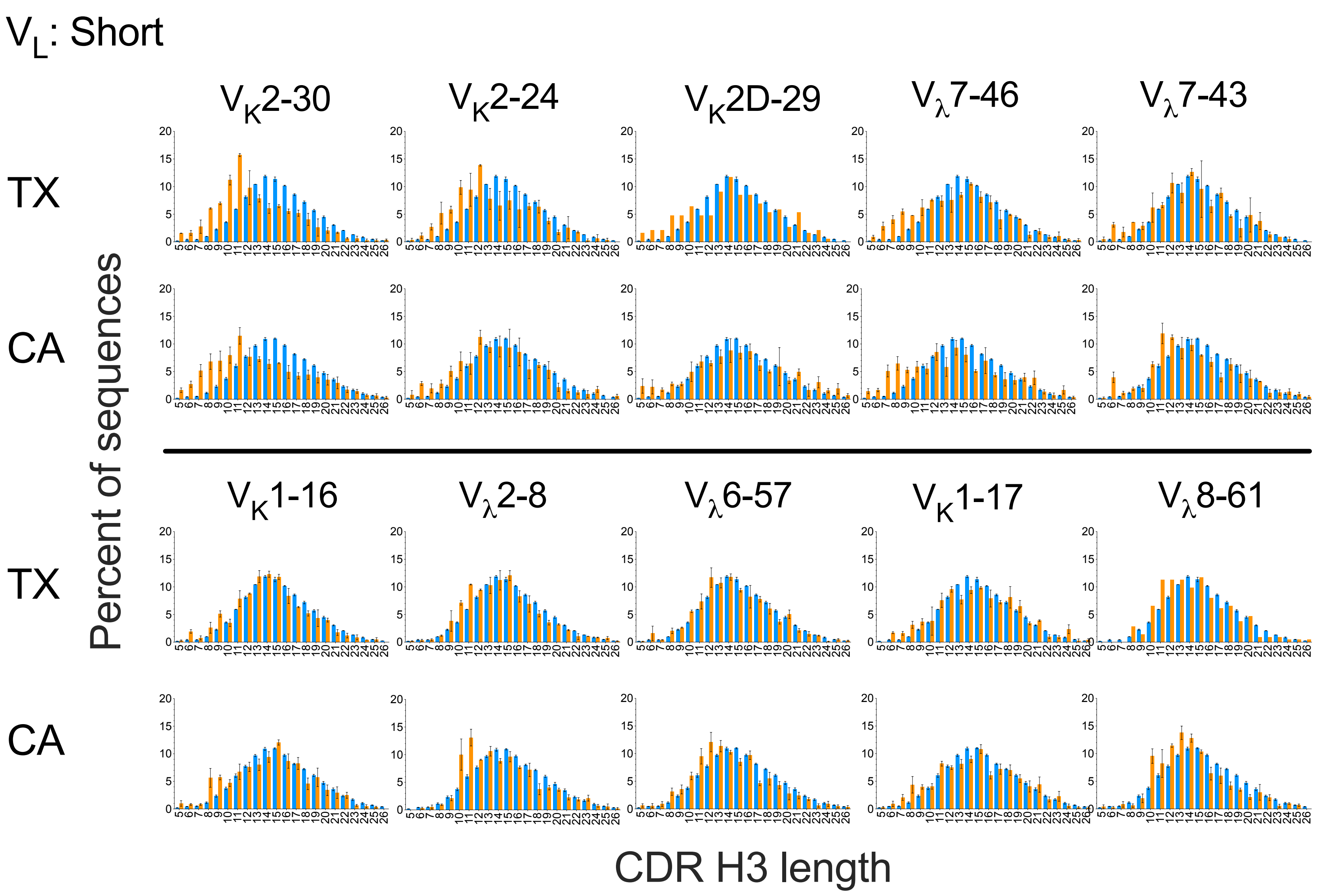

Supplementary Figure 7

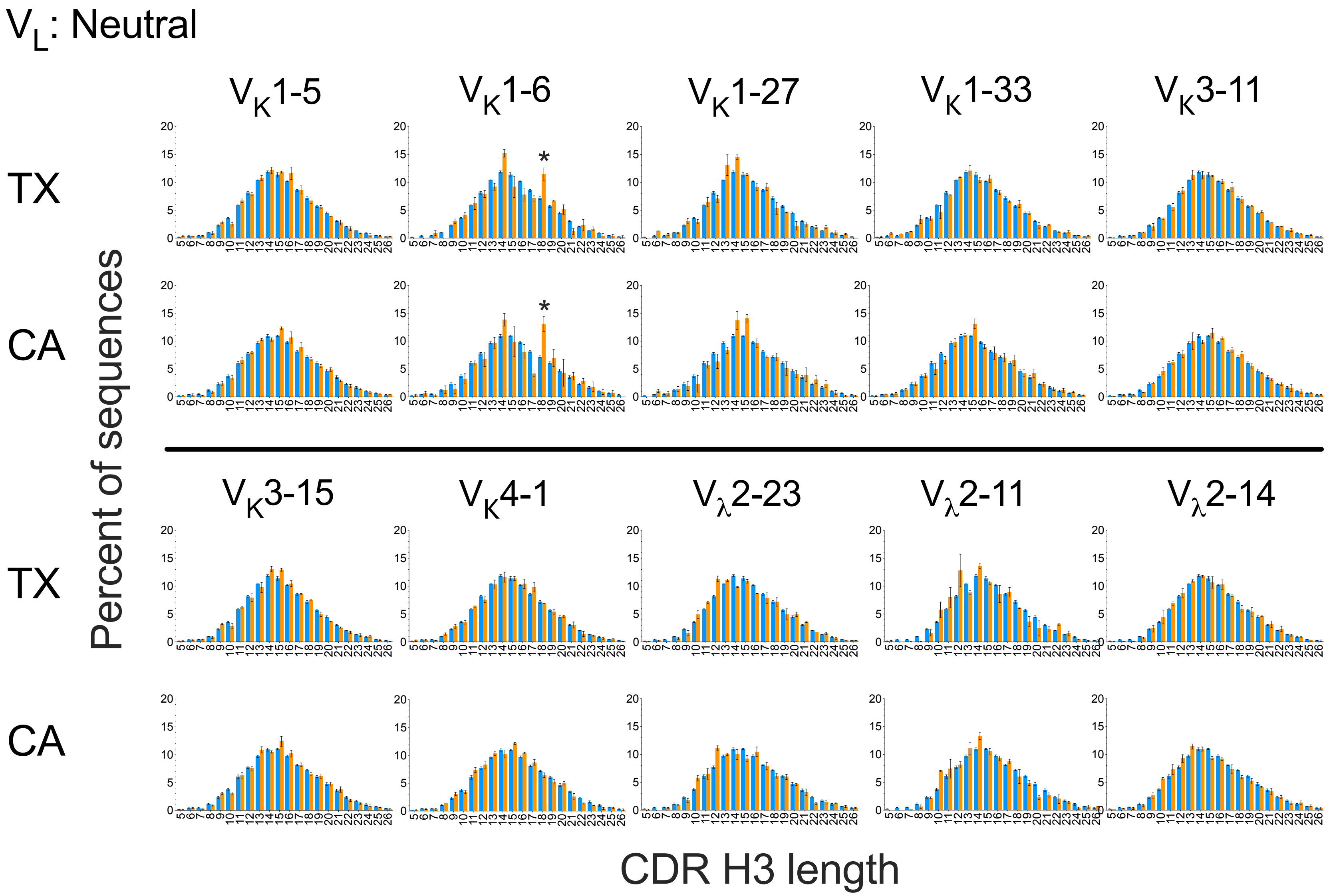

Supplementary Figure 7

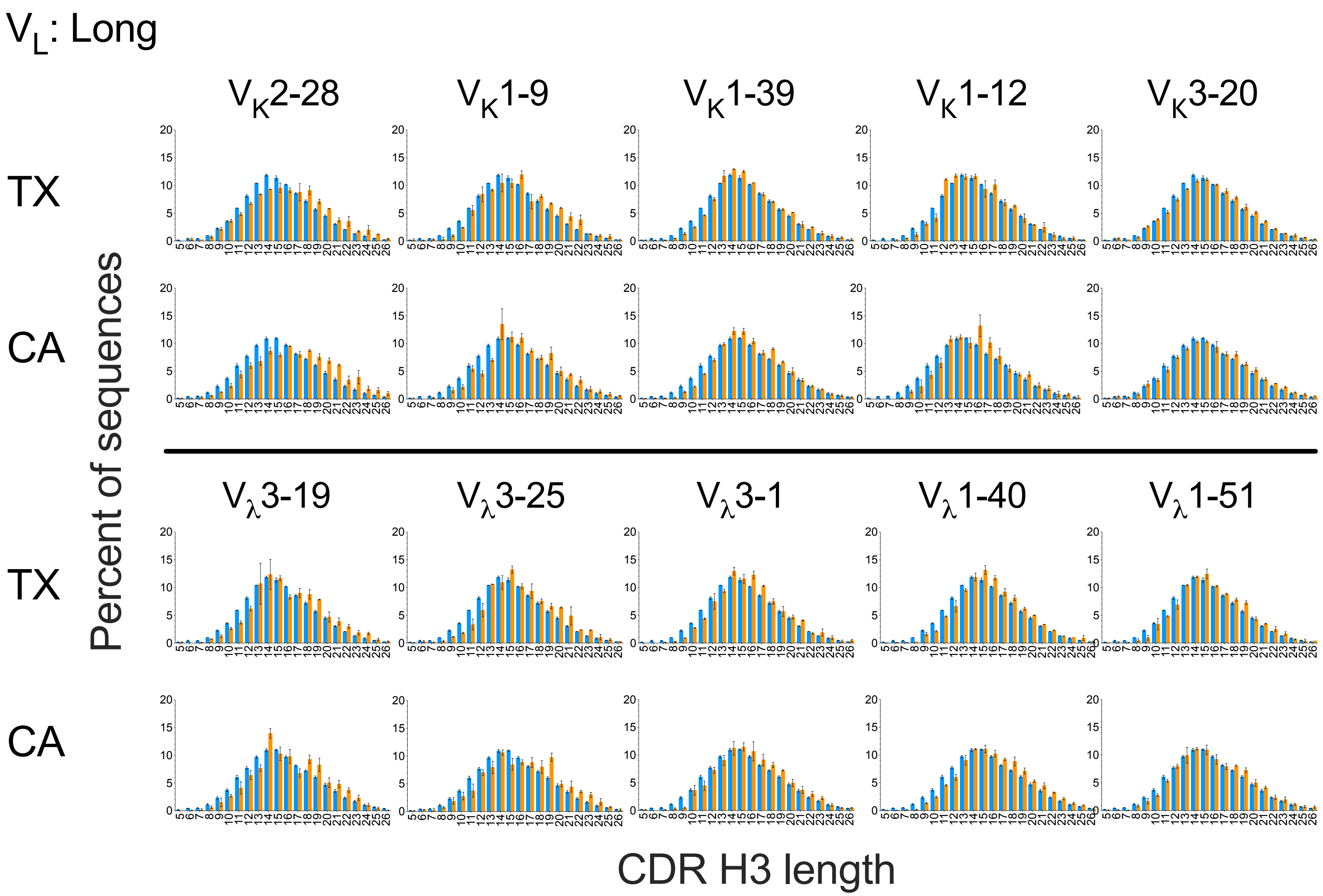

Supplementary Figure 7

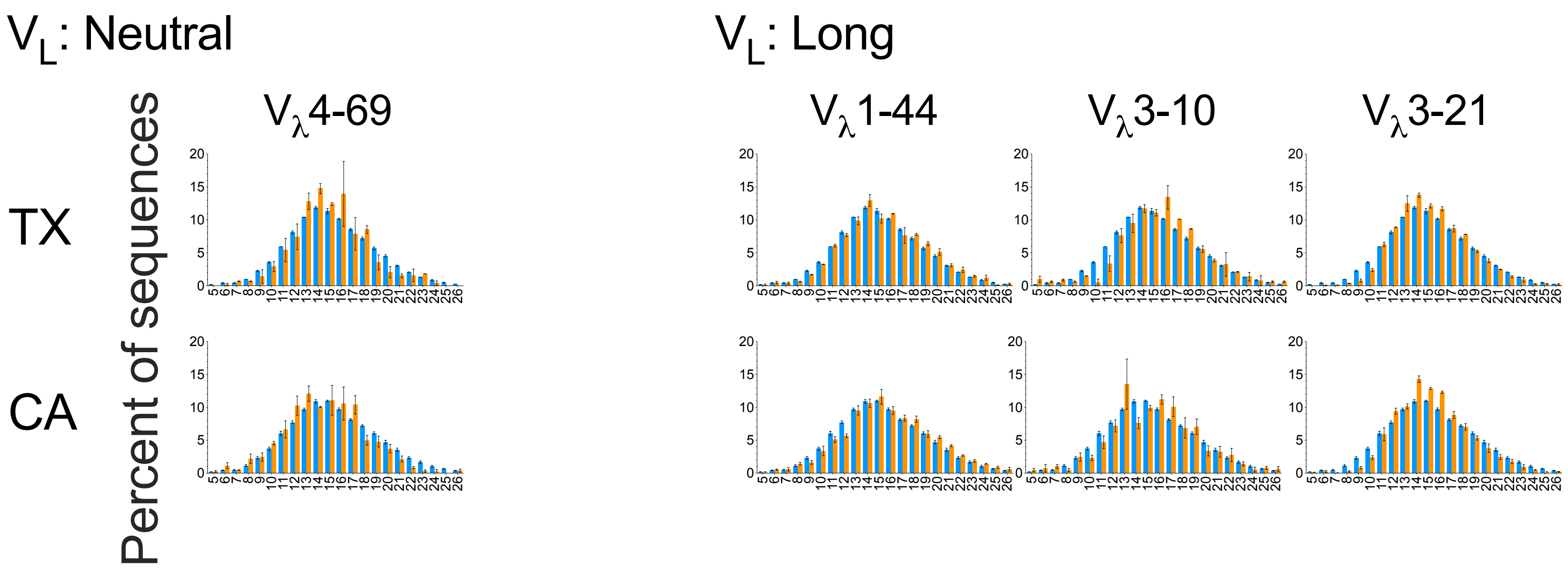

Supplementary Figure 7. V_L-associated CDR H3 length biases are comparable between the CA and TX (AE compartment) datasets. V_L germline segment-specific CDR H3 length distributions (orange bars) compared to overall distribution (blue bars) in the four datasets. Error bars indicate S.E.M. (CA, n = 3) and range (TX, n = 2). Germline segments are named in the IMGT convention. Asterisks indicate spikes at length 18 in the IGKV1-6 distributions of TX and CA datasets with high prevalence of convergent clones with IGHV3-7, IGHJ3, CDR H3 consensus sequence (A/V)RDX_7(L/I/V)(W/Y)YDAFDI and light chain Arg-116 in CDR L3, observed in all CA and TX donors.

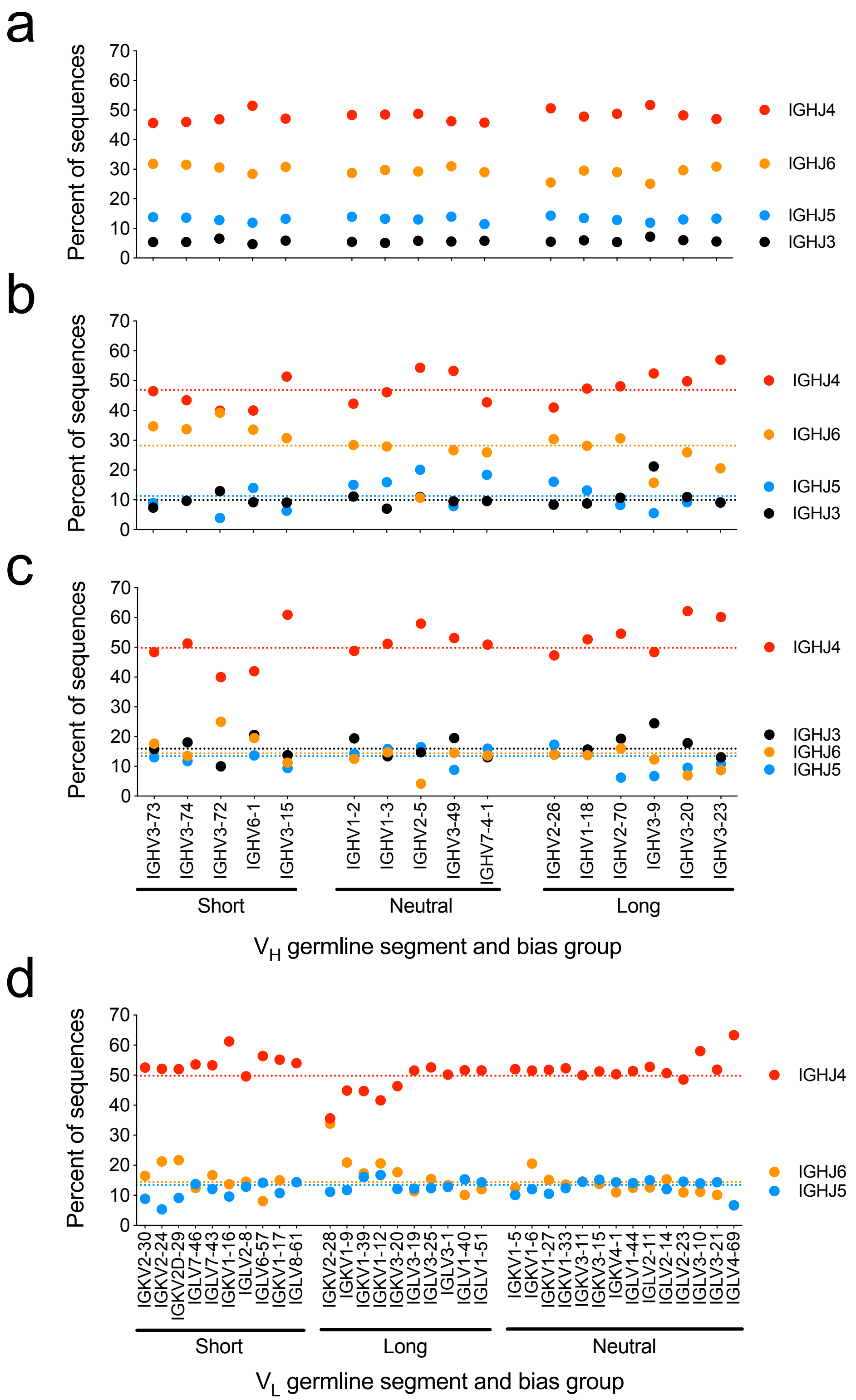

Supplementary Figure 8. J_H prevalence as a function of V_H (a-c) and V_L (d) germline segment in WA unproductive sequences (a) and WA (b) and TX (c and d) naive compartment sequences. Dotted lines indicate overall average J_H prevalence. Germline segments are noted in the IMGT naming convention. Values shown for each dataset after pooling of donors.

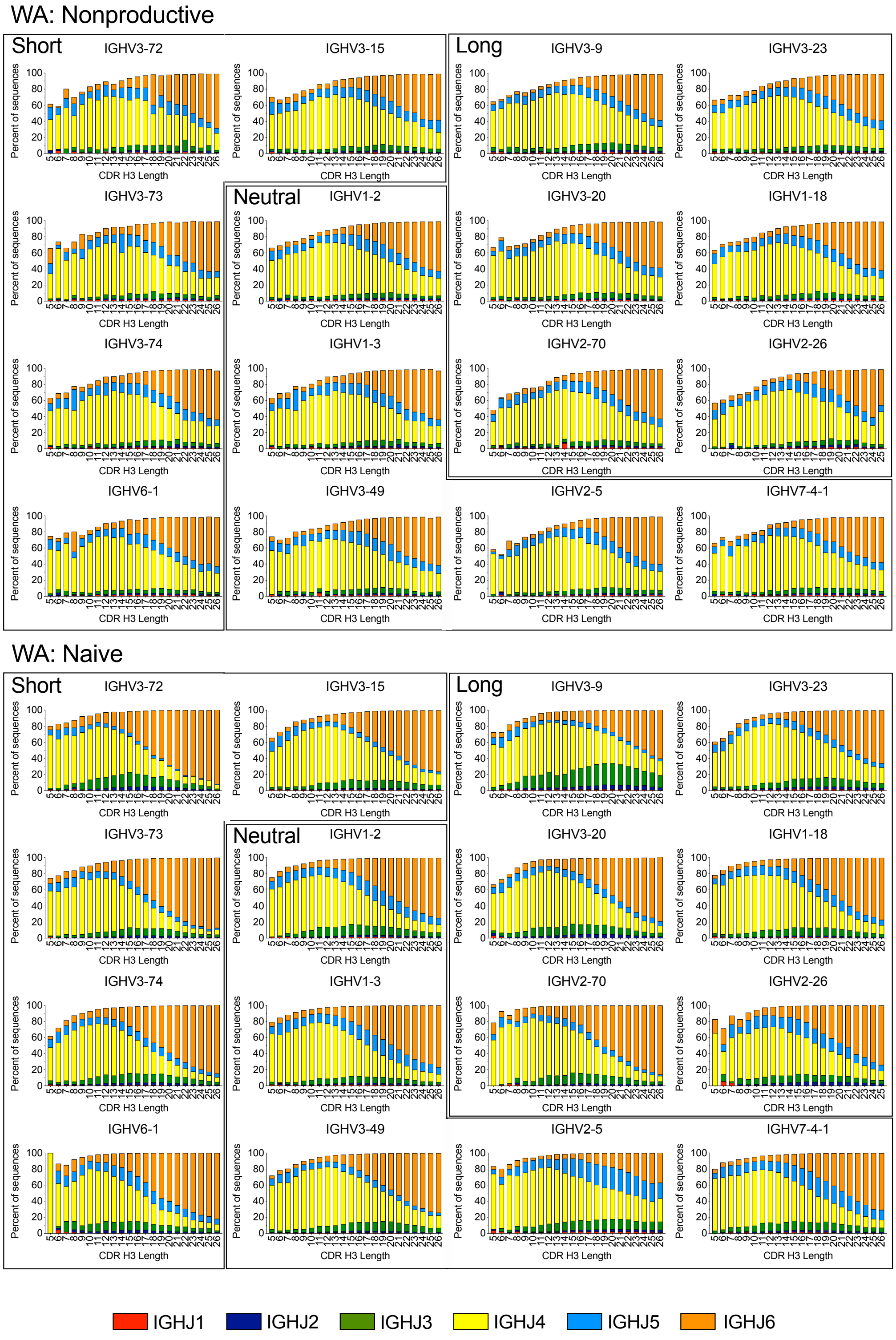

Supplementary Figure 9. J_H germline segment distribution as a function of V_H germline segment and CDR H3 length in unproductive sequences and in the naive compartments of the WA dataset. Data is averaged for all donors. Germline segments are noted in the IMGT naming convention. A fraction of the sequences does not have an unambiguously annotated J_H segment, particularly in the short end of the spectrum. Values calculated after pooling of sequences from 3 donors.

Supplementary Figure 10

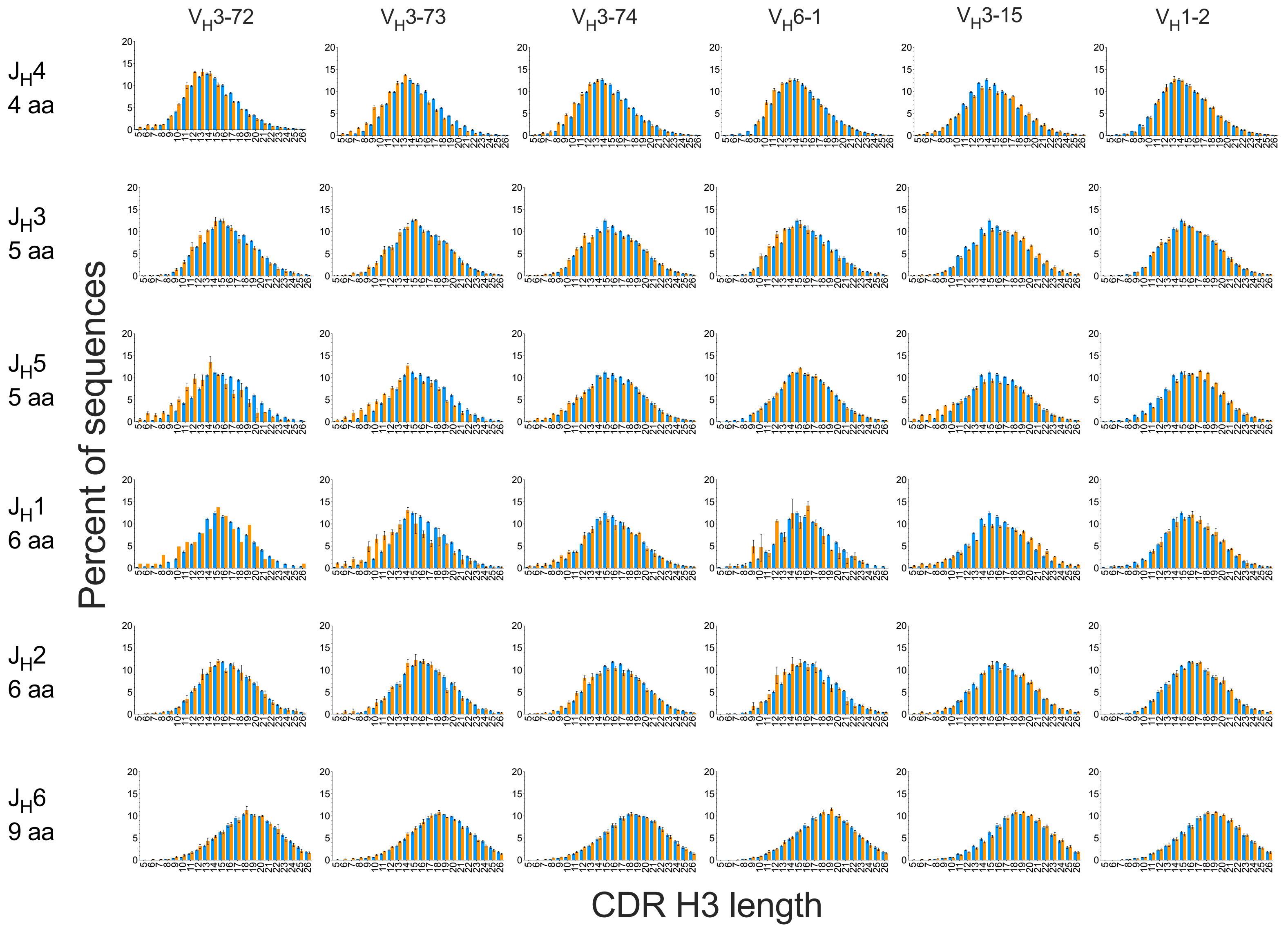

Supplementary Figure 10

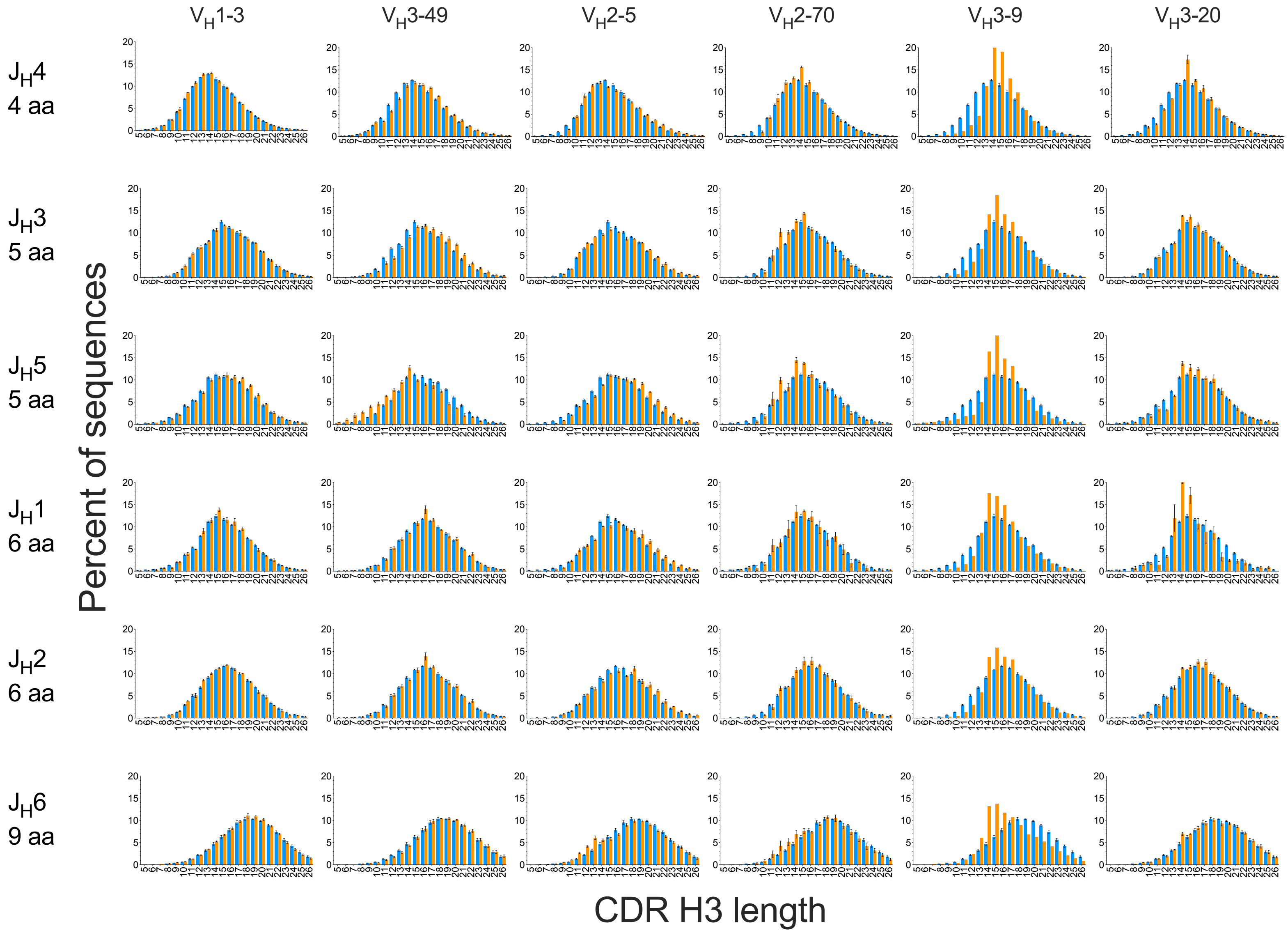

Supplementary Figure 10

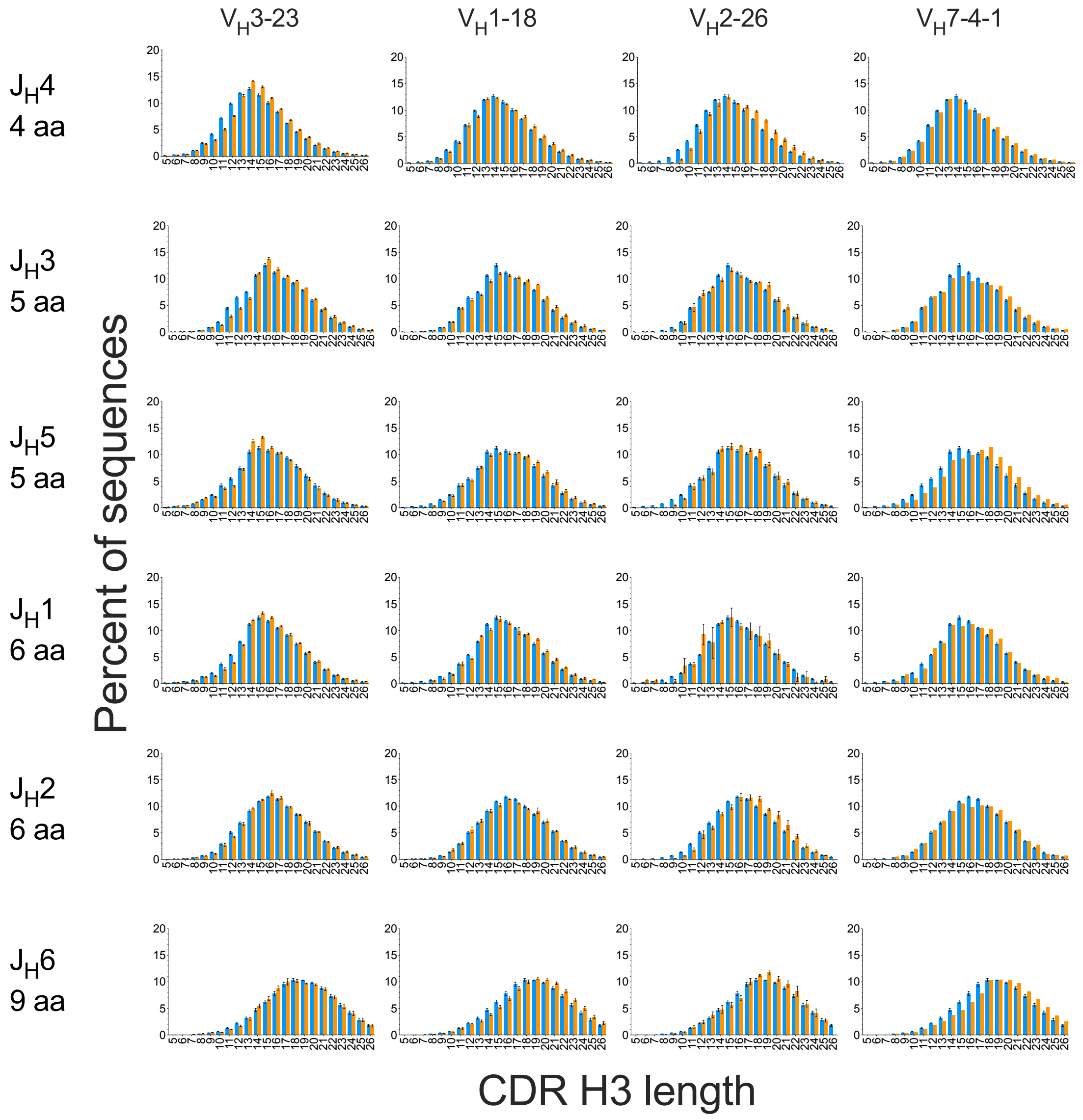

Supplementary Figure 10. CDR H3 distribution as a function of V_H and J_H germline segment in the WA naive compartment. V_H/J_H combination-specific distributions are shown in orange and JH-specific distributions in blue. V_H germline segments are noted in the IMGT naming convention. The number of CDR H3 amino acid residues potentially encoded by J_H1-J_H6 are shown in row legends. Error bars indicate S.E.M. (n = 3 donors except for IGHV3-9 and IGHV7-4-1, with n = 1 donor each).

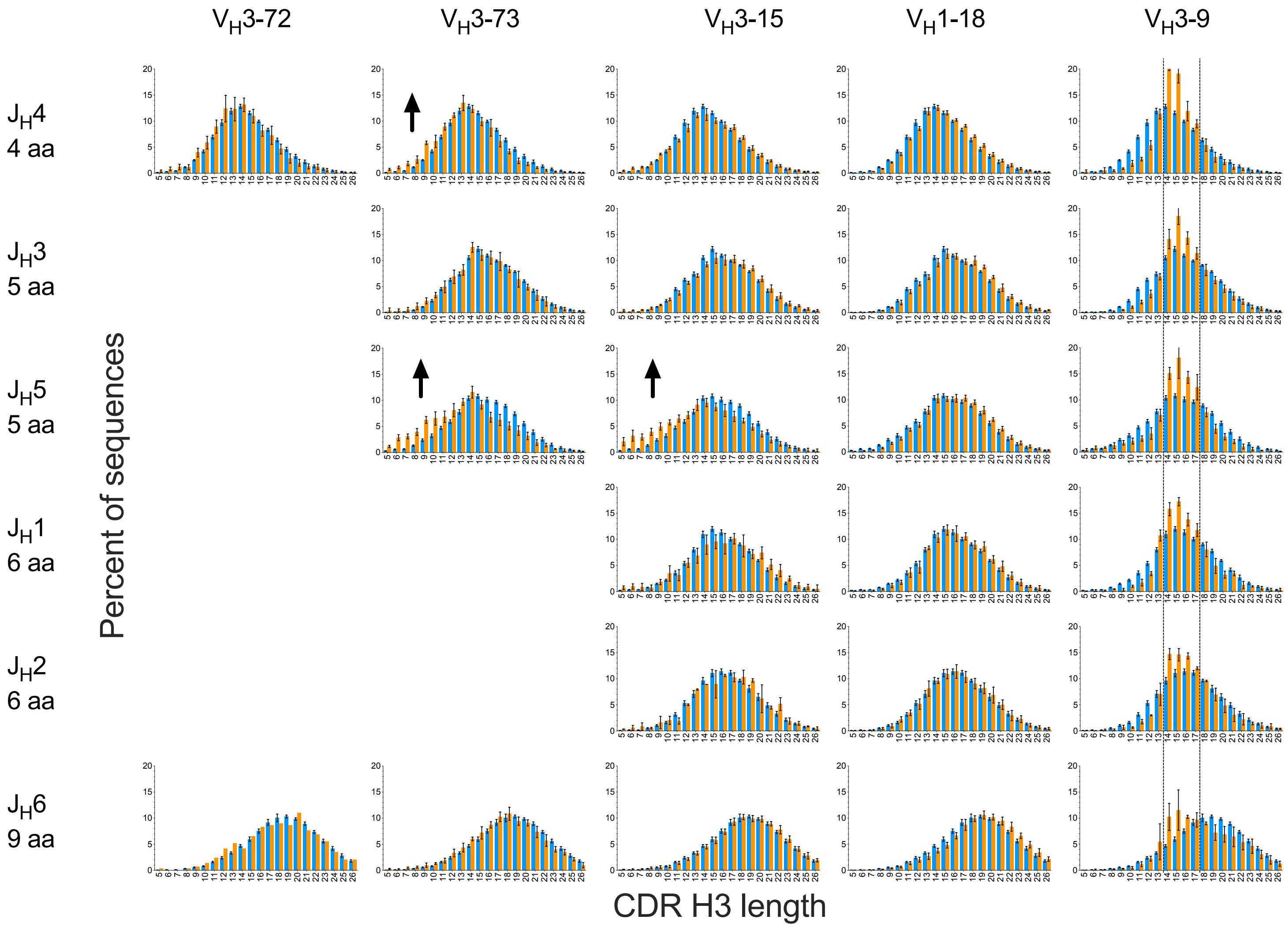

Supplementary Figure 11. Modulation of V_H-associated CDR H3 length biases by J_H segments in the SRI naive compartment. Colors, arrows and panel organization is the same as in Figure 5, adding J_H1 and J_H2, for ease of comparison. Omitted panels had low sequence counts. The number of CDR H3 amino acid (aa) residues potentially encoded by J_H1-J_H6 are shown in row legends. Error bars indicate S.E.M. (n = 8 donors except V_H3-9, n = 7 donors).

Supplementary Figure 12

IGHJ4: YFDY (IMGT 114-117) WA, nonproductive sequences

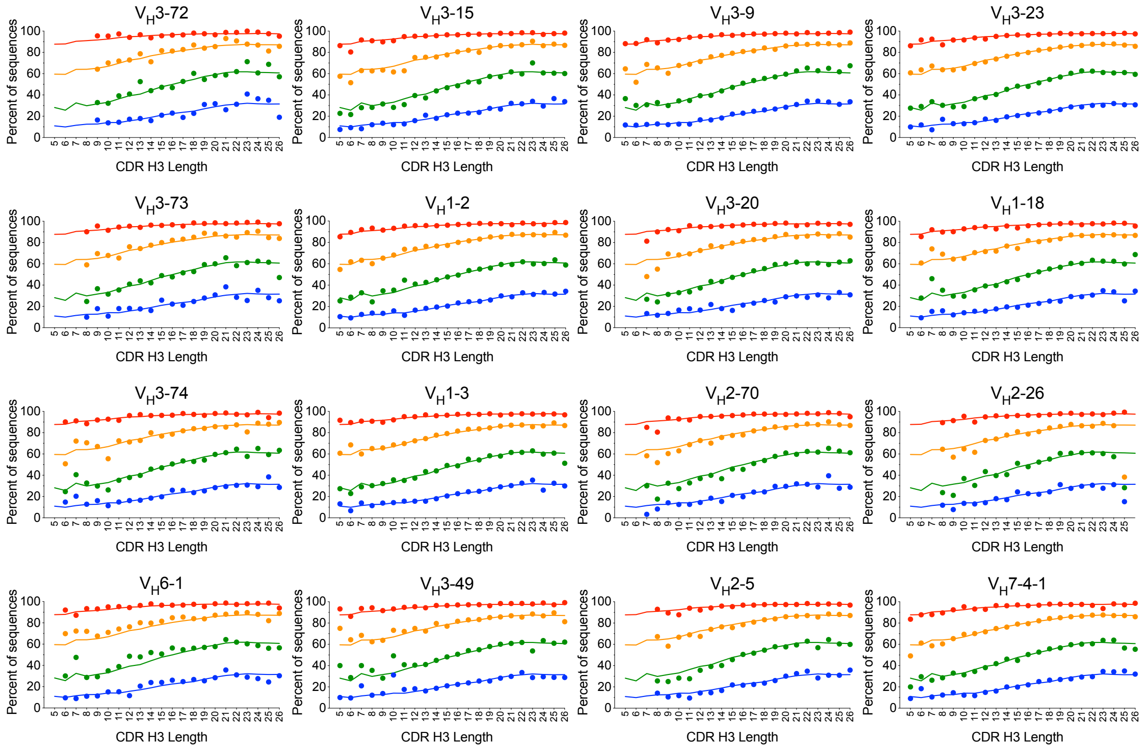

IGHJ4: YFDY (IMGT 114-117) WA, naive sequences

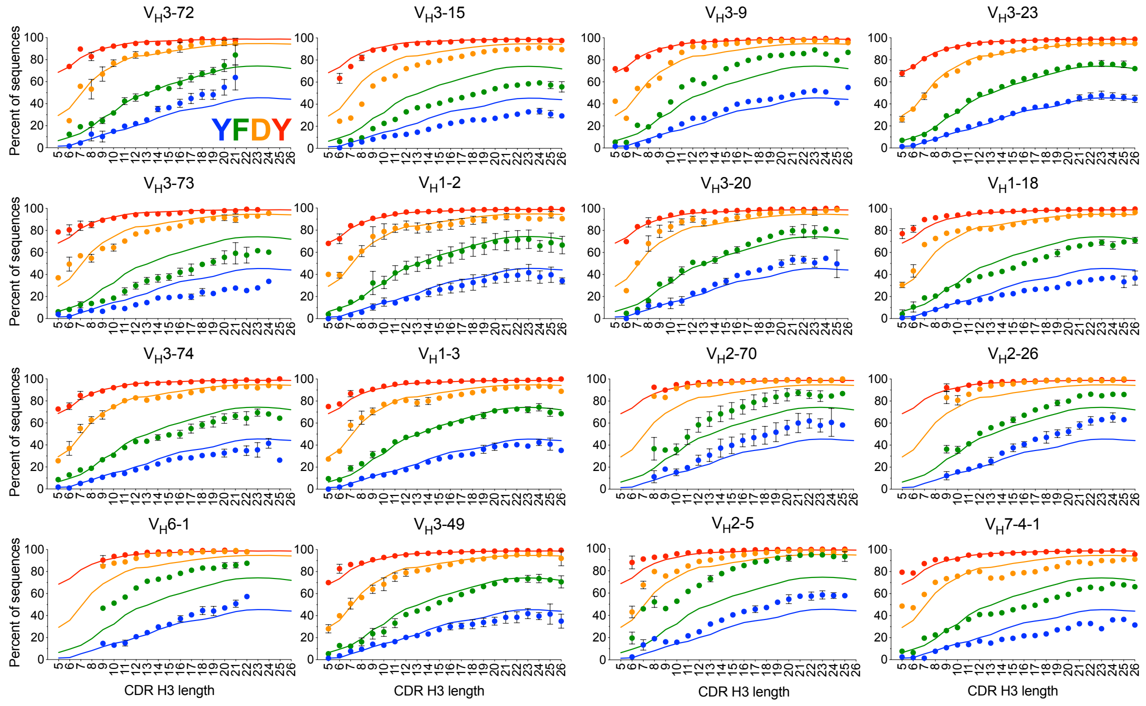

Supplementary Figure 12

IGHJ5: **NWFDP** (IMGT 113-117) WA, Nonproductive sequences

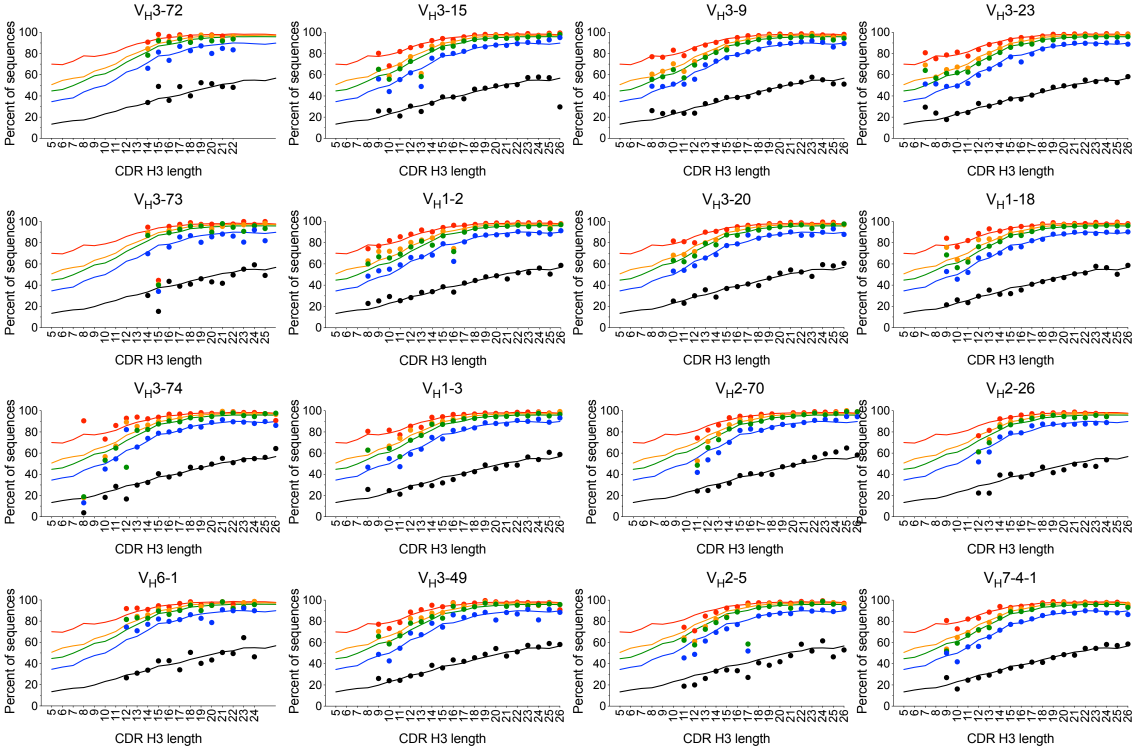

IGHJ5: **NWFDP** (IMGT 113-117) WA, Naive sequences

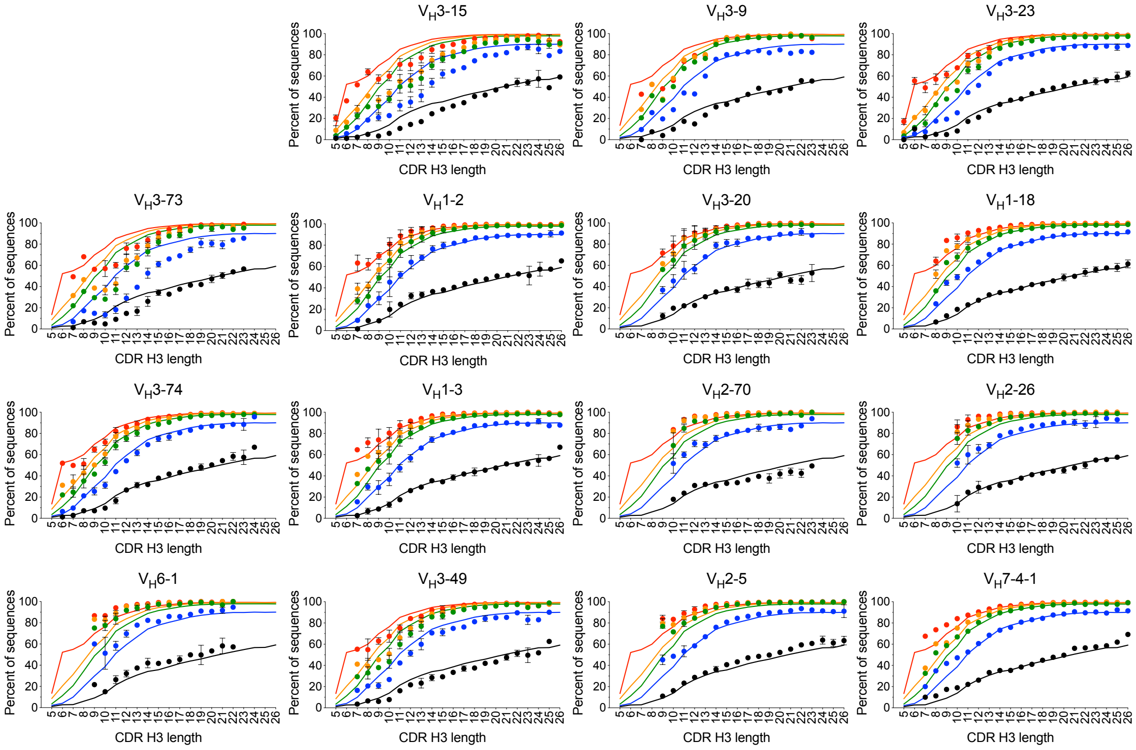

Supplementary Figure 12

Supplementary Figure 12. Analysis of J_H4 and J_H5 sequence trimming as a function of V_H germline segment use and CDR H3 length in WA unproductive and naive compartment sequences. Occupancy of J_H germline segment-encoded residues in the last CDR H3 positions are shown. “YFDY” and “NWFDP” refer to the last residues of the J_H4 and J_H5 germline segments within CDR H3, color coded as in the lines in the figure. Solid lines indicate residue occupancy for all sequences in the unproductive and naive repertoire with a given J_H segment. Dots indicate average residue occupancy with each V_H/J_H combination CDR H3 length. Dots above and below the corresponding solid line indicate reduced and increased J_H trimming relative to the overall repertoire. Note that trimming of J_H segments in the non-productive sequences is not biased by V_H. Bars indicate S.E.M. for three donors except for V_H3-9 and V_H7-4-1, which are present in one donor each, and the nonproductive sequences, which were pooled before analysis. Data points with fewer than 60 sequences (30 for V_H3-9 and V_H7-4-1) were excluded. Panels are organized in the same order as in Supplementary Figure 9.

IGHJ4: YFDY (IMGT 114-117)   SRI, Naive compartment

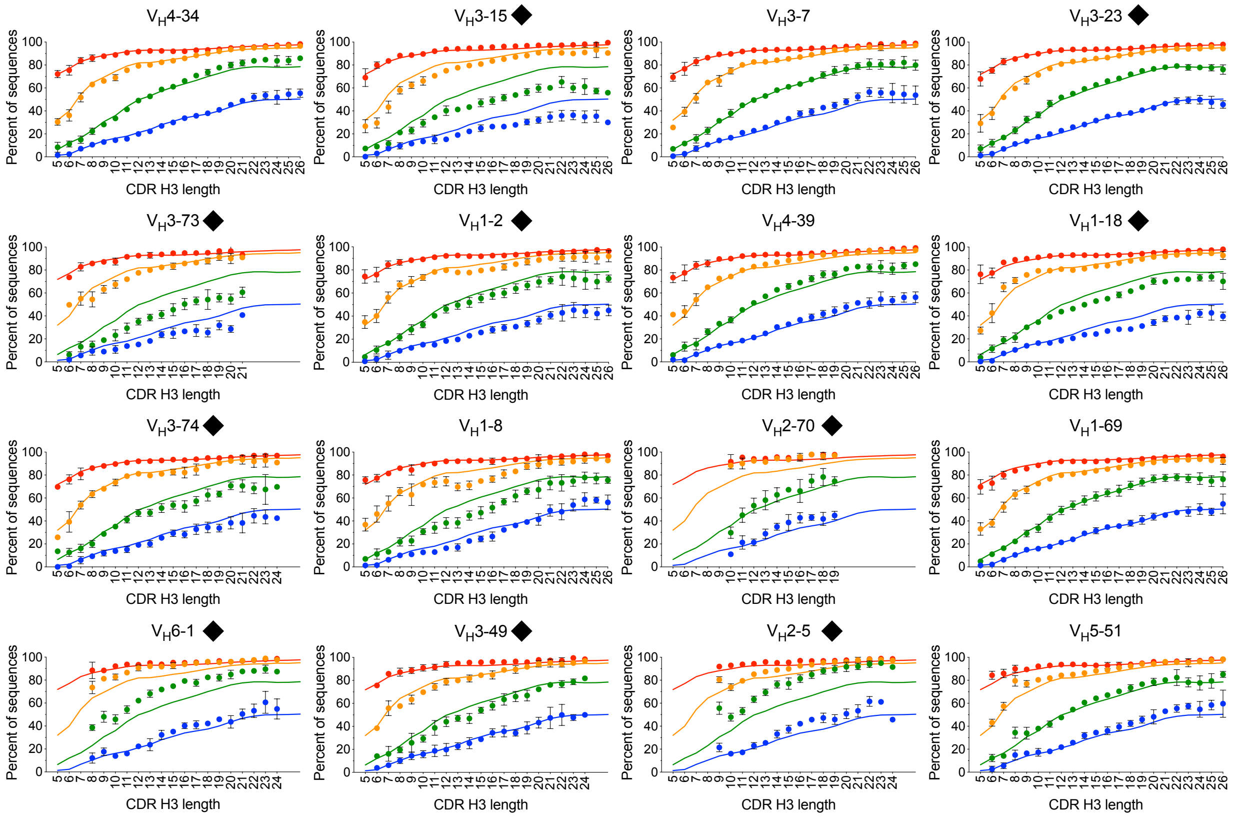

Supplementary Figure 13. Analysis of J_H4 sequence trimming as a function of V_H germline segment use and CDR H3 length in SRI naive compartment sequences. Symbols shown as in Figure 6. The panels highlighted with black diamonds indicate germline segments in the same place as in Suppl. Fig. 12. Other panels show different germline segments according to data availability in the dataset. Data points with fewer than 60 sequences were excluded. “YFDY” refers to the last residues of the J_H4 germline segment within CDR H3, color coded as in the lines in the figure. Error bars indicate S.E.M. (n = 7 except V_H2-70, n=5, and V_H1-8 and V_H6-1, n = 6 donors).

IGHJ3

D germline length (nt)	Total	VH3-72	VH3-73	VH3-74	VH6-1	VH3-15	VH1-2	VH1-3	VH3-49	VH3-9	VH3-20	VH2-5	VH2-70	VH3-23	VH1-18	VH2-26	VH7-4-1
37	1.7%	2.1%	1.9%	1.5%	2.7%	1.5%	2.2%	1.7%	1.4%	1.8%	1.8%	1.5%	1.9%	1.4%	1.8%	1.6%	1.8%
31	24.7%	27.4%	25.2%	23.0%	23.4%	25.9%	27.9%	23.5%	24.1%	26.8%	23.9%	20.0%	22.1%	23.9%	26.2%	18.3%	23.3%
28	0.5%	0.6%	0.4%	0.5%	0.4%	0.7%	0.7%	0.6%	0.6%	0.1%	0.5%	0.5%	0.4%	0.5%	0.6%	0.5%	1.5%
23	8.1%	8.3%	7.1%	8.1%	6.6%	8.6%	7.4%	11.0%	8.1%	9.6%	8.4%	9.1%	8.1%	8.3%	8.3%	9.5%	8.0%
21	15.1%	15.3%	12.4%	15.6%	12.7%	15.3%	12.5%	14.4%	15.8%	12.2%	13.3%	13.9%	14.9%	17.4%	14.8%	16.7%	5.7%
20	19.1%	19.5%	23.1%	20.5%	11.3%	24.2%	16.7%	17.8%	23.6%	13.4%	21.3%	24.6%	16.5%	19.2%	20.0%	22.0%	26.7%
19	1.8%	1.6%	2.1%	1.7%	1.4%	1.3%	1.6%	1.1%	1.4%	1.9%	1.5%	1.4%	2.2%	1.5%	1.5%	2.0%	0.5%
18	3.0%	2.7%	2.5%	2.7%	3.2%	2.6%	2.9%	3.0%	3.0%	2.7%	3.4%	2.9%	3.0%	2.5%	2.6%	3.7%	4.7%
17	9.7%	7.7%	8.2%	8.9%	9.5%	6.9%	9.4%	9.3%	8.6%	13.8%	9.0%	9.6%	11.1%	9.4%	8.3%	9.9%	6.8%
16	9.8%	7.7%	10.0%	9.0%	16.6%	7.2%	10.4%	11.0%	8.4%	11.7%	10.8%	8.5%	11.6%	10.3%	9.8%	9.3%	13.8%
11	1.3%	1.2%	1.3%	1.5%	4.0%	1.0%	1.9%	1.1%	0.9%	1.6%	1.0%	1.7%	1.6%	1.1%	1.2%	1.4%	1.1%
Weighted average (nt)	21.5	21.9	21.5	21.0	20.4	22.0	21.6	21.4	21.8	21.7	21.5	20.8	20.8	21.6	21.8	20.9	21.1

IGHJ4

D germline length (nt)	Total	VH3-72	VH3-73	VH3-74	VH6-1	VH3-15	VH1-2	VH1-3	VH3-49	VH3-9	VH3-20	VH2-5	VH2-70	VH3-23	VH1-18	VH2-26	VH7-4-1
37	4.4%	4.6%	4.1%	4.5%	4.1%	4.7%	4.5%	4.6%	4.2%	3.0%	4.7%	4.8%	4.1%	4.2%	4.4%	4.5%	5.5%
31	40.3%	37.6%	34.6%	38.2%	24.4%	47.4%	38.0%	39.8%	45.0%	38.6%	40.4%	42.5%	33.8%	43.0%	41.8%	43.0%	40.4%
28	2.1%	2.5%	2.5%	2.3%	1.6%	2.2%	1.8%	1.8%	2.4%	1.2%	1.7%	1.7%	1.9%	2.3%	2.0%	2.0%	1.4%
23	4.0%	4.2%	3.7%	4.0%	4.1%	3.6%	4.2%	4.1%	3.8%	3.4%	3.9%	3.9%	3.9%	3.3%	3.7%	4.6%	5.1%
21	14.8%	10.4%	14.7%	13.7%	26.9%	10.2%	15.5%	18.1%	12.8%	20.8%	17.1%	14.1%	18.1%	15.3%	15.2%	14.8%	16.5%
20	12.8%	16.8%	13.7%	14.2%	12.8%	11.5%	11.8%	12.1%	12.7%	15.0%	10.9%	10.0%	14.9%	12.6%	12.6%	13.3%	8.4%
19	1.6%	1.8%	2.3%	1.7%	1.5%	1.1%	1.4%	1.1%	1.3%	1.7%	1.2%	1.2%	1.9%	1.4%	1.4%	1.5%	1.0%
18	3.7%	2.3%	3.8%	3.5%	5.1%	2.2%	5.1%	3.2%	3.0%	4.4%	2.8%	4.0%	4.3%	3.3%	3.5%	2.6%	3.7%
17	3.3%	4.1%	3.9%	3.2%	5.5%	3.6%	4.3%	3.3%	3.0%	3.1%	2.8%	3.7%	3.6%	2.7%	3.4%	2.8%	3.0%
16	8.0%	9.7%	10.8%	8.0%	5.5%	8.4%	8.0%	6.9%	7.6%	4.8%	10.4%	8.7%	8.4%	7.1%	7.7%	7.4%	7.6%
11	0.9%	1.0%	1.0%	1.1%	2.9%	0.7%	1.3%	0.8%	0.7%	1.0%	0.7%	1.1%	1.2%	0.8%	0.8%	0.9%	2.4%
Weighted average (nt)	24.2	23.7	23.2	23.7	22.0	25.0	23.9	24.2	24.9	24.1	24.4	24.4	23.4	24.6	24.5	24.9	24.1

IGHJ5

D germline length (nt)	Total	VH3-72	VH3-73	VH3-74	VH6-1	VH3-15	VH1-2	VH1-3	VH3-49	VH3-9	VH3-20	VH2-5	VH2-70	VH3-23	VH1-18	VH2-26	VH7-4-1
37	4.2%	5.6%	4.1%	4.3%	4.1%	4.3%	4.2%	4.2%	3.6%	3.3%	4.8%	4.2%	4.8%	3.9%	4.1%	4.1%	3.9%
31	47.9%	38.8%	42.5%	47.2%	32.1%	53.7%	47.4%	49.3%	50.7%	42.4%	43.7%	52.5%	41.0%	47.6%	49.1%	46.6%	49.8%
28	1.9%	3.2%	2.2%	1.9%	2.1%	2.2%	1.4%	1.7%	2.1%	1.1%	1.5%	1.5%	2.1%	1.9%	1.6%	1.8%	1.2%
23	2.4%	3.3%	2.7%	2.5%	3.0%	2.4%	2.5%	2.6%	2.4%	2.2%	2.2%	2.1%	2.6%	2.2%	2.3%	2.5%	3.2%
21	13.5%	10.2%	14.2%	13.7%	24.2%	9.1%	13.6%	15.7%	12.6%	20.0%	16.3%	12.6%	15.4%	14.9%	13.7%	16.1%	16.0%
20	7.9%	11.6%	9.3%	8.9%	8.9%	7.6%	7.4%	7.2%	8.3%	10.8%	7.6%	5.9%	8.8%	8.3%	8.0%	8.2%	4.8%
19	1.0%	1.1%	1.2%	1.0%	1.0%	0.8%	0.9%	0.8%	0.9%	1.0%	0.9%	0.7%	1.2%	0.9%	0.9%	1.2%	0.8%
18	4.1%	2.0%	3.6%	3.4%	5.1%	2.2%	5.5%	3.4%	3.3%	4.3%	3.1%	4.0%	4.7%	3.7%	3.8%	3.4%	4.2%
17	4.0%	5.5%	3.9%	3.3%	7.2%	3.8%	5.1%	3.9%	3.7%	4.6%	3.9%	3.9%	4.7%	3.6%	4.2%	3.8%	4.7%
16	7.5%	10.5%	9.3%	6.7%	5.3%	6.8%	7.0%	6.4%	7.1%	5.0%	11.0%	7.2%	8.5%	7.5%	7.0%	7.2%	6.2%
11	0.7%	0.7%	0.7%	0.7%	1.6%	0.6%	0.8%	0.5%	0.6%	0.8%	0.8%	0.7%	0.9%	0.7%	0.6%	0.7%	0.8%
Weighted average (nt)	24.8	23.5	23.9	24.5	23.0	25.2	24.8	25.2	25.1	24.2	24.4	25.3	24.0	24.8	24.9	24.8	25.1

IGHJ6

D germline length (nt)	Total	VH3-72	VH3-73	VH3-74	VH6-1	VH3-15	VH1-2	VH1-3	VH3-49	VH3-9	VH3-20	VH2-5	VH2-70	VH3-23	VH1-18	VH2-26	VH7-4-1
37	4.1%	4.3%	3.8%	3.8%	3.8%	3.8%	4.2%	4.0%	3.8%	4.6%	5.1%	4.5%	4.1%	4.0%	3.7%	3.9%	4.1%
31	46.6%	45.1%	43.3%	47.1%	32.0%	50.0%	45.4%	47.8%	49.5%	40.8%	47.1%	45.9%	38.6%	47.6%	50.3%	45.8%	51.1%
28	1.8%	2.0%	2.0%	1.9%	1.7%	1.8%	1.5%	1.6%	1.8%	1.4%	1.5%	1.3%	1.6%	1.8%	1.7%	1.6%	1.0%
23	3.3%	3.2%	3.1%	2.9%	4.3%	3.2%	3.3%	3.4%	3.1%	2.7%	2.9%	2.9%	2.9%	2.8%	3.0%	3.9%	3.7%
21	10.6%	7.9%	10.1%	10.7%	20.1%	8.9%	11.3%	12.0%	10.1%	13.0%	10.8%	9.9%	12.5%	11.3%	11.0%	12.5%	13.0%
20	9.0%	10.8%	9.9%	9.6%	9.7%	8.7%	8.6%	8.4%	9.2%	9.9%	8.0%	7.2%	10.2%	8.9%	8.3%	9.2%	6.6%
19	1.1%	1.4%	1.3%	1.1%	1.1%	0.8%	1.0%	0.9%	1.0%	1.3%	0.8%	0.7%	1.3%	1.0%	0.9%	1.2%	0.7%
18	4.6%	2.9%	4.9%	3.9%	6.1%	3.1%	5.7%	3.9%	3.8%	5.3%	3.5%	5.1%	6.3%	4.0%	3.8%	3.5%	3.4%
17	3.7%	4.4%	3.8%	3.2%	7.1%	4.0%	4.7%	3.9%	3.3%	4.0%	3.6%	3.7%	4.0%	3.3%	3.8%	3.4%	3.1%
16	7.8%	8.9%	10.0%	7.3%	4.8%	8.7%	7.4%	7.2%	7.9%	4.9%	8.8%	8.1%	8.6%	7.9%	7.3%	7.0%	5.8%
11	0.7%	0.7%	0.8%	0.7%	2.0%	0.6%	0.8%	0.7%	0.7%	1.0%	0.8%	0.9%	0.9%	0.8%	0.6%	0.7%	0.7%
Weighted average (nt)	24.2	23.7	23.7	24.1	22.5	24.6	24.2	24.5	24.7	22.8	24.3	23.5	22.8	24.4	24.8	24.1	24.8

Supplementary Figure 14. D germline segment prevalence associated with different V_H and J_H germline segments in the WA naive compartment. D germline segments are grouped by length. The D germline segment prevalence deviations from averages associated with V_H6-1 are boxed.

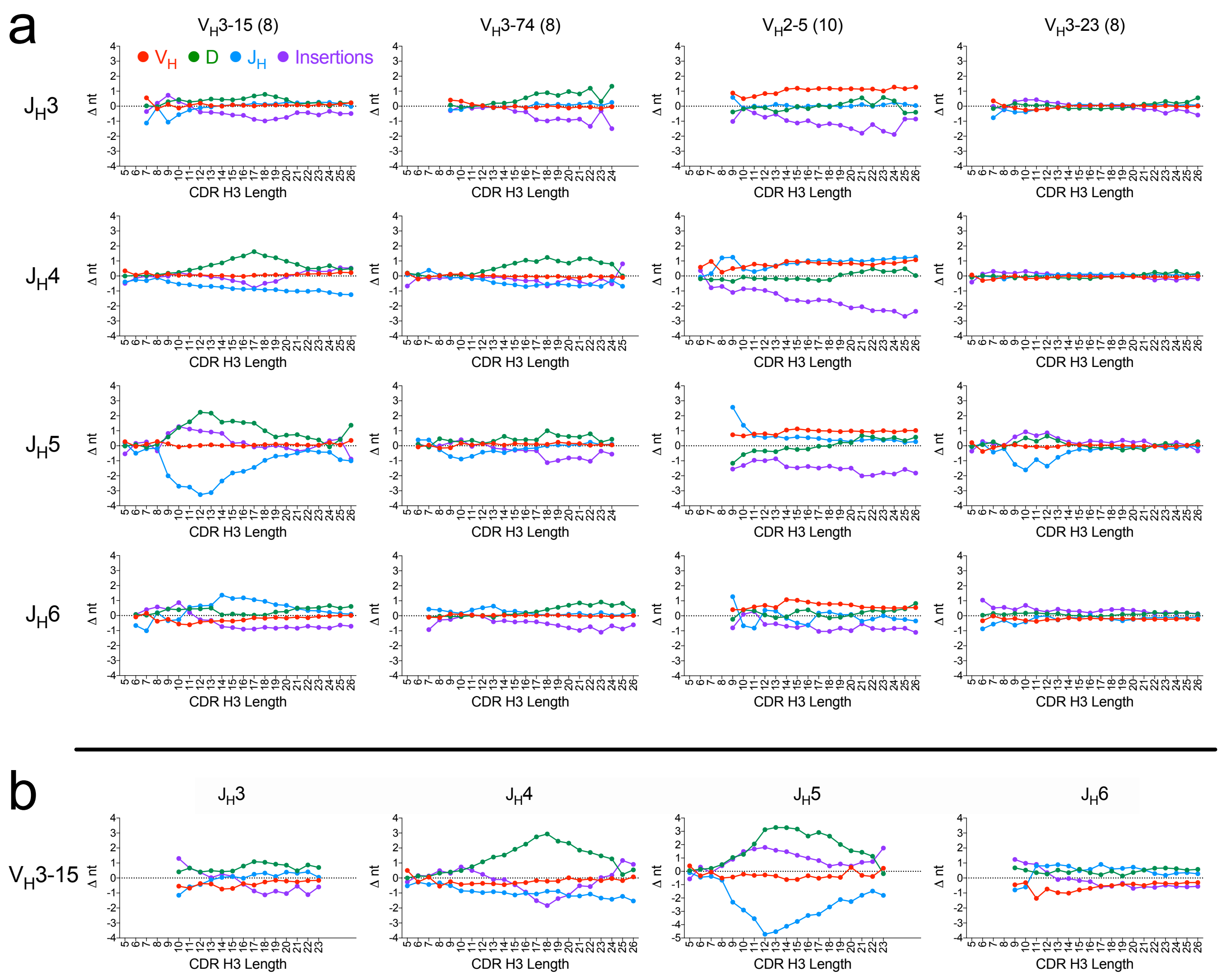

Supplementary Figure 15. Summary of most significant length biases of regions within CDR H3 as a function of V_H and J_H germline use and CDR H3 length. (a, rows 1 to 4) Deviations in nucleotides (Δnt) from whole repertoire averages are shown for the V_H, D_H, J_H segments and NP-region (insertions) in CDR H3 in WA naive sequences grouped by V_H and J_H germlines. Colors for each segment are indicated in the top left panel. Values in parentheses indicate the maximum number of V_H germline nucleotides that can be included in CDR H3. (b, row 5) As in panels (a), showing CDR H3 segment length deviations associated with V_H3-15 sequences and different J_H germline segments in 3 donors of the SRI naive dataset (donors 326650, 326797 and 327059). Sequences with undefined D segments were omitted. Values shown are averages of deviations from overall repertoire, with deviations calculated within donors. Data points with fewer than 60 counts within donors were excluded. Error bars not shown for clarity. CDR H3 lengths are shown in amino acid residues. The full dataset is shown in Supplementary Fig. 16. See Supplementary text for details.

Supplementary Figure 16

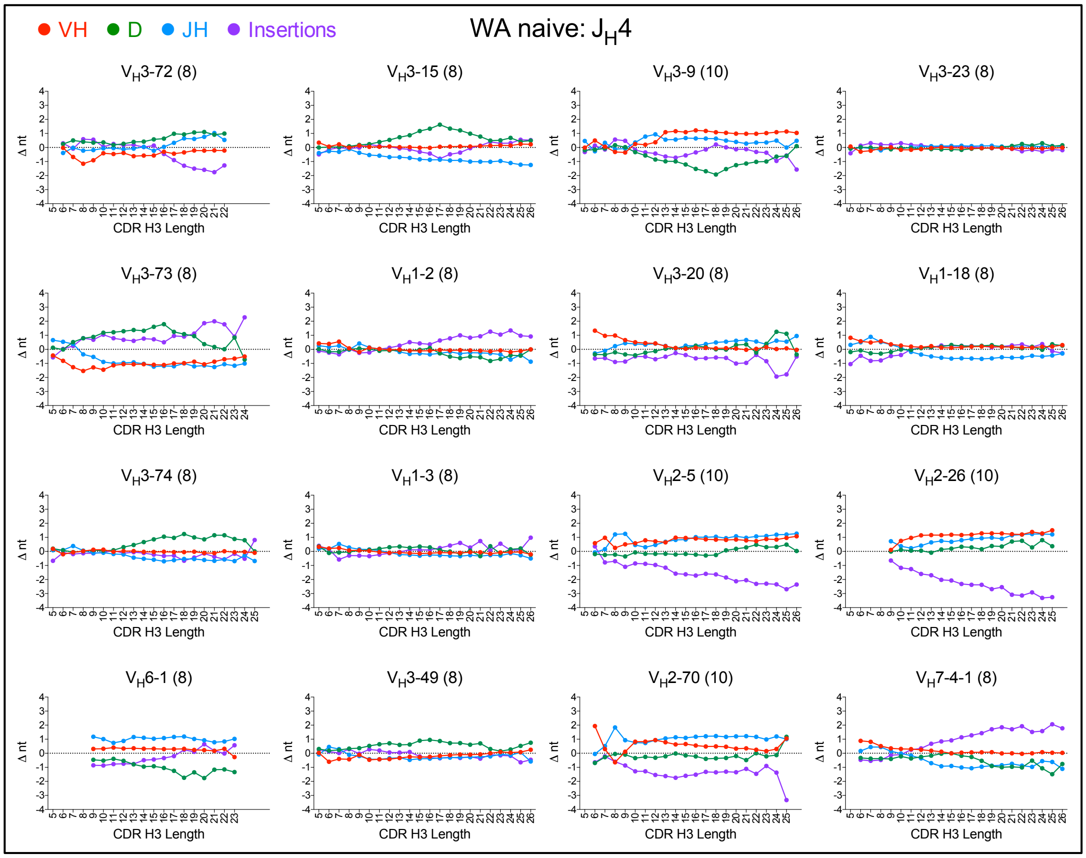

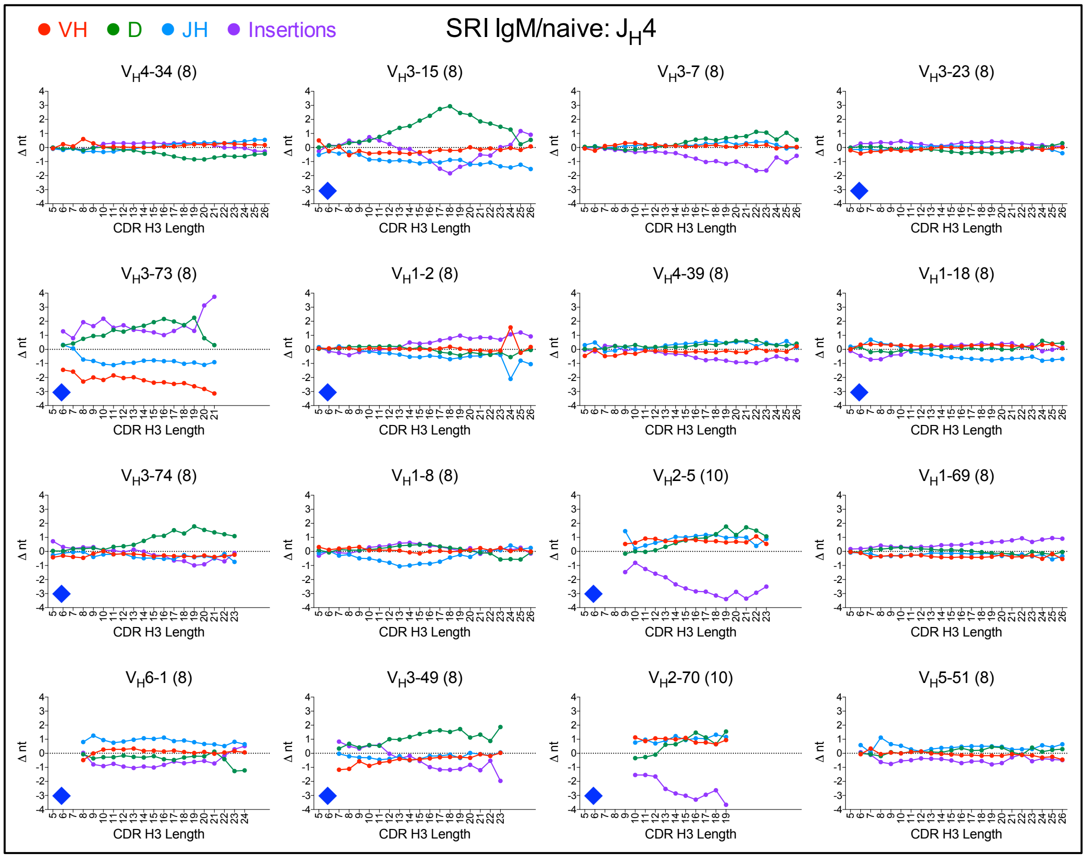

Supplementary Figure 16

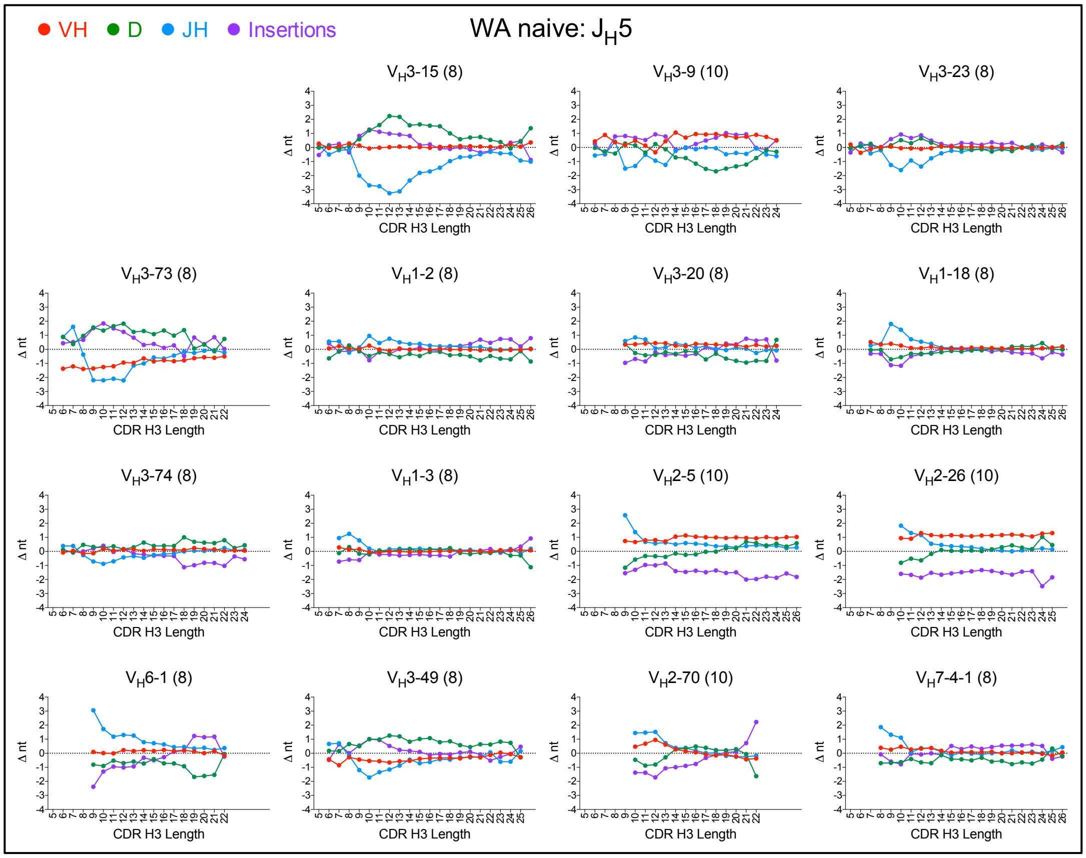

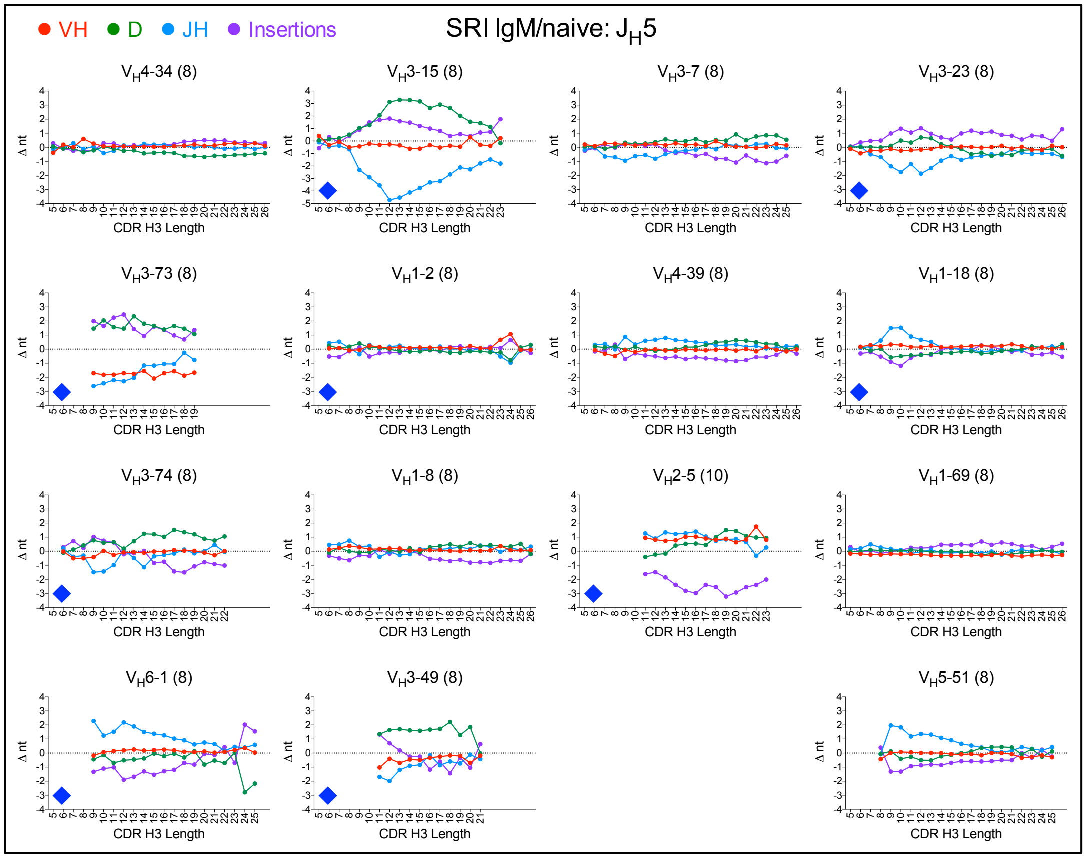

Supplementary Figure 16

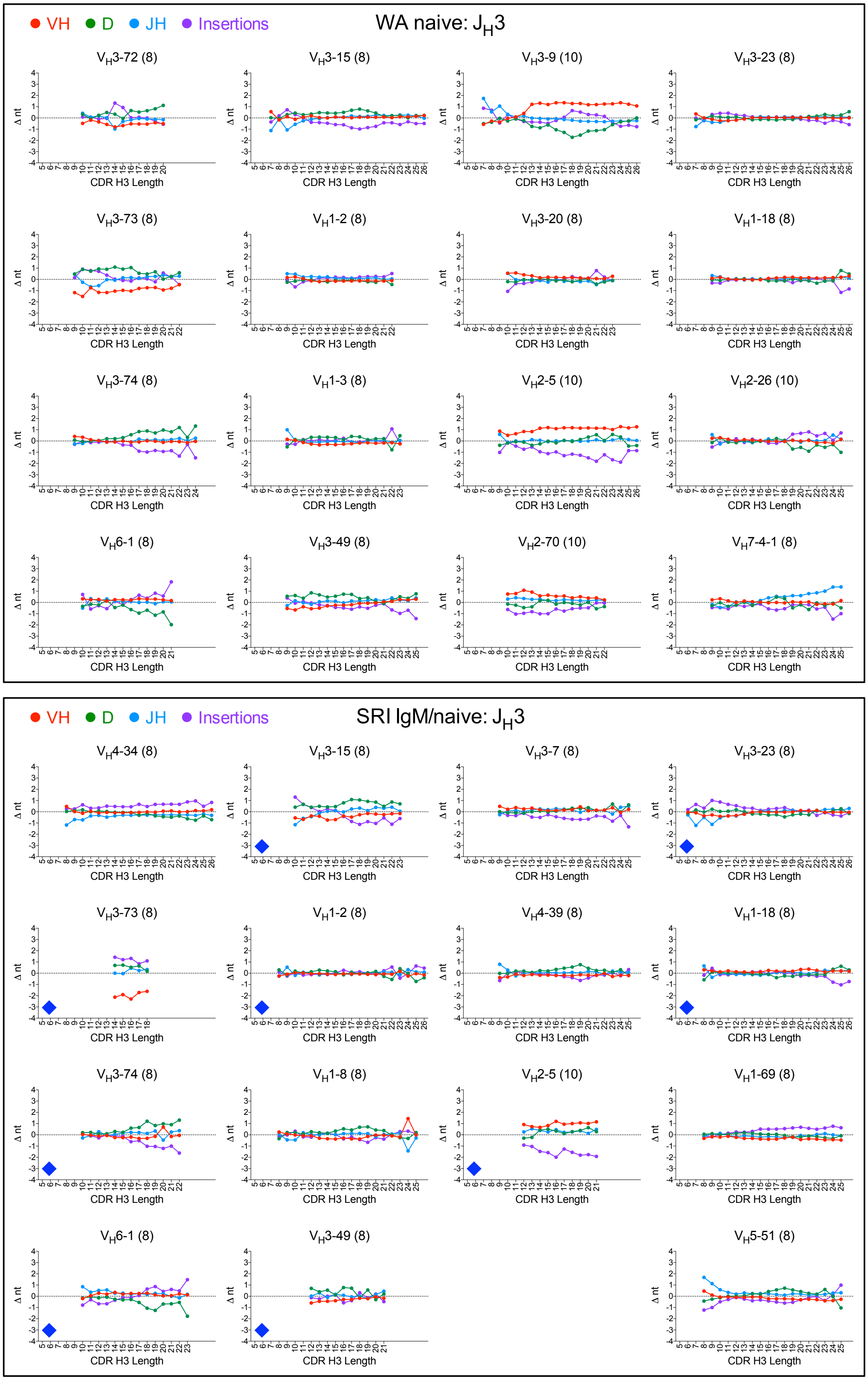

Supplementary Figure 16

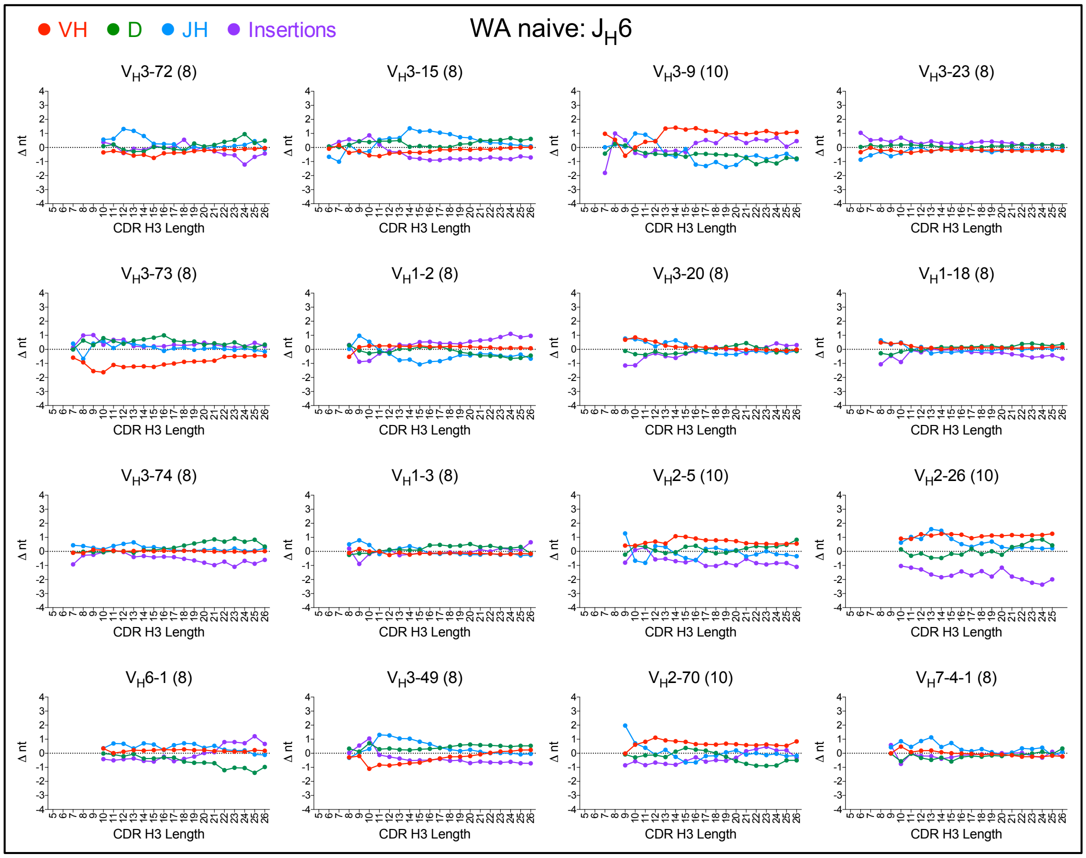

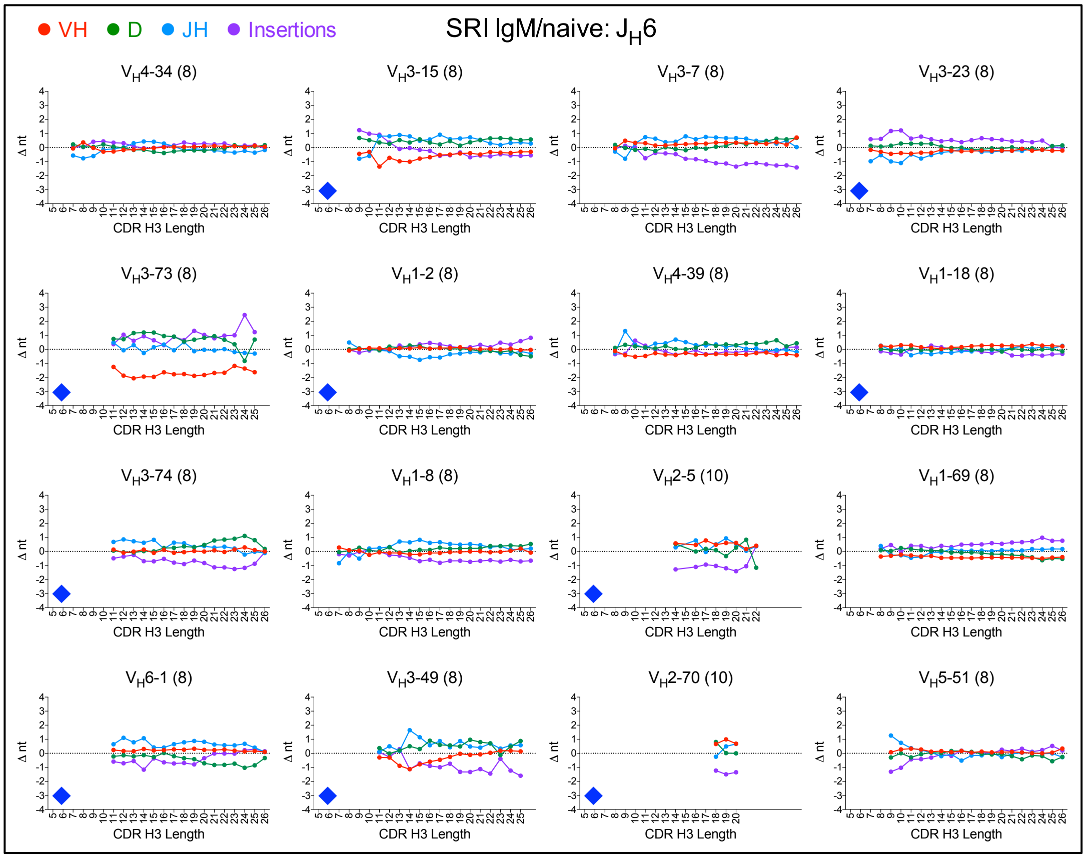

Supplementary Figure 16

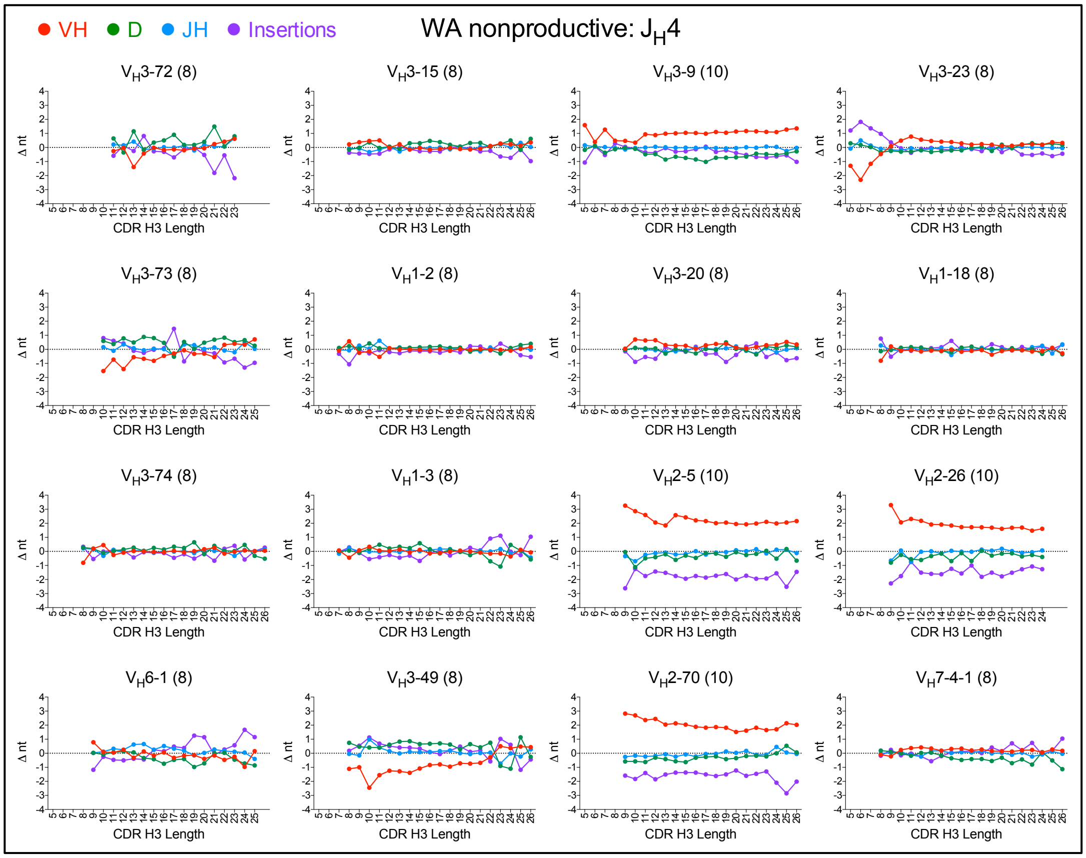

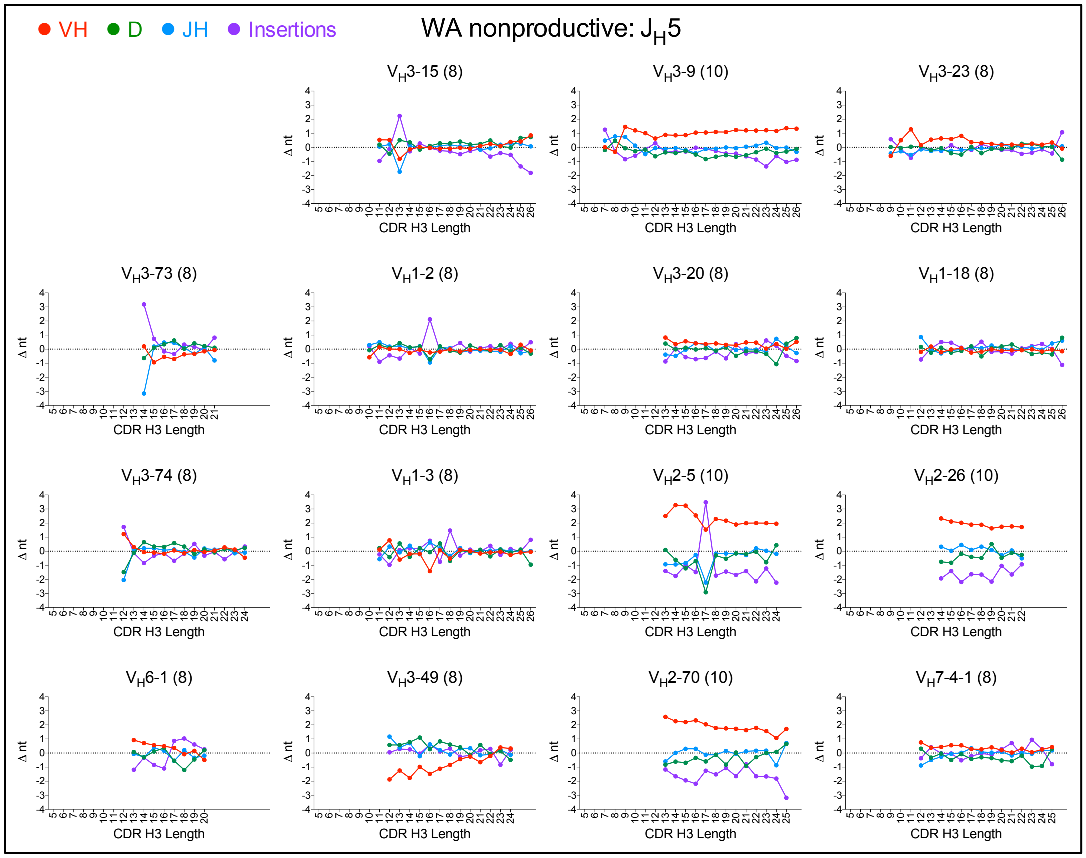

Supplementary Figure 16

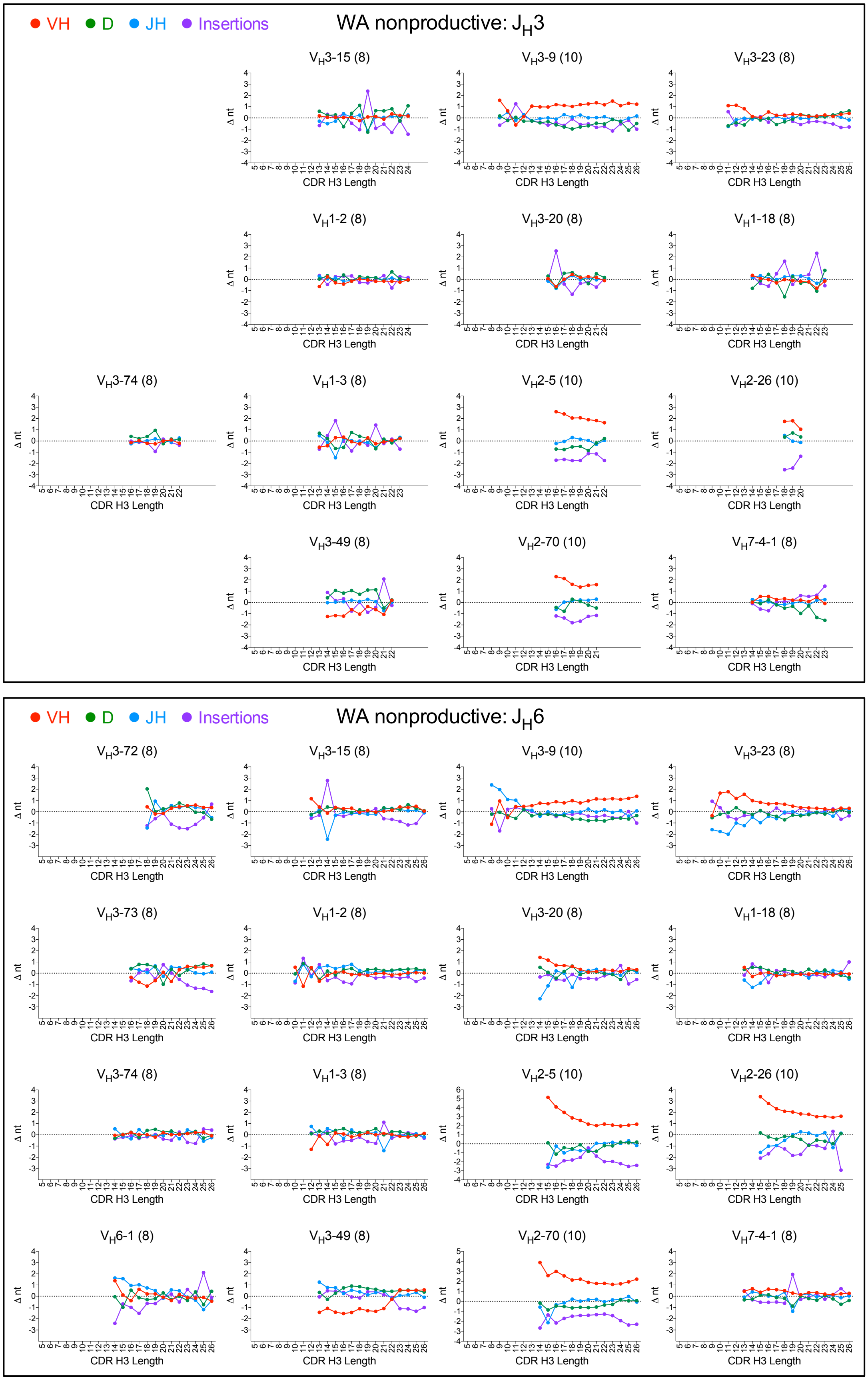

Supplementary Figure 16

Supplementary Figure 16. Deviations from average length for V_H, D_H, J_H and NP-regions (insertions) in clones segregated by V_H and J_H germline segments. Deviations are calculated by subtracting average values for each region from overall repertoire averages within donors. Data points show averages of at least 3 donors from each data set for V_H/J_H combinations with at least 60 counts. SRI panels highlighted by blue diamonds indicate V_H/J_H combinations also analyzed in the WA naive dataset. Error bars omitted for clarity. Note similarity of trends between WA and SRI naive datasets and lack of trends in WA unproductive sequences except for longer V_H sequences for V_H2 and V_H3-9, as expected, and a trend for shorter than expected lengths of nucleotide insertions in V_H2 sequences. See Supplementary notes for details.

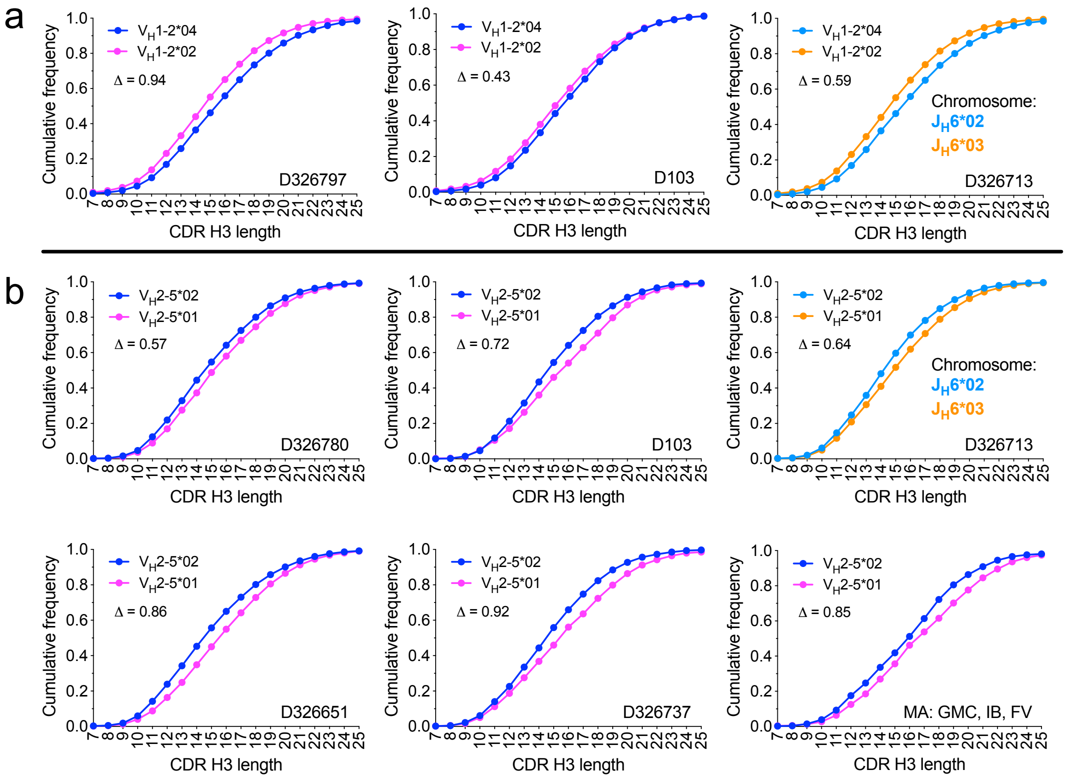

Supplementary Figure 17. CDR H3 length distributions associated with V_H1-2 (a, row 1) and V_H2-5 (b, rows 2 and 3) allelic variants in the SRI and MA datasets. All panels except the lower right are SRI dataset donors. Only heterozygous donors for these germline segments are shown, indicated in each panel. The 3 MA donors are heterozygous for V_H2-5 and were pooled in the lower right panel due to relatively low counts for each allele in individual donors. For SRI donors, only IgM/naive sequences are included. For the MA donors, only IgM sequences with up to 1 amino acid mutation in IMGT® positions 1 to 104 were included. Light blue and orange symbols in donor D326713 distributions indicate haplotypes based on J_H6 alleles associated with each V_H1-2 and V_H2-5 allele for donor D326713. Dark blue and magenta symbols indicate alleles for which haplotype analysis based on J_H6 alleles was not possible. Note the opposite biases of V_H1-2 and V_H2-5 alleles in the same chromosome for donor D326713, indicating that haplotype-associated variations of D germline segments do not directly determine allele-associated CDR H3 length biases. The difference in average CDR H3 length between alleles is shown in each panel. All CDR H3 distribution differences are statistically significant in a Mann-Whitney test ($P < 10^{-15}$, except V_H2-5 alleles of MA dataset, $P = 10^{-5}$). V_H2-5 alleles vary by Asn/Asp-59 and V_H1-2 alleles by Arg/Trp-75. Additional variations in the framework region 1 may occur but are not covered in the SRI dataset. Other allelic variants were not analyzed due to lack of sufficient number of heterozygous donors with same alleles or insufficient allele-specific sequence counts.

Germline	IMGT position 105	106	107	Bias group
IGHV3-66	A	R	(D)	Short
IGHV3-7	A	R	(D)	
IGHV3-72	A	R	(D)	
IGHV3-74	A	R	(D)	
IGHV6-1	A	R	(D)	
IGHV3-53	A	R	(D)	
IGHV3-73	T	R	(Q)	
IGHV3-15	T	T	(D)	
IGHV1-18	A	R	(D)	Long
IGHV1-69	A	R	(D)	
IGHV2-26	A	R	I	
IGHV2-70	A	R	I	
IGHV4-34	A	R	(G)	
IGHV1-24	A	T	(D)	
IGHV3-20	A	R	(D)	
IGHV3-30	A	R	(D)	
IGHV3-30-3	A	R	(D)	
IGHV3-33	A	R	(D)	
IGHV3-64	A	R	(D)	
IGHV3-64D	A	R	(D)	
IGHV3-23	A	K	(D)	
IGHV3-9	A	K	**D**	
IGHV5-51	A	R	(Q)	
IGHV1-2	A	R	(D)	Neutral
IGHV1-3	A	R	(D)	
IGHV2-5	A	H	**R**	
IGHV3-11	A	R	(D)	
IGHV3-21	A	R	(D)	
IGHV3-48	A	R	(D)	
IGHV3-49	T	R	(D)	
IGHV4-30-2	A	R	(D)	
IGHV4-30-4	A	R	(D)	
IGHV4-4	A	R	(D)	
IGHV4-61	A	R	(D)	
IGHV4-31	A	R	(D)	
IGHV4-59	A	R	(D)	
IGHV1-8	A	R	(G)	
IGHV4-39	A	R	(Q)	
IGHV7-4-1	A	R	X	

Supplementary Figure 18. CDR H3 residues encoded by V_H germline segments in the absence of nucleotide trimming. Residues in parentheses show partial codons and favored encoded residue in the absence of nucleotide trimming.

Supplementary Table

Supplementary Table 1. Datasets

	Dataset							
	CA	MA	TX			WA		SRI
Reference	1	2,3	4			5		6
Number of donors	3	3	3[a]	2 [a]	2 [a]	3		8
Cell type	IgG^{pos} B cells	PBMC	CD3^{neg} CD19^{pos} CD20^{pos} CD2^{pos} and CD3^{neg} CD19^{pos} CD20^{pos} CD27^{neg} B cells			CD3^{neg} CD19^{pos} CD27^{pos} and CD3neg CD19pos CD27neg B cells		PBMC
CD27 marker	NA[b]	NA	CD27^{neg}	CD27^{pos}	CD27^{pos}	CD27^{neg}	CD27^{pos}	NA
Source	cDNA	cDNA	cDNA			genomic DNA		cDNA
Sequencing platform	Illumina HiSeq2500	Illumina MiSeq	Illumina MiSeq			Illumina HiSeq		Illumina HiSeq2500
Isotype	IgG	IgG, IgA[c]	IgM, Null[d]	IgG, IgA	IgM	NA		IgG, IgM
B cell compartment in main text	CA AE	MA AE	TX naive	TX AE	TX AE IgM	WA naive	WA AE	SRI AE (IgG only) SRI naive (IgM)[h]
Chain sequences	$V_H + V_L$	V_H	$V_H + V_L$			V_H		V_H
V region coverage	Full-length	Full-length	Partial			Partial		Partial
V/J region parsing	Absolve, as published	IgBlast	IgBlast, As published			IMGT collaboration, as published		abstar, as published
Clonotype clustering	$V_H + V_L$	$V_H + J_H$	$V_H + V_L$			$V_H + J_H$[e]		$V_H + J_H$
Clonotype clustering CDR H3 aa identity[f]	60%	60%	60%			100%		100%
Raw counts	68,727	233,724	55,210	81,786	26,447	2.2 x 10^7	1.9 x 10^7	1.9 x 10^8
Unique clonotypes[g]	63,503	63,051	52,993	67,158	22,616	1.7 x 10^7	8.3 x 10^6	AE: 6.3 x 10^6 Naive[h]: 1.7 x 10^7
Unproductive sequences	NA	NA	NA			3.1 x 10^6	Not used	NA

[a] Dataset with 3 donors, only 2 of which were processed for CD27^{pos} B cells.

[b] Not available.

[c] IgM sequences were not included in analyses for the MA dataset.

[d] "Null" isotype sequences in the CD27neg compartment were assumed to be IgM.

[e] Sequences with undefined V_H or J_H germlines grouped by clonotype as described in methods.

[f] Average 60% amino acid identity across CDR H3 lengths achieved by using a nominal 57% identity threshold.

[g] Number of sequences after filtering for clonotypes as described in methods.

[h] IgM/naive sequences in the SRI dataset defined as IgM sequences without amino acid mutations between IMGT® Cys-23 and Cys-104.

Supplementary Notes

Biases in D_H segment and N-region lengths within CDR H3 sequences as a function of V_H and J_H germline segment use. Parsing of D_H segments in the WA naive and SRI IgM sequences with no mutations in the V_H region between Cys-23 and Cys-104, as a proxy for naive sequences, was done using Blastafter removing the sequences corresponding to V_H and J_H regions from CDR H3 sequences. The samples from the D1Nb subset were also included for D_H segment parsing, removing redundant sequences as described in Methods. An identity of 100% over a span of at least 5 contiguous nucleotides was required for D_H germline segment matches. The identity of the DH segments for each hit were not considered, only the length of the germline segment in the germline. This was done due to parsing uncertainties, especially for the shorter matches. However, each match length represents the longest possible length within the set of D_H segments. In addition, all datasets were parsed the same way, controlling for biases in the parsing procedures.

We determined whether the observed J_H segment length biases in CDR H3 sequences of specific lengths are an indirect consequence of biases elsewhere in CDR H3, such as length of V_H and D_H sequences and number of N-region and palindromic nucleotide insertions (NP-region) flanking the D_H region in CDR H3. Naive sequences from the 3 donors of the WA dataset and SRI IgM/naive sequences from 3 of the donors with higher number of sequences in the SRI dataset were parsed for V_H, J_H, D_H and NP-region lengths within CDR H3. In general, no obvious differences on the prevalence of D_H germline segments of different lengths within the germline were observed in association with different V_H and the J_H4 and J_H5 germline segments that would account for J_H length biases (Supplementary Fig. 14). One possible exception is V_H6-1 which was associated with shorter D_H germline segments. In addition, the number of nucleotides that V_H can directly contribute to CDR H3 did not correlate with J_H length biases (Supplementary Fig. 15 and 16, red and blue lines and symbols). However, different classes of biases in the lengths of D_H

segments and NP-regions were observed for different V_H/J_H combinations, even for clones with the same V_H germline segment (Supplementary Fig. 15 and 16, green lines and symbols). Biases had similar trends in the WA and SRI datasets, with differences between datasets observed mostly in the magnitude of the biases. No similar biases were observed in the nonproductive WA sequences with exception of differences in average V_H-derived sequence lengths associated with V_H germline segment length and a generally shorter NP-region length in V_H2 clones (Supplementary Fig. 16), indicating that the observed D_H and NP-region length biases are mostly selected in naive repertoire maturation.

Different classes of junctional biases were observed for different V_H/J_H combinations (Supplementary Fig. 15 and 16). In several V_H/J_H combinations, J_H length biases were inversely correlated with both D_H segment and NP-region length biases including, for instance, V_H3-23/J_H5, V_H3-15/J_H4 and V_H3-73/J_H4/J_H5 combinations. In these cases, the factor determining junctional biases is likely to be the J_H segment, with the other junctional components compensating for the J_H biases. In V_H3-74/J_H4, D_H length biases are compensated by both NP-region and J_H lengths, suggesting the D_H segments as the bias determinant. In other cases, such as V_H3-15/J_H4, V_H3-7/J_H4 and V_H3-9/J_H4, D_H and NP-region lengths were inversely correlated with each other without correlations with J_H segment length across the CDR H3 length spectrum. In V_H2 sequences, the reduction in NP-region length observed in nonproductive sequences is maintained in the naive repertoire and compensates for the longer V_H sequences. However, as the J_H segments of nonproductive V_H2 sequences do not appear to be biased relative to the overall repertoire, the observed J_H4 and J_H5 trimming biases associated with V_H2 naive sequences are presumably due to J_H trimming rather than NP-region selection. Overall, the results indicate different classes of biases in D_H segment, N-region and J_H lengths within CDR H3 of naive sequences that vary among V_H/J_H germline segment combinations.

Supplementary References

1. Goldstein, L. D. et al. Massively parallel single-cell B-cell receptor sequencing enables rapid discovery of diverse antigen-reactive antibodies. *Commun Biol* 2, 304 (2019).

2 Busse, C. Dynamics of the human antibody repertoire after influenza vaccination. NCBI BioProject Database, https://www.ncbi.nlm.nih.gov/bioproject/PRJNA349143 (2016).

3 Laserson, U. et al. High-resolution antibody dynamics of vaccine-induced immune responses. *Proc Natl Acad Sci U S A* 111, 4928-4933 (2014).

4 DeKosky, B. J. et al. In-depth determination and analysis of the human paired heavy- and light-chain antibody repertoire. *Nat Med* 21, 86-91 (2015).

5 DeWitt, W. S. et al. A Public Database of Memory and Naive B-Cell Receptor Sequences. PLoS One 11, e0160853 (2016).

6 Briney, B., Inderbitzin, A., Joyce, C. & Burton, D. R. Commonality despite exceptional diversity in the baseline human antibody repertoire. *Nature* 566, 393-397 (2019)
