# Supplementary material for: Dynamics of heavy chain junctional length biases in antibody repertoires
Source: Commun Biol. 2020 May 1;3:207. doi: 10.1038/s42003-020-0931-3 (PMC7195405; doi:10.1038/s42003-020-0931-3)
Supplement: Supplementary file 5 — Description of Additional Supplementary Files [file 42003_2020_931_MOESM5_ESM.pdf]

Supplementary Data Legends

Supplementary Data includes 5 Tables:

Supplementary Data 1 – Clonotype counts with each germline segment in the AE ($CD27^{pos}$/IgG/IgA B cells) in individual donors of the TX, CA, MA, WA and SRI datasets and combined counts per dataset. Values lower than 80 in individual donors and 240 in the combined donors for each dataset or that were not used due to relatively low counts within each dataset are highlighted. Repertoire fraction indicates the fraction of the repertoire that includes the shown germline segments for each donor or dataset.

Supplementary Data 2 – Clonotype counts with each germline segment in the Naive ($CD27^{neg}$ B cells) in individual donors of the TX and WA datasets and combined counts per dataset. Values lower than 80 in individual donors and 240 in the combined donors for each dataset or that were not used due to relatively low counts within each dataset are highlighted. Repertoire fraction indicates the fraction of the repertoire that includes the shown germline segments for each donor or dataset.

Supplementary Data 3 – Clonotype counts with each germline segment in the TX AE IgM ($CD27^{pos}$, IgM B cells) in individual donors and the combined dataset. Values lower than 80 in individual donors and 240 in the combined donors for each dataset are highlighted. Repertoire fraction indicates the fraction of the repertoire that includes the shown germline segments for each donor or dataset.

Supplementary Data 4 – WA dataset germline segment classification ambiguity. The number of clonotypes with unambiguous and ambiguous germline segment classification in the AE and naive compartments of the WA dataset are shown. Unambiguous and ambiguous germline segment classification refers to sequences with only one or more than one possible germline segment classification in the originally published dataset germline segment call in the originally published dataset respectively.

Germline segments with less than 10% ambiguity and relatively high counts were selected for analysis.

Supplementary Data 5 – MA dataset source. Accession numbers in the Sequence Read Archive (SRA) database and details for each of the subsets of the MA dataset used are shown.

Supplementary Data 6: Figure Data file includes 27 files with the data in plots of Figures 1 to 6 and Supplementary Figures 1 to 13 and 15 to 17.
